# The role of mentoring, supervision, coaching, teaching and instruction on professional identity formation: a systematic scoping review

**DOI:** 10.1186/s12909-022-03589-z

**Published:** 2022-07-08

**Authors:** Rachelle Qi En Toh, Kai Kee Koh, Jun Kiat Lua, Ruth Si Man Wong, Elaine Li Ying Quah, Aiswarya Panda, Chong Yao Ho, Nicole-Ann Lim, Yun Ting Ong, Keith Zi Yuan Chua, Victoria Wen Wei Ng, Sabine Lauren Chyi Hui Wong, Luke Yu Xuan Yeo, Sin Yee See, Jolene Jing Yin Teo, Yaazhini Renganathan, Annelissa Mien Chew Chin, Lalit Kumar Radha Krishna

**Affiliations:** 1 Yong Loo Lin School of Medicine, National University Singapore, 1E Kent Ridge Road, NUHS Tower Block, Level 11, Singapore, 119228 Singapore; 2 Division of Palliative and Supportive Care, National Cancer Centre Singapore, 11 Hospital Crescent, Singapore, 16961 Singapore; 3 Medical Library, National University of Singapore Libraries, National University of Singapore Blk MD6, Centre for Translational Medicine, 14 Medical Dr, #05-01, Singapore, 117599 Singapore; 4 Division of Cancer Education, National Cancer Centre Singapore, 11 Hospital Crescent, Singapore, 16961 Singapore; 5 Duke-NUS Medical School, 8 College Road, Singapore, 169857 Singapore; 6 Palliative Care Institute Liverpool, Academic Palliative & End of Life Care Centre, University of Liverpool, 200 London Rd, Liverpool, L3 9TA UK; 7 Centre for Biomedical Ethics, National University of Singapore, Blk MD11, 10 Medical Drive, #02-03, Singapore, 117597 Singapore; 8 PalC, The Palliative Care Centre for Excellence in Research and Education, PalC c/o Dover Park Hospice, 10 Jalan Tan Tock Seng, Singapore, 308436 Singapore

**Keywords:** Mentoring, Supervision, Coaching, Teaching, Instruction, Professional Identity Formation, Communities of Practice

## Abstract

**Background:**

Mentoring’s pivotal role in nurturing professional identity formation (PIF) owes much to its combined use with supervision, coaching, tutoring, instruction, and teaching. However the effects of this combination called the ‘mentoring umbrella’ remains poorly understood. This systematic scoping review thus aims to map current understanding.

**Methods:**

A Systematic Evidence-Based Approach guided systematic scoping review seeks to map current understanding of the ‘mentoring umbrella’ and its effects on PIF on medical students and physicians in training. It is hoped that insights provided will guide structuring, support and oversight of the ‘mentoring umbrella’ in nurturing PIF. Articles published between 2000 and 2021 in PubMed, Scopus, ERIC and the Cochrane databases were scrutinised. The included articles were concurrently summarised and tabulated and concurrently analysed using content and thematic analysis and tabulated. The themes and categories identified were compared with the summaries of the included articles to create accountable and reproducible domains that guide the discussion.

**Results:**

A total of 12201 abstracts were reviewed, 657 full text articles evaluated, and 207 articles included. The three domains identified were definitions; impact on PIF; and enablers and barriers. The mentoring umbrella shapes PIF in 3 stages and builds a cognitive base of essential knowledge, skills and professional attitudes. The cognitive base informs thinking, conduct and opinions in early supervised clinical exposure in Communities of practice (COP). The COPs’ individualised approach to the inculcation of desired professional characteristics, goals, values, principles and beliefs reshapes the individual’s identity whilst the socialisation process sees to their integration into current identities.

**Conclusion:**

The mentoring umbrella’s provides personalised longitudinal support in the COP and socialisation process. Understanding it is key to addressing difficulties faced and ensuring holistic and timely support.

**Supplementary Information:**

The online version contains supplementary material available at 10.1186/s12909-022-03589-z.

## Introduction

Mentoring plays a critical role in nurturing professional identity formation (henceforth PIF) or helping medical students and physicians (henceforth physicians in training) “think, act and feel like physicians” [1]. This role is premised on the notion that mentoring’s personalised, longitudinal and holistic support helps physicians in training integrate the relevant professional values, beliefs, expectations, standards, codes of conduct, culture and principles of the medical profession into their individual identities [2]. However, efforts to understand mentoring’s precise role in PIF has been limited by the presence of a variety of different forms of mentoring [3–5] and its conflation with distinct practices such as role modelling, supervision, coaching, tutoring, teaching and instruction [6]. Two new developments promise to change this impasse and offer new insights into mentoring’s role in PIF.

The first is evidence that role modelling, supervision, coaching, tutoring, teaching and instruction take on characteristics that liken them to mentoring when applied in a longitudinal manner to enduring and personalised educational relationships [7]. Krishna et al. (2019) suggest overlaps with traditionally understood concepts of mentoring, allowing these approaches to be considered part of a larger concept called the ‘mentoring umbrella’.

The second is the notion that professional identity is part of a larger concept of identity and that self-concepts of identity are intimately related and informed by self-concepts of personhood or “what makes you, you” [8]. As such, the influence of effective mentoring on the PIF of physicians in training may be understood through the lens of personhood. This is especially useful amidst evidence that evaluations of self-concepts of personhood did allow for better appreciation of changing notions of identity particularly when current tools fail to effectively evaluate such evolving concepts.

### Ring theory of personhood

**Fig. 1:**
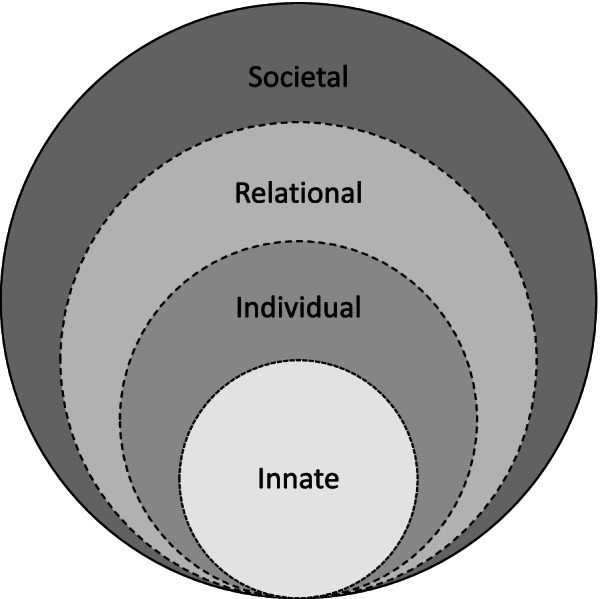
The Ring Theory of Personhood (RToP)

Radha Krishna and Alsuwaigh [9]’s Ring Theory of Personhood (RToP) is a clinically evidenced tool that maps changing concepts of personhood and captures evolving notions of identity. The RToP suggests that personhood is comprised of the Innate, Individual, Relational and Societal Rings (Fig. 1) [10–12]. With each ring encapsulating the values, beliefs, and principles of the particular aspect of the clinician’s identity, each ring also represents the corresponding aspects of identity (Fig. 1) [12, 13].

It is suggested that better understanding of these values, beliefs, and principles will reveal how a physician in training’s views their roles, responsibilities, and place within a team, family unit, professional community, and society and provide insights into the physician in training’s thinking, conduct and coping in the face of different situational, environmental, and/or relational influences [14–22].

At the core of the Ring Theory is the Innate Ring that houses the individual’s spiritual, religious and/or theist beliefs, values, moral ideals, and ethical principles. These are shaped by the individual’s demographical and historical features such as the ethnicity, culture, religion, family unit, gender, society, country, and social group they were born into. These considerations influence the individual’s Innate Identity and their thinking, goals, motivations, and actions.

The Individual Ring represents conscious function which includes the ability to think, feel, communicate, carry out actions, and interact with the environment. The Individual Ring houses the individual’s values, beliefs, principles, biases, preferences, thoughts, emotions, experiences, decision making and personality which shape Individual Identity.

The Individual Ring also acts to balance the thinking, goals, motivations, and actions drawn from the Innate, Relational and Societal Identities.

The Relational Ring consists of personal relationships deemed to be important to the individual, and the values and beliefs that stem from and inform these relationships. The Societal Ring contains societal, religious, professional, and legal expectations set out in the individual’s society to guide and police conduct. One’s professional identity resides here.

These identities may come into conflict when professional involvement in cases such as those involving palliative sedation, withdrawal or withholding of treatment, termination of pregnancy or familial determination arise.

### Structured ‘mentoring umbrella’ approach

A structured ‘mentoring umbrella’ approach replete with a combination of mentoring, supervision, coaching, tutoring, teaching and instruction may be key to structuring and guiding this professional identity formation process. Indeed, Krishna et al. (2018) suggest that the most significant role of this holistic approach is its ability to support students, residents and junior doctors during periods of negotiation where new experiences and obstacles are either accepted, adapted to fit their particular circumstances or needs (compromised) or rejected [23]. Kuek, Ngiam [24], Ho, Kow [11], Ngiam, Ong [25], Chan, Chia [10] and Huang, Toh [13] suggest that ‘conflict’ sees the beliefs, values and principles housed in each of the four rings in ‘tension’ with professional norms and responsibilities introduced to each ring. If the ‘tension’ persists, dyssynchrony or identity dissonance arises [24]. This may increase the risk of burnout and a loss of interest in the profession [26–32]. Effectively supporting the processing and resolution of dyssynchrony will attenuate these risks.

With Sarraf-Yazdi et al. (2021) suggesting that mentoring helps each of the four identities adapt to the inculcation of these new professional values and responsibilities, evaluating elements of the ‘mentoring umbrella’ more closely may clarify its role within any proposed PIF focused training program.

## Methods

A Systematic Evidence-Based Approach guided systematic scoping review (henceforth SSR in SEBA) is used to map what is known about the effects of mentoring, supervision, coaching, tutoring, teaching and instruction upon PIF [33–36]. Given its broader scope, we aim to study role modelling’s impact on PIF in a separate review.

**Fig. 2:**
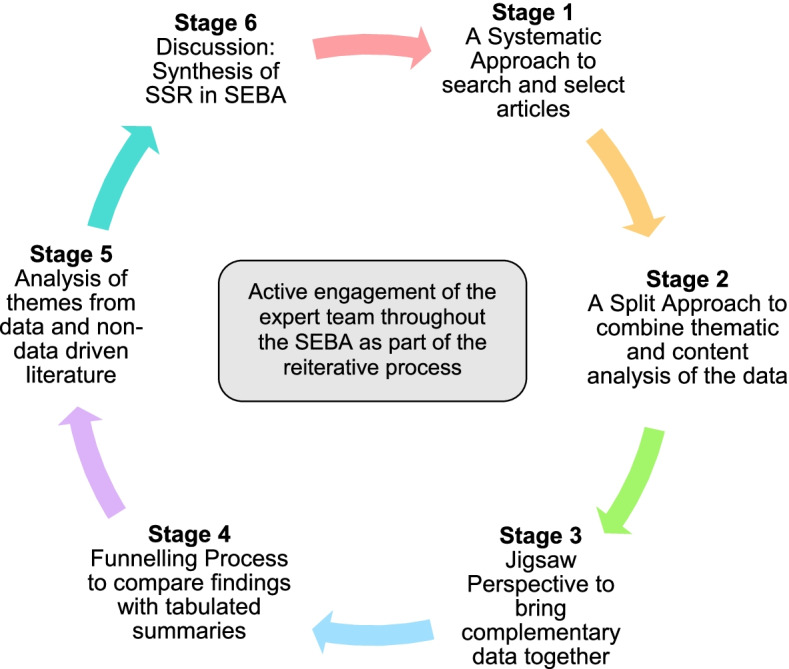
The SEBA Process

This SSR in SEBA is overseen by an expert team comprised of medical librarians from the Yong Loo Lin School of Medicine (YLLSoM) and the National Cancer Centre Singapore (NCCS), and local educational experts and clinicians at NCCS, the Palliative Care Institute Liverpool, YLLSoM and Duke-NUS Medical School who guide, oversee and support all stages of SEBA to enhance the reproducibility and accountability of the process [37–49] (Fig. 2).

### Stage 1 of SEBA: systematic approach

#### Determining the title

The research and expert teams set out the overarching goals, study population, context and remediation programs to be evaluated.

#### Inclusion criteria

The PICOS format was used to guide the inclusion criteria Table 1.

**Table 1:** PICOs, inclusion criteria and exclusion criteria applied to database search

PICOS	Inclusion Criteria	Exclusion Criteria
**Population**	• Junior physicians, residents, and medical students	Allied health specialties such as dietetics, nursing, psychology, chiropractic, midwifery, social work Specialists, consultants, attendings and physicians not in training programs Non-medical specialties such as clinical and translational science, veterinary, dentistry
**Intervention**	• All forms of mentoring and o Mentoring processes o Mentor factors o Mentee factors o Mentoring relationship o Host organization o Outcomes of mentoring o Barriers to mentoring o Mentoring structure o Mentoring framework o Mentoring culture o Mentoring environment • Educational roles of mentoring: Supervision, coaching, role-modelling, teaching, and tutoring	
**Comparison**	• Comparisons accounts of mentoring between mentoring programs, editorials, and perspective, reflective, narratives and opinions pieces	
**Outcome**	• Personal outcomes of mentoring such as values, beliefs, identity as a medical professional etc • Professional development outcomes such as on career choices (including academia positions/careers)	• Papers that did not discuss impact of mentoring on personal or professional development outcomes
**Study design**	• All study designs are included o Descriptive papers o Qualitative, quantitative, and mixed study methods o Systematic review, literature reviews, and narrative reviews • Perspectives, opinion, commentary pieces, and editorials • Year: 1^st^ January 2000 to 31^st^ December 2020	

#### Identifying the research question

To identify the research question, the expert and research teams were guided by the Population, Intervention, Comparison, Outcome and Study Design (PICOS) elements of the inclusion criteria [50, 51]. The primary research question was identified as follows: “*What is known about the effect of mentoring, supervision, coaching, tutoring, teaching and instruction on professional identity formation amongst medical students, residents and junior doctors?*”

#### Searching

In keeping with Pham, Rajić [52]’s recommendations on ensuring a viable and sustainable research process, the research team confined the searches to articles published between 1^st^ January 2000 to 31^st^ December 2020 to account for prevailing manpower and time constraints. Additional ‘snowballing’ of references of the included articles ensured a more comprehensive review of the articles [53].

#### Extracting and charting

Using an abstract screening tool, the research team independently reviewed abstracts to be included and employed ‘negotiated consensual validation’ to achieve consensus on the final list of articles to be included [54].

#### Stage 2 of SEBA: split approach

The split approach [55] sees concurrent analysis of the included articles by three independent teams. The first team summarised and tabulated the articles in keeping with recommendations drawn from RAMESES publication standards by Wong, Greenhalgh [56] and “*Guidance on the conduct of narrative synthesis in systematic reviews*” by Popay, Roberts [57]. The second team used the approach to thematic analysis by Braun and Clarke [58] to find meaning and patterns in the data whilst the third team employed the approach to directed content analysis by Hsieh and Shannon [59] to “*identifying and operationalizing a priori coding categories*” from *“The Development of Professional Identity” by* Cruess and Cruess [2]*.* ‘Negotiated consensual validation’ was used as a means of peer debrief in all three teams to further enhance the validity of the findings [60].

#### Stage 3 of SEBA: jigsaw perspective

The Jigsaw Perspective employs Phases 4 to 6 of France et al. [61]’s adaptation of Noblit et al. [62]’s seven phases of meta- ethnographic approach to view the themes and categories as pieces of a jigsaw puzzle where overlapping/complementary pieces are combined to create a bigger piece of the puzzle referred to as themes/categories. This process would see themes and subthemes compared with the categories and subcategories identified. These similarities were verified by comparing the codes contained within them. If they are complementary in nature, then the subtheme and subcategory are combined to create a bigger piece of the jigsaw puzzle Table 2.

**Table 2:** Subthemes and subcategories

Subthemes	Subcategories
The Impact of Mentoring, Supervision, Coaching, Teaching and Instruction on Personhood: RToP	Innate Ring; Individual Ring; Relational Ring; Societal Ring
Barriers and Enablers	Mentoring and its Roles; Communities; Learners; Institutions; Others

#### Stage 4 of SEBA: Funnelling

Themes/categories were compared with the tabulated summaries (Additional file 1: Appendix A). The funnelled domains created from this process forms the basis of the discussion’s ‘line of argument’.

## Results

**Fig. 3:**
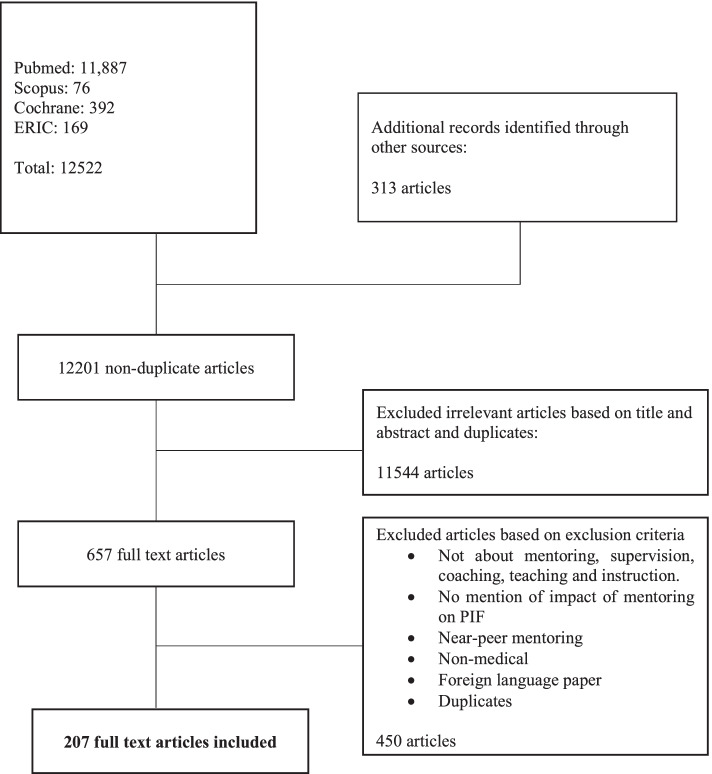
PRISMA flow chart

A total of 12201 abstracts were reviewed, 657 full text articles evaluated, and 207 articles included and coded. A total of 176 of the 207 articles were data-driven while 31 articles were opinion driven (commentaries, editorials, letters, perspectives, reflections) (Fig. 3). Of the data driven articles, 55 were quantitative studies, 75 were qualitative studies, 33 were mixed studies, and 13 were literature and systematic reviews.

There were 163 articles on mentoring, 26 articles on supervision, 18 articles on coaching, 46 articles on teaching and 8 articles on instruction. There were a few articles that covered a variety of forms of mentoring.

### Themes and categories identified

Scrutiny of the themes and categories from thematic and content analysis were consistent with one another. To avoid repetition, we discuss the themes identified using both approaches in tandem. The funnelled domains identified were:

1. A definition for each of the elements of the mentoring umbrella
2. How each element within the mentoring environment impacts PIF
3. Enablers and barriers to mentoring, *supervision, coaching, teaching and instruction’s effects on PIF.*

#### Domain 1

Defining mentoring, *supervision, coaching, teaching and instruction.* From the included articles it is possible to delineate an understanding of *mentoring supervision, coaching, teaching and instruction*. These are summarised in Table 3.

**Table 3:** Definitions and descriptors of mentoring and its roles

Mentoring	▪ “Dynamic, context dependent, goal sensitive, mutually beneficial relationship between an experienced clinician and junior clinicians and or undergraduates that is focused upon advancing the development of the mentee.” [63]
Teaching	▪ Impart knowledge and guide studies by precept, examples or experience [63]. ▪ Teaching in the clinical environment is defined as teaching and learning focused on, and usually directly involving, patients and their problems [64]
Coaching	▪ Coaching is an inherently creative activity of bringing forth knowledge, wisdom, and insight [65]. ▪ A coach works with a student to continually improve his/her performance, usually on areas that the student deems weak [66]. ▪ The coaching process involves asking questions [66], listening deeply [65], keenly observing [65, 67], evaluating and identifying gaps [68], providing specific and concrete feedback [67, 68], creating goals, exploring solutions, and holding the individual accountable [68], supporting reflection [65, 66, 69], setting goals [69], developing a comprehensive study plan [69] and ensuring a commitment to learning [65]. ▪ Coaching can also improve their emotional intelligence, durability, wellbeing, and resilience [66]. ▪ In medical education, two main types of coaching have been described [68]: • Coaching in clinical skills: coach directly observes the learner in the clinical setting and then engages in the coaching process for the improvement of a specific skill such as procedural training [68] • Academic coaching: coaches guide learners to achieve their fullest potential by indirectly evaluating performance via review of objective assessments [68]: o (a) self-reflection; o (b) specific, measurable, achievable, relevant and time-based (SMART) goal setting; o (c) the development of comprehensive study plans with deliberate use of effective learning strategies including spaced retrieval practice and elaboration, and o (d) self-care. • Teaching faculty members supported the streamlined, collaborative approach. Academic coaches offered timely oversight and early identification of students requiring support [69].
Instruction	▪ None of the articles defined instruction. ▪ According to the UNESCO International Bureau of Education, instruction is defined as: *“*The creation and implementation of purposefully developed plans for guiding the process by which learners gain knowledge and understanding, and develop skills, attitudes, appreciations and values.” [70]
Supervision	▪ Supervision may be seen “as an intervention, a working alliance, a method, a process and a professional activity.” [71] ▪ Supervision may be conceived of as “…a joint endeavour in which a practitioner with the help of a supervisor, attends to their clients, themselves as part of their client practitioner relationships and the wider systemic context, and by so doing improves the quality of their work, transforms their client relationships, continuously develops themselves, their practice and the wider profession.” [72] ▪ Clinical supervision has been defined as the “provision of guidance and feedback on matters of personal, professional and educational development in the context of a trainee’s experience of providing safe and appropriate patient care” [73].

#### Domain

Impact of *mentoring, supervision, coaching, teaching and instruction impact PIF*

To effectively evaluate the impact of the elements of the mentoring umbrella on PIF, we discuss each of them in turn through the lens of the RtoP.

##### Mentoring

Mentoring supports minority groups with guidance and networking opportunities [74–76] and helps female mentees balance their career demands and family responsibilities [77–81], underlying its role in the Innate Ring.

In the Individual Ring mentoring helps support the mentee’s career, personal, research and academic goals, beliefs, values, and motivations by boosting confidence [80, 82–89], discipline [89] resilience [90, 91], and self-efficacy of the mentee [86]. Mentoring also supports reflective practice [74, 87, 92, 93] which increases career satisfaction [94], and boosts work-life balance [95–98], and reduces burnout and disillusionment [99].

Within the Relational Ring mentoring is credited with enhancing parenting skills [100], and improving relationships with family members [93, 98]. In the Societal Ring, mentoring improves networking [101], sponsorship [102], interprofessional practice [98] and patient interactions [103].

##### Supervision

Supervision’s effect on the Individual Ring includes increasing interest in a particular field [104–107], influencing career decisions [104, 105, 107, 108], boosting personal development/growth [71, 106, 109–112] and personal skills [110] and improves career satisfaction [110, 113]. In the Societal Ring, supervision enhances academic [3, 110, 114–117], research [104, 110], decision making skills [109] and clinical [71, 73, 107, 109, 114, 116–122] competencies and supports socialisation of a professional identity [115, 118, 123, 124].

##### Teaching

In the Individual Ring, teaching improves interest in a particular field [104–106, 125–127], influences career decisions [104, 105], nurtures personal development/growth [106, 110, 128–139] and boosts career satisfaction [110, 139, 140]. In the Societal Ring, teaching increases academic [74, 110, 127, 132, 135, 141] clinical [118, 119, 128, 129, 131–135, 138, 141–153] and research [104, 110, 126, 154–156] competencies [110, 118, 119, 130, 136, 137, 139, 143, 149, 155, 157, 158].

Within the Societal Ring, teaching advances networking [74, 141], career goals [110, 126, 151] and research outputs [104, 110, 126, 151], improves interprofessional working [104, 118, 135, 141, 149], patient interactions [118, 128, 131, 133–135, 138, 141, 142, 146, 147, 149, 157, 159], social identity and a sense of community [141].

##### Coaching

In the Individual Ring, coaching influences career decisions [104], boosts personal development/growth [65, 66, 68, 69, 110, 139, 160–162] and career satisfaction [66, 110].

In addition, coaching improves academic [65, 68, 69, 110, 117, 162, 163] clinical [65, 117, 160, 164–166] and research [104, 110] competencies [3, 110, 139, 160, 164, 165] in the Societal Ring.

##### Instruction

In the Individual Ring, instruction improves skills [133] and time management [167] and in the Societal Ring it improves clinical competencies [133, 147, 149, 157, 167, 168] and interactions with patients [133, 147, 149, 157] and fellow professionals [149, 167].

#### Domain 3

There are factors that enhance (enablers) and hinder (barriers) the impact of mentoring, supervision, coaching, teaching and instruction upon PIF. These may be divided into mentee, mentor and institutional factors.

##### Mentee-related

Mentee related factors influencing the efficacy of the mentoring umbrella include being motivated, proactive, invested in the mentoring process and relationships, reflective, willingness to take feedback and make necessary adaptations and assign sufficient time to training [35, 82, 83, 97, 102, 118, 169–176].

##### Mentor-related

Mentor related factors consider all the roles played under the aegis of the mentoring umbrella. These include being motivated and invested in mentoring, having the abilities, availabilities and experience required, possessing good listening and communication skills, a commitment to self-improvement and learning, being open to feedback and learning from the mentee, being able to provide holistic and longitudinal support and understanding and abiding by the expectations and standards of practice expected of a mentor [74, 78, 80, 83, 86, 87, 89, 95, 170, 177, 178].

##### Institution-related

The host organization plays a critical role in matching, training, supporting and structuring the training process. The implementation of protected time and formal recognition of participation in mentoring help maintain motivation.

The host organization also plays a part in establishing clear codes of conduct, roles and responsibilities and expectations of all stakeholders, structuring the mentoring process, providing it a formal place in the curriculum, assessing and overseeing the program [3, 80, 83, 84, 87, 89, 90, 97, 100, 118, 170, 174]. This is especially important when considering the hidden and informal curriculum influence workplace culture; career choice; impact upon the mentoring environment [67, 86, 90, 93, 100, 102, 113, 130, 179, 180]; and acknowledgment and personal and team investment in the efforts of the mentoring umbrella [71, 77, 82, 118, 130, 181].

#### Stage 5 of SEBA: analysis of evidence-based and Non-data driven Literature

The themes drawn from evidenced-based publications were compared with those from non-data based articles (grey literature, opinion, perspectives, editorial, letters) found that the themes from both groups to be similar and non-data based articles did not bias the analysis untowardly.

Most of the included articles were data-driven (175 out of 207) whilst the remaining articles were non-data-based articles (grey literature, commentaries, opinion, perspectives, editorial, letters). Despite non-data-based articles forming a small minority of articles, we examined themes drawn from the non-data-driven publications and compared them with those from data-based articles (grey literature, opinion, perspectives, editorial, letters). This process revealed similarities between the two groups suggesting that non-data-based articles did not bias the analysis untowardly.

A majority of articles only stated the outcomes of the mentoring umbrella without addressing mechanisms via which they exert their influence [65–69, 71, 74, 75, 86, 93, 94, 97, 100, 109, 118, 123, 130, 149, 150, 177, 182]. Given how mechanism papers formed the minority, there were concerns that non-mechanism papers would bias the data. There were also no papers describing the mechanism via which instruction influences personhood. Regardless, most of the mechanisms described were consistent with each other as well as the data derived from non-mechanism papers.

## Discussion

### Stage 6 of SEBA: synthesis of SSR in SEBA

In answering its primary question, this SSR in SEBA of the mentoring umbrella’s effects on PIF provides a number of insights into the mentoring umbrella’s influence on the stages of PIF development and the role of the host organization.

When applied longitudinally to an individualised learning relationship, across different settings involving one learner or a small group of learners with common goals, abilities and experiences, the mentoring umbrella provides an individualised perspective of development. This approach accounts for the physician-in-training’s and the instructor’s, teacher’s, coach’s, supervisor’s and tutor’s abilities, availabilities, attitudes, context, competencies, demographics, experiences, goals, motivations, and needs, in addition to building upon the physician in training’s successes, failures and reflections to enhance their longer term development. The overlapping elements within the mentoring umbrella provide synergistic support in addressing the influences of the physician-in-training’s societal, professional, clinical, academic, research, and personal considerations, the regnant sociocultural considerations, the influence of prevailing healthcare and educational system and the impact of the local hidden, informal and formal curriculum, upon PIF. This affirms the notion that the mentoring umbrella may be applied widely and in the stage based manner that allows them mentoring umbrella to shape PIF.

#### The mentoring umbrella’s influence on PIF

##### Stage One. Building a personalised cognitive base

The first stage of mentoring umbrella’s influence on PIF begins with the building of a ‘cognitive base’ of knowledge, skills, relevant expectations, roles, responsibilities around the physician-in-training’s goals, abilities, milestones, experience and setting. The cognitive base also inculcates regnant standards of professionalism and sociocultural considerations. Much of this personalisation in this stage falls upon tutoring, teaching and instruction.

Applied longitudinally, the mentoring umbrella also advance mutual understanding, trust and open communication, networking, interprofessional collaborations, research output, and enhances clinical and research competencies.

##### Stage Two. Codes of Practice (COP)s

Early exposure to clinical practice builds upon a personalised cognitive base and occurs in communities of practice. Barab et al. (2004) define CoPs as “*a persistent, sustaining social network of individuals who share and develop an overlapping knowledge base, set of beliefs, values, history and experiences focused on a common practice and/or enterprise*” [183]. Here the mentoring umbrella facilitates personalised clinical exposure, supports the application and appraisal of knowledge, skills and competencies, provides feedback and oversees remedial exercises. In remedial processes the coaching and supervision elements of the mentoring umbrella focus attention on competency gaps and boost confidence in the learner’s Individual and Societal Rings.

##### Stage Three. The socialisation process

Cruess et al. (2015) [184] describes the socialisation process as “*a representation of self, achieved in stages over time during which the characteristics, values, and norms of the medical profession are internalised, resulting in an individual thinking, acting and feeling like a physician”*.

Whilst technically part of the COP, the precise mechanism in which the socialisation process helps the integration of new values, beliefs and principles are integrated into current identities remains unclear. However it does appear that within the socialisation process the mentoring umbrella provides physicians in training with personalised, responsive, appropriate and timely support as they confront ethical, cultural, philosophical, religious and social issues that conflict with their Innate, Individual, Relational and Societal values, beliefs and principles. Here, coaching’s ability to observe [65, 67], listen deeply [65], keenly question (218), evaluate and identify gaps [68], explore solutions [65, 66, 69], provide specific and concrete feedback [67, 68], support reflection [65, 66, 69], set goals [69], develop a comprehensive study plan [69] and hold the individual accountable [68] helps focus efforts on particular areas of identity inculcation, career readiness [185], remediation of professional identities and character education [186]. Critically, coaching and supervision provide this help whilst being sensitive to the learner’s wellbeing, and goals [66]. Instruction impacts identity development in Individual and Societal aspects of personhood. These combinations of approaches would be critical to the provision of affirmation, feedback, facilitated reflection, career guidance, holistic and longitudinal support, introduction of a variety of opportunities and resources, sharing networks and “stress inoculation” important to facilitating reflection, the provision of feedback [187, 188]. Addressing dyssynchrony also highlights the role of the mentor in assessing and supporting the mentees. This continuous multipronged approach facilitates the nurturing of an enduring and personalised mentoring relationship.

#### The role of the host organization

This review also underscores the role of the host organisation [82, 171] in structuring effective mentoring relationships [170, 173, 174]. Echoing recent reviews on mentoring, the host organisation plays a critical role in the selection and matching of motivated mentees and trained and experienced mentors who share complementary goals. The host organization plays a critical role in establishing a common code of conduct, oversight [189] and assessment [190] process, as well as a supportive and nurturing environment. The host organization must also provide longitudinal ‘protected time’, support and recognition of trained mentors over the course of a mentee’s developmental journey. A further aspect in a mentor’s armamentarium must be access to user-friendly and robust communication platforms that enable timely, personal and appropriate feedback. Such a platform will also aid gathering of input on the mentee’s situation, development, goals and needs.

Here, the various aspects of the mentoring umbrella encapsulate many of the primary influences upon PIF set out by Cruess and colleagues (2015, 2018, 2019) [2, 23, 184]. Pending further studies, it may yet be possible to suggest that purposeful, structured nurturing of PIF is a mentored process.

### Limitations

One of the main limitations of this study was its inability to differentiate residents and junior doctors in training from more senior doctors such as consultants, attendings, specialists and senior consultants who have completed their training and physicians who are not in training programs. This limited the number of articles included. In addition, difficulties separating these groups also made analysis of the data difficult given the different levels of experience, roles, responsibilities and needs amongst the included groups of physicians given the diversity of the training programs and different settings and educational and healthcare programs adopted.

Moreover, whilst this study was intended to analyse the wide range of current literature on mentoring and PIF programs, our review was limited by a lack of consistent reporting of current programs. Furthermore, most of the included papers were largely drawn from North American and European practices potentially limiting the applicability of these findings in other healthcare settings. This was compounded by our focus upon articles that were published in English.

Whilst taking into account the limited resources and availability of the research and experts teams and limiting the review to the specified dates to increase the chances of completing the review, this too could have seen important articles excluded.

## Conclusion

This SSR in SEBA highlights the role of mentoring umbrella in nurturing PIF. Whilst the three stages built on posits by Cruess and colleagues remain to be evidenced, it does underline the need for longitudinal and holistic evaluation of the mentoring umbrella’s impact on PIF. Further understanding of the mentoring umbrella and its role in PIF also demands better appreciation of the need for personalised, holistic and longitudinal assessments and individualised and timely support. These gaps represent some of the key areas for future studies seemingly as the role of portfolios and longitudinal assessment measures to enhance support of evolving concepts of PIF develop.

## Supplementary Information

**Additional file 1:** **Appendix A.** Tabulated Summaries

## Data Availability

All data generated or analysed during this review are included in this published article and its supplementary files.

## Abbreviations

CoP: Community of Practice
PICOs: Population, Intervention, Comparison, Outcome and Study Design
PIF: Professional Identity Formation
SSR: Systematic Scoping Review
SEBA: Systematic Evidence-Based Approach
RToP: Ring Theory of Personhood
PRISMA: Preferred Reporting Items for Systematic Reviews and Meta-Analyses
YLLSoM: Yong Loo Lin School of Medicine
NCCS: National Cancer Centre Singapore
