# Supplementary material for: The role of mentoring, supervision, coaching, teaching and instruction on professional identity formation: a systematic scoping review
Source: BMC Med Educ. 2022 Jul 8;22:531. doi: 10.1186/s12909-022-03589-z (PMC9270794; doi:10.1186/s12909-022-03589-z)
Supplement: Supplementary file 1 — Additional file 1: Appendix A. Tabulated Summaries [file 12909_2022_3589_MOESM1_ESM.pdf]

Appendix A. Tabulated Summaries (207 Articles)

Author, Year	Title	Background and Aims	Theoretical Approach and Data Collection Methods							Findings	
			Study Population	Location of Study	Mentoring structure	Primary/ Secondary/ Tertiary	Qualitative/ Quantitative/ Mixed Methods	Study Design	Outcome Measures and Tools	Main Empirical Findings	Insights Drawn
Ackroydand and Adamson, 2015	Mentoring for new consultants	There is increasing evidence of the benefits of having a mentor during the early years as a consultant. Mentoring encourages and provides support to an individual in their professional development. Although there are different forms of mentoring there is recognition that developing a formal mentoring scheme can provide a consistent approach and support within a framework.	Consultants	United Kingdom	Not indicated	Tertiary	Qualitative	Opinion paper	Clinical outcomes	There is always the concern that with continuing difficulties in trying to balance clinical commitments with the declining time for supporting professional activities that mentoring is viewed as a luxury and not a necessity. However, developing doctors who are more empowered and satisfied in their job is a reason to ensure mentoring gets its recognition. Mentoring can help new consultants to face the challenges of providing healthcare in a changing environment with more confidence and resilience. Mentoring provides the tools needed to equip mentees to lay the foundations they need to build their future. This can benefit the organisation they work in and ultimately improve their patient care while also encouraging them	There is potential that the process of mentoring can improve an individual's development, and motivate and encourage them to develop the skills needed to achieve their goals, thus having an impact on ultimately improving their ability to deliver an effective patient-centred service.

										to become the next generation of mentors.	
Agzarian et al. 2019	Moving to the other side of the table — transitioning from residency to faculty and the value of mentorship	Not indicated	Residents	Canada	Mixed methods	Tertiary	Not indicated	Editorial	Not indicated	Not indicated	The author offers the following as roles of a good mentor:<br>verify thinking, inspire imagination, multiply clinical experience, provide unvarnished criticism and advice, serve as a congenial colleague, and be understanding.<br><br>Firstly, mentors serve a wide range of functions, and as such serve a dynamic role in the lives of their mentees, and are accordingly crucial to the development of new surgeons (particularly in the academic realm). Secondly, we all need mentors.
Altieri et al. 2020	What Are Women Being Advised by Mentors When Applying to Surgery?	Despite an increase in percentage of women entering the surgical field, women tend to favour certain surgical subspecialties. The purpose of this study was to investigate how surgeons advise trainees in pursuit of a surgical career.	Residents	America, American College of Surgeons	Dyadic Informal program	Primary	Quantitative	Cross-sectional survey	Not indicated	There were 663 respondents, of which the majority (n 5 465, 70.99%) were male. When asked if participants had a role model in medical school, 61.10 per cent had male role models/mentors, whereas only 7.96 per cent had female role models/mentors. Among the 23 surgical subspecialties listed, the top five specialties viewed as receptive for women were breast surgery, obstetrics and gynaecology, plastic surgery, ophthalmology, and GS. Surgical	There is inherent gender-based bias in advisement of trainees that may affect surgical specialty choice. Surgeon gender, age, and surgical specialty could be predictors as to how trainees are advised.

										specialty and gender of the respondent played a role in how surgeons advised men and women trainees, especially in specialties that traditionally have less female representation.	
Amonoo et al. 2017	Residents' Experiences with Mentorship in Academic Medicine	Although mentorship is essential for the professional development of physicians, the literature on trainees' mentorship experiences and perceptions of effective mentoring is more limited. This descriptive study examines residents' experiences of mentoring and their perceptions about the impact of mentorship on professional development, comparing experiences in mentoring that is assigned versus self-initiated.	Residents	United States of America	Dyadic Informal program	Primary	Quantitative	Cross-sectional observational study	Research output, career advice, networking and work-life balance.	Of the 327 eligible senior residents, 204 (62%) responded and completed the survey. Most residents (82%) reported multiple mentors and 65% reported that their primary mentorship relationship was self-initiated. Residents who self-initiated their primary mentorship were significantly more likely to strongly/somewhat agree that their mentor had a positive impact on publications and scholarly projects (88 vs 44%, p = 0.0063) as well as research (88 vs 55%, p = 0.0001) compared to residents with assigned mentorship, with no significant differences measured by gender, race, or ethnicity. Forty-four percent of residents indicated they had unmet needs for mentoring in at least one of several professional areas.	Most residents perceive mentoring relationships as important to many aspects of their career development. Still 44% of residents reported unmet needs for mentoring in one or more areas, a result that needs further exploration. Since the majority of residents' primary mentoring relationships were self-initiated rather than assigned, and these were seen as more important for research and publications, programs should consider how to support the connection between residents and potential mentors.

Areephanthu et al. 2015	Impact of professional student mentored research fellowship on medical education and academic medicine career path.	This study explores the long-term impact of the Professional Student Mentored Research Fellowship (PSMRF) program at the University of Kentucky College of Medicine (UKCOM) on medical students' research productivity and career paths.	PSMRF graduates and UKCOM students	University of Kentucky College of Medicine (UKCOM), United States of America	Network formal programm e	Primary	Quantitative	Cohort Study	Demographic characteristics, academic profiles, number of publications and residency placements from 2007 to 2012.	PSMRF students had higher MCAT scores at admission (31.5 ± 0.6 vs. 30.6 ± 0.2, p = 0.007) and achieved higher USMLE Step 1 scores (228 ± 4.2 vs. 223 ± 1.5, p = 0.03) than comparison group. PSMRF students were more likely to publish PubMed-indexed papers (36.7% vs. 17.9%, p < 0.0001), achieve AOA status (19.3% vs. 8.5%, p = 0.0002) and match to top 25 US News and World Report residency programs (23.4% vs. 12.1%, p = 0.008). A greater proportion of PSMRF fellows matched to top tier competitive specialties (23% vs. 14.2%, p = 0.07), however this difference was not statistically significant.	The PSMRF program shows a significant increase in enrollment, as well as positive associations with indicators of success in medical school and subsequent quality of residency program.
Arnold et al. 2005	Mentoring the next generation: a critical task for palliative medicine.	Not indicated	Physicians	United States of America	Not indicated	Tertiary	Qualitative	Commentary	Not indicated	While we are building mechanisms to mentor junior academicians, however, we must not neglect mentoring junior clinicians. We need to think about the mentoring it requires to develop a successful hospice medical director. How can we make sure that senior clinicians are given the skills and time needed to	Not indicated

										mentor their junior colleagues. The clinical models are different from those in large medical centres and we need to make sure the mentoring processes will fit the environment. Moreover, we need to develop distance-mentoring for those clinicians who are working in jobs where there are no senior palliative care clinicians.	
Back et al. 2007	Efficacy of communication skills training for giving bad news and discussing transitions to palliative care	Few studies have assessed the efficacy of communication skills training for postgraduate physician trainees at the level of behaviors. We designed a residential communication skills workshop (Oncotalk) for medical oncology fellows. The intervention design built on existing successful models by teaching specific communication tasks linked to the patient's trajectory of illness. This study	Residents	United States of America	Network Formal programm e	Primary	Quantitative	Pre-post observation study	Communication skills	Compared with preworkshop standardized patient encounters, postworkshop encounters showed that participants acquired a mean of 5.4 bad news skills (P<.001) and a mean of 4.4 transitions skills (P<.001). Most changes in individual skills were substantial; for example, in the bad news encounter, 16% of participants used the word "cancer" when giving bad news before the workshop, and 54% used it after the workshop (P<.001). Also in the bad news encounter, blinded coders were able to identify whether a standardized patient encounter occurred before or after the	Oncotalk represents a successful teaching model for improving communication skills for postgraduate medical trainees.

		evaluated the efficacy of Oncotalk in changing observable communication behaviors.								workshop in 91% of the audio recordings.	
Balmer DF, Giardino AP, Richards BF., 2012	The dance between attending physicians and senior residents as teachers and supervisors.	To examine how attending physicians and senior residents negotiated shared responsibilities for teaching and supervising on clinical work rounds.	Senior residents	United States of America	Network Formal programm e	Primary	Qualitative	Focus group	Themes surrounding the dynamic relationship between attending physicians and senior residents.	Like a traditional dance with a priori choreography, and consistent with the traditional premise in graduate medical education, attending physicians frequently "stood back" and senior residents, accordingly, "stepped up" and took on teaching and supervising responsibilities. Less often, both attending physicians and senior residents assumed the lead, or attending physicians stepped up rather than entrust senior residents. The complex clinical context sometimes changed the choreography. Attending physicians and senior residents understood their mutual responsibilities but were not bound by them; they improvised to maintain high-quality patient care.	The metaphor of a dance enabled us to better understand not only how attending physicians and senior residents negotiate shared responsibilities for teaching and supervision on clinical work rounds, but also how the clinical context impacts this negotiation. A better understanding of this negotiated relationship may help to clarify assumptions and set realistic expectations for what it might take for senior residents to assume progressive responsibility for these responsibilities in today's clinical context.
Barham et al. 2019	The role of professional supervision for palliative care doctors in New Zealand: a quantitative	Professional supervision, a collaborative relationship that promotes reflection on contextualised	Junior doctors	New Zealand	Dyadic Informal program	Primary	Quantitative	Cross-sectional survey	Survey results	Overall, professional supervision was felt to be important, particularly by those currently participating in professional supervision, for enhancing clinical	Despite identified barriers, New Zealand-based palliative care doctors reported professional supervision to be important and beneficial both personally and

	survey of attitudes and experiences	work-related issues for the benefit of ongoing learning and development and improved professional practice, is not well understood in the medical context. This study aimed to explore the attitudes and experiences of professional supervision among doctors working in palliative care in New Zealand.								functioning and supporting doctors' wellbeing. Barriers to undertaking professional supervision included finding a supervisor, lack of funding and time, with the most significant factor being whether the workplace supported professional supervision. Supervisees' responses showed professional supervision to be a safe experience, addressing a wide range of issues, with a positive effect on personal coping.	professionally. Further consideration should include the impact of professional and organisational culture on uptake of professional supervision, future research and acknowledgment that palliative medicine may be in a position to highlight the benefits of professional supervision for the wider medical community.
Bartlett et al. 2019	Development of a “Surgical Shadowing Scheme” to improve undergraduate experiences of surgery	To establish a sustainable model for a “Surgical Shadowing Scheme” and assess how this affects undergraduate attitudes to surgical careers.	Medical students	United Kingdom, UCL Medical School and UCL Partners	Dyadic Formal program	Primary	Quantitative	Cohort Study	Participation rate and satisfaction with scheme	After running for four consecutive years, approximately 220 students have participated in the scheme across a range of surgical units and specialties. A total of 91.5% of the students were pre-clinical (years 1–3), whilst the remainder were clinical (years 4–6). Fifty-four percent were female and 46% male. Eighty-three percent of the students did not have any previous experience of the specialty that they shadowed, and 67% agreed that participating in the scheme had either “increased” or “strongly	This is the first published example of an established “Surgical Shadowing Scheme” for medical undergraduates. Our Surgical Shadowing Scheme has been highly valued by students and indicates that even a single high-quality surgical exposure is sufficient to increase the desire of undergraduates to pursue a surgical career. We hope that this Surgical Shadowing Scheme will act as a blueprint for other centres to develop their own shadowing schemes, in turn helping to ensure that surgery continues to inspire and attract the very best candidates for the future.

										increased" their desire to pursue a surgical career. Ninety-four percent said they would "recommend" or "strongly recommend" the Surgical Shadowing Scheme to a peer. Over a third of students reported scrubbing-up during their placements and 35% of these directly assisted the lead surgeon. Traditionally male-dominated surgical sub-specialties recruited a high proportion of female students.	
Beanlands et al. 2020	An LGBTQ+ mentorship program enriched the experience of medical students and physician mentors	Recognizing the critical importance of mentorship for minority students, we developed a novel mentorship program for LGBTQ+ medical students at the Schulich School of Medicine and Dentistry (Schulich). To understand how mentors and mentees were impacted by Schulich's LGBTQ+ mentorship program, we distributed online surveys to participants from the 2017-2018	Medical students	Schulich School of Medicine and Dentistry, Canada	Group Formal program	Primary	Qualitative	Pre-post study	Benefits of the program.	Not indicated	Students felt that role-modelling by LGBTQ+ physicians provided reassurance about their capacity to achieve professional success, and they experienced a sense of belonging amongst peers and faculty. Mentors identified the value of modelling success for gender and sexual minority students and gained new insight into the educational experience of minority learners.

		and/or 2018-2019 academic years and performed a qualitative analysis.									
Benson et al. 2002	Effective faculty perceptoring and mentoring during reorganization of an academic medical center	“The experience and lessons learned in the design, implementation and initial evaluation of a demonstration faculty-to-faculty mentoring program, during a time of major institutional reorganization, are described. The question addressed was: Can a voluntary mentoring program be established with minimal resources and be effective in the context of major organizational change? Key design elements included two-tiered programs (one year preceptoring and multi-year mentoring), voluntary participation, and selection of senior faculty members by the	Faculty members	MCP Hahnemann University National Center of Leadership in Academic Medicine, United States of America	Formal mentoring program	Primary	Mixed methods	Interventional study	Self-reported survey data (satisfaction), focus groups, individual interviews, database of participation statistics	A total of 20% of junior faculty and 30% of senior faculty participated. Faculty indicated the program was worth the time invested, had a positive impact on their professional life and increased productivity. There was high satisfaction with the mentoring relationship, especially the psychosocial mentoring functions, and a trend toward increased retention of minority faculty. Within two years, the program was institutionalized into the Office for Faculty Affairs, and faculty approved a mentoring policy.	It is concluded that voluntary mentoring programs can have a positive impact on junior and senior faculty satisfaction, reinvigorate the collegial culture, and improve productivity and retention even during a time of reorganization and minimal resources.

		junior faculty members"									
Berk et al. 2005	Measuring the effectiveness of faculty mentoring relationships	Mentor" is a term widely used in aca- demic medicine but for which there is no consensus on an operational definition. Further, criteria are rarely reported for evaluating the effectiveness of mentor- ing. This article presents the work of an Ad Hoc Faculty Mentoring Committee whose tasks were to define "mentor- ship," specify concrete characteristics and responsibilities of mentors that are measurable, and develop new tools to evaluate the effectiveness of the mentoring relationship.	Faculty members, medical students	Johns Hopkins University School of Nursing, United States of America	Informal and formal mentoring programs	Primary	Quantitative	Cross-sectional survey	Mentorship Profile Questionnaire, Mentorship Effectiveness scale	The committee developed two tools: the Mentorship Profile Questionnaire, which describes the characteristics and outcome measures of the mentoring relationship from the perspective of the mentee, and the Mentorship Effectiveness Scale, a 12-item six-point agree–disagree-format Likert-type rating scale, which evaluates 12 behavioral characteristics of the mentor. These instruments are explained and copies are provided. Psychometric issues, including the importance of content-related validity evidence, response bias due to acquiescence and halo effects, and limitations on collecting reliability evidence, are examined in the context of the mentor–mentee relationship.	The research and experience on faculty mentoring relationships in academia, and medical schools in particular, over the past 25 years have produced lists of definitions, functions, and programs, but miniscule evidence of effectiveness. The concept of mentoring remains unclear and imprecise and instruments designed to evaluate mentoring programs rarely do. The effectiveness of formal and informal medical faculty mentoring programs intended to promote the professional growth of junior faculty and the academic success of students is based more on assumption than on demonstrated empirical evidence.
Bhatnaga et al. 2020	The Need for More Mentorship in Medical School	Mentorship, a supportive relationship that actively provides knowledge and insight, has many benefits. Although not extensively	Medical students	United States of America	Not indicated	Primary	Mixed methods	Cross-sectional survey	Quality of mentorship, impact on career decisions, research and career trajectory.	Sixty-one students responded to this survey. Fifty-nine percent of respondents said they did not receive enough mentorship in medical school while 63.9% of respondents said their quality of mentorship	Our study demonstrates the profound impact mentorship has on a medical student's career. Respondents believed their mentorship experiences strongly impacted their decisions involving rotation choices, residency programs, field of

		studied, medical students pursuing various specialties have diverse experiences with mentorship. To understand how mentorship impacts medical student decisions involving rotation choices, residency programs, field of practice, interest in research, and career trajectory.								was good/very good. Most survey respondents strongly agreed/agreed that the amount and quality of mentorship impacted their decisions involving rotation choices, residency programs, field of practice, and career trajectory. Qualitative data analysis led to the emergence of three themes: students pursuing primary care had positive mentorship experiences as compared to students pursuing non-primary care careers, female students stated they did not receive enough mentorship, and a majority of students cited the lack of formal mentorship as an area of improvement.	practice, and career trajectory. Participants also believed availability in the amount and quality of mentorship could be improved. The perceived lack in the amount and quality of mentorship may have negative implications on medical students' career prospects.
Birch et al. 2007	The impact of a formal mentoring program for minimally invasive surgery on surgeon practice and patient outcomes.	Surgeons need a process by which to safely introduce new procedures, such as minimally invasive surgery (MIS), into practice. Emerging evidence would suggest that an effective strategy is the implementation of a mentorship	Surgeons	University of Alberta, Canada	Formal program	Primary	Quantitative	Retrospective review	Clinical outcome, patient safety	A total of 7 general surgeons were mentored at 1 site. After 1 year of intense mentoring, the number of surgeons completing >12 cases/y increased from 2 to 4, and the number of advanced MIS cases completed (excluding mentored ones) increased from 35 to 102. Fifty-three cases (52% of total) were formally mentored. Total conversions to open	Surgeons and the institutions in which they work have a duty to adopt advanced MIS techniques in a safe and appropriate manner. This study demonstrates that a mentorship program is an effective strategy for safely introducing advanced MIS into practice.

		program. This study analyzed the effect of mentoring on a single institution's advanced MIS practice.								surgery decreased from 14.3% to 6.4% (P = .12). The number of colorectal resections increased from 11 to 92 (P = .0027). Intraoperative complications were not significantly decreased, eg, from 17.1% to 7.1% (P = .06). Postoperative complications remained unchanged (15.0% to 16.5%).	
Brook et al. 2020	The Influence of Mentors in Orthopedic Surgery	The importance of mentorship in medicine has been established. However, little is known regarding the influence of mentors in orthopaedic surgery. This study sought to determine the prevalence of mentoring relationships in orthopaedic surgery, assess the influence of mentors in specialty and subspecialty selection, and evaluate the importance of gender in orthopaedic mentoring relationships.	Medical students and residents	Not indicated	Network Formal program	Primary	Quantitative	Cross-sectional survey	Survey results	A total of 117 (95 males and 22 females) surveys were returned. The majority of respondents (66.7%, n=78) had at least one mentor in their career, and the majority of respondents (66.7%, n=52) were satisfied with their mentoring experience. Residency was the most common time to have a mentor, and 73.3% (n=44) of respondents indicated their mentor was influential in determining their subspecialty. Although only 50% of respondents indicated they had a mentor in medical school, 84.2% (n=32) believed their mentor was influential in selecting an orthopaedic surgery residency. The majority (79.4%, n=62) of respondents did not have a preference on the gender of their mentor.	Many orthopaedic surgeons have a mentor at some point in their career who influenced their specialty or subspecialty decision. Mentoring experiences are less prevalent in medical school, and female medical students may lack accessibility to mentoring opportunities. Future efforts should focus on opportunities that connect medical students to orthopaedic surgery faculty to further diversify the field and close the gender gap.

Brown et al. 2007	Seeking informed consent to cancer clinical trials; evaluating the efficacy of doctor communication skills training	Clinical trials have come to be regarded as the gold standard for treatment evaluation. However, many doctors and their patients experience difficulties when discussing trial participation, leading to poor accrual to trials and questionable quality of informed consent. We have previously developed a communication skills training program based on a typology for ethical communication about Phase II and III clinical trials within four categories. The training program consisted of a 1 day experiential workshop that included didactic teaching, exemplary video and role play. The aim of this study was to evaluate the effectiveness of the	Oncologists	Australia	Group, interactive Formal program	Primary	Quantitative	Cross-sectional study	Doctor-patient communication.<br><br>The Decisional Conflict Scale, the Satisfaction with Decision Scale, the Ellis Clinical Trails Knowledge Scale, the Ellis Attitudes to Clinical Trials Scale, the Speiberger Strait-Trait Anxiety Scale (Stait version), the Information Preference Scale and Control Preference scale.	Doctors increased their use of some aspects of shared decision-making behavior ($t87 = 1.945$, $p = 0.05$) and described some aspects of essential ethical/clinical information more commonly. In addition they used less coercive behaviors ($z = 1.976$, $p = 0.048$). However, they did not provide more clinical information or structure their consultations in the recommended fashion. Patients in the post-training cohort reported more positive attitudes to clinical trials, but other outcomes were not affected by the intervention.	This short training programme demonstrated limited success in improving the oncologist's communication skills when gaining informed consent. A larger randomized controlled trial of extended training is now underway.

		communication skills training workshop.									
Brown et al. 2020	Becoming a clinician: Trainee identity formation within the GP supervisory relationship	Becoming a clinician is a trajectory of identity formation in the context of supervised practice. This is a social process where the supervisory relationship is key. Therefore, to know how to support identity formation of clinical trainees, it is necessary to understand how this happens within the supervisory relationship. Our aim was to develop a conceptualisation of trainee identity formation within the general practice supervisory relationship to aid its support.	Residents	Ireland	Dyadic Formal program	Primary	Qualitative	Cohort study	Impact of the program.	We identified three social Discourses centring on: clinical responsibility, ownership of clinical knowledge, and, measures of trainee competency. Versions of these Discourses defined four trainee/supervisor relational arrangements within which trainee and supervisor assumed reciprocal identities. We labelled these junior learner/expert clinician, apprentice assistant/master coach, co-clinicians, and lead clinician/advisor. We found a trajectory across these identity arrangements. Behind this trajectory was an invitation by the supervisor to the trainee into the social space of clinician and a readiness of the trainee to accept this invitation. Congruence in supervisor and trainee positioning was important.	In the supervisory relationship, trainee and supervisor adopted reciprocal identities. For trainees to progress to identity of lead clinician, supervisors needed to invite their trainee into this space and vacate it themselves. Congruence between supervisor positioning of their trainee and trainee authorship of themselves was important and was aided by explicit dialogue and common purpose. We offer a model and language for trainees, supervisors and faculty to facilitate this.
Butow et al. 2008	Increasing oncologists' skills in eliciting and responding to emotional cues:	Psychological morbidity in cancer patients is common, but	Oncologists	Australia	1. Group, interactive 2. Formal	Primary	Quantitative	Cross-sectional study	Doctor stress and burnout.	Doctors in the intervention group displayed more creating environment and fewer	This short training programme demonstrated a positive effect on aspects of doctor behaviour. Video-conferencing after a short

	evaluation of a communication skills training program	often undetected and untreated. We developed a communication skills training (CST) program targeting this issue, and evaluated its impact on doctor behaviour.							Maslach Burnout Inventory	blocking behaviours at both follow-ups; however, these differences did not reach statistical significance. Intervention doctors valued the training highly, but did not report substantial reductions in stress and burnout.	training course may be an effective strategy for delivering CST.
C. A. Taylor et al. 2009	The influence of mentorship and role modeling on developing physician-leaders: views of aspiring and established physician-leaders	Although the benefits of mentoring in academic medical centers have been amply discussed, the major focus has been on conferring traditional academic skills (e.g., grantsmanship, publications, etc.). In contrast, little attention has been given to the career development of physician-leaders (e.g., communication, vision, team-building, etc.). OBJECTIVES: To understand the role and functions of mentoring and role-modeling in developing physician-leaders	Clinic faculty (physician leaders and aspiring leaders)	United States of America	Not indicated	Primary	Qualitative	Interview	Professional development	Twenty-five Cleveland Clinic faculty participated (14 established physician-leaders, 11 aspiring leaders). Three themes emerged: 1. Role modeling was differentiated as a valued experience separate from mentoring, with respondents describing the significant influence of purely observational learning and "watching leaders-in-action". 2. Many respondents favored a series of "strategic" interactions with various individuals about specific professional issues rather than traditional, longitudinal mentoring experiences. 3. Emotional and psychological support was considered the most valued type of interventional activity.	In our small sample both established and aspiring physician leaders believed that mentorship and role modeling played a significant role in their career development. Short, focused "strategic" mentoring relationships were favored by many over the classic longitudinal experience. Our participants valued role-modeling as an experience separate from mentoring and described the impact of learning from direct observation of skilled leaders. The educational implications of these findings are summarized.

		as experienced by aspiring and established physician-leaders. DESIGN: Qualitative design using a stratified purposeful sample and inductive analysis.									
Thiedke et al. 2004	Student observations and ratings of preceptor's interactions with patients: the hidden curriculum	Professional values and behavior are conveyed to students through both formal and informal curricular activities. This study examined 1st-year students' observations of community-based physicians' behaviors during a community-based clinical experience.	Medical students	United States of America	Not indicated	Primary	Quantitative	Cross-sectional	Clinical behaviours	Descriptive statistics were computed. 119 students completed the instrument (87% response rate). Students rated aspects of physicians' demeanor with patients highest (mean ranges 4.7-4.57). The lowest rated item was the physician's view of his or her professional role (M = 3.39), eliciting patients' ideas about illness and treatment (M = 3.55), and modeling interviewing techniques learned in class (M = 3.71).	Community-based physicians reinforce many professional values associated with positive role-modeling aspects of the physician-patient interaction.
Carey and Weissman, 2010	Understanding and finding mentorship: a review for junior faculty.	Mentorship is considered central to physician success, and yet relatively few physicians report having formal mentors. Ever-increasing demands on physician time as well as multiple personal and	Junior faculty	USA	Not indicated	Secondary	Mixed methods	Review	Mentoring self-evaluation tool	In this article, we describe the attributes of the "ideal" mentor and the roles mentors commonly play in a protégé's career. We then discuss a framework for optimizing one's chance of fostering mentoring relationships. We conclude by discussing the evolution of and transitions in mentoring relationships,	Ever-increasing demands on physician time, geographic isolation from more senior faculty in the field, and multiple conflicting responsibilities make finding alternatives to the dyadic mentoring relationship essential. Having a constellation of mentors at different levels and with different skills is a viable and user-friendly alternative to seeking that one ‘‘academic

		professional responsibilities, make it challenging to find and sustain mentoring relationships. These challenges may be even greater in palliative medicine, a field with few mid-level to senior faculty and in which the supply of physicians is inadequate to meet the anticipated demand.								as well as how one might transition from protégé to mentor.	parent'' described in traditional mentoring. One's attitude and approach is essential to the success of any mentoring relationship, whether with peers or more senior physicians. In medicine, intellectual capital is a crucial asset. Developing and sustaining mentoring relationships benefits not only a protégé´s career but also to the growth of our field.
Carma L. Bylund et al. 2009	The implementation and assessment of a comprehensive communication skills training curriculum for oncologists	The objective of this paper is to report the implementation and assessment of the Comskil Training Curriculum at Memorial Sloan-Kettering Cancer Center.	Oncologists	United States of America	Didactic and Role-Playing Formal	Primary	Quantitative	Cohort study	Use of communication skills.<br><br>Medical Interaction Process System, Cancer Research Campaign Workshop Evaluation Manual	Communication skills related to two of the six major skill sets, Establishing the Consultation Framework and Checking, increased following training. Limited changes emerged in three skill sets, while one skill set, Shared Decision Making, did not change. Doctors who attended more training modules had higher levels of change. Female participants demonstrated three skills more frequently than males post-training.	The intervention produced significant communication skills uptake in a group of experienced attending clinicians, mediated by the amount of training. Future research should focus on the dose of training necessary to achieve skills uptake and the effect of skills training on patient outcomes.
Celenza and Rogers, 2006	Qualitative evaluation of a formal bedside clinical teaching	Bedside clinical teaching in emergency departments is	Emergency medicine registrars	Australia	Bedside teaching Formal	Primary	Qualitative	Pre-post study survey	Session evaluating questionnaires (items: describe	Learners ranked history taking and physical examination technique as the most frequently	Formal bedside teaching is effective if organised with adequate staffing to quarantine the teacher and

	programme in an emergency department	usually opportunist or ad hoc. A structured bedside clinical teaching programme was implemented, where a consultant and registrar were formally allocated to teaching and learning roles separated from the usual departmental management or clinical roles. Themes emphasised included clinical reasoning, practical clinical knowledge, communication, physical examination, procedural and professional skills.<br>Aim: To evaluate the perceived educational value, effects on patient care and areas for ongoing development.							the teaching methods, learning experience and outcomes, problems encountered and suggestions for change.)	learnt item, but clinical reasoning as the most important theme learnt. Informal discussion and performance critique or constructive feedback were the most frequent teaching methods. The biggest obstacle to learning was learner apprehension. The most frequent positive effect on patient care was faster management, decision making or disposition. Most often, no negative effect on patient care was identified.	learner from routine clinical duties, and concentrating on themes best taught in the patient setting. Clinical reasoning and clinical knowledge were perceived to be most important, with positive effects on patient care through more thorough assessment and faster decision making.
Chapman et al. 2021	Development of teaching, mentoring and supervision	Teaching, mentoring and supervision are fundamental skills with a specific commitment	Residents	New Zealand	Apprentice ship in clinical setting Formal	Primary	Qualitative	Focus group	Experience of participants.	121 registrars from 16 District Health Boards responded. Registrars supervise two juniors daily. Fewer than 1:4 have formal training in	Registrars are expected to teach, mentor and supervise junior colleagues but experience a 'frustrated apprenticeship': formal training is minimal and

	skills for basic training registrars – a frustrated apprenticeship?	within the Royal Australasian College of Physicians professional practice framework. The new basic training standards include 'use of appropriate educational techniques to facilitate the learning of peers, junior colleagues and other health professionals and to provide supervision for junior colleagues' but it is unclear how basic physician trainees and equivalent grade doctors will provide, learn or develop TMS skills. The aim is to explore how registrars provide, learn and develop teaching, mentoring and supervision skills.								teaching, mentoring and supervision skills.<br>Free text and focus group themes include: informal development by observing role models plus personal experience of giving and receiving teaching, mentoring and supervision, inequitable access to development opportunities and formal training, barriers include workload and unsupportive learning cultures. Some registrars lack confidence in delivering teaching, mentoring and supervision.	informal training is dependent on variable role models, opportunities and systematic support. Registrars feel unprepared and lack confidence despite wanting to succeed in this domain. Suggestions for improvement include baseline formal training, purposeful role modelling by seniors, and equitable promotion of teaching, mentoring and supervision opportunities.
Cheng et al. 2020	Academic Coaching Of Medical Students During The	The authors observed that students requiring academic support often had	Medical students	Singapore	Regular 30 minutes scheduled meetings, formal	Primary	Quantitative	Cohort study	Students' overall	Importantly, noticeable improvements in students' overall academic performance and increasing numbers	Regular and short-interval engagement with students during social isolation circumstances allows them to feel safe in reaching out

	Covid-19 Pandemic	ineffective learning strategies, poor motivation and suboptimal communication skills, all of which were magnified by the imposition of home-based learning. A virtual academic coaching programme was thus established to engage these students and to address specific challenges that arose from an adapted education programme.			teaching program				academic performance	of students proactively utilising programme resources to optimise their individual learning journeys was observed. Further studies on academic impact and adaptive behaviour are needed as part of programme evaluation.	for help. This model of academic coaching informed by theory supports students and empowers them with the skills necessary for effective learning, adapting and thriving in a health care environment challenged by uncertainty and ambiguity.
Coates et al. 2008	Medical school curricular reform: Fourth-year colleges improve access to career mentoring and overall satisfaction.	The authors described the impact that an innovative fourth-year curriculum, the “College Program” at the David Geffen School of Medicine at the University of California–Los Angeles, which focuses on mentoring and required curricular components, has had on student perceptions of access to career	Medical students	David Geffen School of Medicine at the University of California–Los Angeles (UCLA), United States of America	Formal program	Primary	Quantitative	Cross-sectional survey	Accessibility to mentors, student satisfaction towards the curriculum, relationships with faculty members, preparedness for residency	Students in the intervention group reported a higher degree of satisfaction with accessibility to mentors and the impact they had on their educational experiences and careers than the preintervention cohort. Despite initial concerns that student freedom was going to be compromised, the students who participated in the College curriculum reported increased satisfaction with an intense foundations course, longitudinal experiences in the	In the future, it would be interesting to look at the mentors’ view of the implementation of the College system. Improved training of mentors as well as funding them for their time in the form of “buy-out” or compensation would add value to our program. Individual colleges have evolved to offer unique programs to prepare the students whose needs match their college’s mission. For example, the Applied Anatomy College serves the students who participate in the early match. Thus, their career-building component is accelerated to the late

		mentors and overall satisfaction with the fourth-year experience.								clinical setting, and scholarly projects during their senior year. Fourth-year students in the College Program were more likely to identify and develop better relationships with faculty mentors than their preintervention counterparts. They indicated excellent residency preparedness, and their overall impression of the fourth year was favorable.	summer. The Primary Care College caters to students whose careers will involve longitudinal experiences, and many of these are built in as options for their students. The Medical Specialties College has a focus on research, and students present substantial projects as part of their monthly evening seminar series. The Acute Care College provides opportunities for practicing rapid evaluation using simulation and with an association with the local emergency medical services agency.
Cochran et al. 2017	Characteristics of Effective Mentorship for Academic Surgeons	Prior qualitative work describes characteristics of successful mentoring relationships. However, no model exists of effective mentorship that is specific to academic surgery. The authors sought to describe characteristics of effective mentoring relationships in academic surgery based upon lived experiences of mid-career and senior female	Doctors	United States of America	Not indicated	Primary	Qualitative	Cross-sectional interview	Not indicated	Interviewees described the need for multiple mentors over time with each mentor addressing a unique domain. Interviewees suggested that mentees should seek mentors who will serve as strategic advisors, who will be unselfish, and who engage with diverse mentees.	This study identified a need for multiple mentors across time and disciplines, and identified 3 key characteristics of effective mentoring relationships in academic surgery. Future work in this area should generate an operational definition of mentorship that supports quantitative evaluation of mentor and mentoring panel performance.

		academic surgeons.									
Cowan and Flint, 2012	The importance of mentoring for junior doctors	Fiona Cowan and Sara h Flint report on the Kent, Surrey and Sussex Deanery's obstetrics and gynaecology mentorship scheme	Junior doctors	Not indicated	Not indicated	Tertiary	Not indicated	Commentary	Not indicated	Not indicated	The results of our surveys of the Kent, Surrey and Sussex OandG mentorship scheme show that junior specialty trainees and their senior colleagues believe mentoring is an essential part of their training, providing further encouragement and confidential emotional and academic support. The scheme has provided additional support to trainees in a number of areas, including examination preparation; work based assessments; audits; publications; and career, patient, and staff related concerns.
Croke et al. 2018	Mentorship Needs for Radiation Oncology Residents: Implications for Programme Design	Mentorship during residency guides and supports professional and personal development. Despite this, mentorship programmes within many post-graduate medicine programmes, including radiation oncology, are not standard. The objective of this qualitative study	Residents	Canada	Not indicated	Primary	Qualitative	Cross-sectional interview	Not indicated	Twenty interviews (10 residents and 10 faculty) took place between October and December 2017, at which point thematic saturation was achieved. Four major themes emerged - the perceived experiences of residents and faculty with mentorship, the evolution of mentorship needs during residency training, the mechanisms of creating mentorship relationships and peer mentorship.	In this study, the perceptions of mentorship from the perspective of radiation oncology residents and faculty were explored. Important areas of alignment and discordance were discovered. These insights will inform the development and implementation of a mentorship programme that can be adapted for use by other oncology training programmes.

		was to carry out a needs assessment to determine the perceived mentorship needs and experiences of radiation oncology residents and faculty.									
Dabbagh et al. 2019	Improving the Training Process of Anesthesiology Residents Through the Mentorship-Based Approach	Although mentoring has been accepted as an effective and nourishing component in medical learning, known mentoring programs for the residents are lacking in most countries.<br>To evaluate the mentoring program for anaesthesiology residents at Shahid Beheshti University, an examination was designed to explore the styles of mentoring programs, as well as the aims and outcome followed by such programs.	Residents	Anaesthesiology Department of Shahid Beheshti University of Medical Sciences, Iran	Not indicated	Primary	Quantitative	Cohort study	Iranian Board of Anaesthesiology In-Training Examination Scores	In the academic stage from 2014 - 2015, the overall first-time license rate for the Iranian Board of Anaesthesiology written exam was 7 of 14 (50%) and in the period of 2016 - 2017, the general rate was 11 of 14 (78%) and in the period of 2017 - 2018, it was 12 of 14 (85%). For each extra year experiencing the program, the odds ratio for passing Iranian Board of Anaesthesiology written exam was 1.7 ($P < 0.05$). The median Iranian Board of Anaesthesiology In-Training Examination percentile was considerably greater in period 2 than period 1 ($P < 0.05$). Also, there was a significant correlation between Iranian Board of Anaesthesiology In-Training Examination score and first-time passing rates for the Iranian Board of	The results of this study demonstrated the clinical mentorship program as an effective method in improving theoretical, clinical, and professional achievement of anaesthesiology residents. Programmed mentorship could significantly improve the educational goal achievements for anaesthesiology residents. Mentoring programs are needed to be more used; however, need to be assessed and evaluated, especially regarding the professional aspects of education. Medical schools could subsequently be assessed regarding the number and quality of their running mentorship programs as a quality improvement tool.

										Anaesthesiology written exam (0.55, P < 0.05).	
Darbyshire et al. 2019	Trainee and supervisor experience of the Academic Foundation Programme	The Academic Foundation Programme is often the initial step along the Integrated Academic Training pathway in the United Kingdom. It is relatively new and research as to its effectiveness is limited. Our objective was to evaluate the Academic Foundation Programme in terms of its impact on academic career aspirations and to explore trainees' expectations and experience of the programme and investigate the enablers and barriers to success.	Residents	Academic Foundation Program, United Kingdom	Not indicated	Primary	Qualitative	Cross-sectional survey	Impact on academic career aspirations.	Thirty-four trainees completed online questionnaires. The majority of trainees (94%) did not proceed directly along the Integrated Academic Training pathway to complete Academic Clinical Fellowships, but those who applied to do so were often successful (nine applicants, six successful). Free-text comments revealed an expectation of a more course-like structure to the programme, this is in contrast to the authentic experience of clinical academia, along with its associated challenges, that some of the supervisors reported. The importance of planning and preparation for success was a recurring theme from the supervisor interviews.	The programme is achieving some success in encouraging academic careers. There are several areas that can be improved. Improving the availability of information and guidance for supervisors and facilitating Academic Foundation Doctors to network are both feasible changes that could lead to improvement.
Davies, Tan and Jenkins, 2000	The Current Status of Senior House Officer Postgraduate Education in a Single Region	To assess postgraduate education (PGE) for paediatric senior house officers (SHOs) in a single region.	Senior house officers	United Kingdom	Not indicated	Primary	Quantitative	Cross-sectional survey	Clinical skills, post-graduate examinations	SHOs graded their overall satisfaction with PGE and improvement in their clinical skills during their post, as	Our study has shown that paediatric SHOs in Wales are reasonably satisfied with their postgraduate education, although there is considerable variation between different units.

										an average of 5 and 4 out of 10, respectively. However, there was wide variation between the different units with scores varying between 3 and 9 out of 10.	Several areas have been highlighted which need to be improved if we are to provide SHOs with adequate preparation for the specialist registrar training grade.
Deora et al. 2020	In Reply to the Letter to the Editor Regarding "Mentor- Mentee Relationship in Neurosurgery	Not indicated	Residents and doctors	India	Not indicated	Tertiary	Not indicated	Editorial	Not indicated	Not indicated	Increase in mentoring of women is necessary in order to increase female faculty. The following changes must be made - characterize barriers, identify and eliminate discriminatory practices when recruiting medicals students, training residents, and hiring and promoting of neurosurgeons, promote women into leadership positions within organized neurosurgery, and foster the development of female neurosurgeon role models by training and promoting competent female trainees and surgeons.
Dimitriadis et al. 2012	Characteristics of mentoring relationships formed by medical students and faculty.	"Little is known about the characteristics of mentoring relationships formed between faculty and medical students. Individual mentoring relationships of clinical medical students at Munich Medical	Clinical medical students	LMU Munich, Germany	Network Program One-on-one mentoring	Primary	Mixed methods	Cohort study	Opinions about the potential mentor's role and topics to be discussed with the mentor, as well as expectations regarding frequency, duration, and mode of mentoring.	High-performing students were significantly more likely to participate in the mentoring program (pB0.001). Topics primarily discussed include the mentee's personal goals (65.5%), career planning (59.6%), and experiences abroad (57.6%). Mentees mostly perceived their mentors as counselors (88.9%),	Medical students with strong academic performance as defined by their grades are more likely to participate in formal mentoring programs. Mentoring relationships between faculty and medical students are perceived as a mutually satisfying and effective instrument for key issues in medical students' professional development. Mentoring relationships are a highly effective means of

		School were characterized quantitatively and qualitatively.”								providers of ideas (85.0%), and role models (73.3%). Mentees emphasized the positive impact of the mentoring relationship on career planning (77.2%) and research (75.0%).	enhancing the bidirectional flow of information between faculty and medical students. A mentoring program can thus establish a feedback loop enabling the educational institution to swiftly identify and address issues of medical students.
Dolmans et al. 2002	The impacts of supervision, patient mix, and numbers of students on the effectiveness of clinical rotations	This study investigated whether supervision, patient mix, and numbers of students influence the effectiveness of clinical rotations. The authors administered a questionnaire to 1,208 medical students in 1999 to evaluate the rotations' quality. They computed four variables - overall effectiveness, supervision, patient mix, and number of students contemporaneously involved - and analysed the data using analysis of variance.	Medical students	Medical School in Maastricht, the Netherlands	Group	Primary	Quantitative	Cross-sectional survey	Students’ perception of clinical rotation’s effectiveness	Supervision and patient mix positively influenced a rotation's effectiveness. A higher level of supervision and a higher level of patient mix led to higher overall effectiveness scores. The number of students did not significantly influence the effectiveness score, although a low number led to a higher score as compared with a high number. Furthermore, the two-way interaction between supervision and patient mix was significant. Supervision more strongly influenced the rotation's effectiveness when patient mix was limited than when it was high. Also, when the patient mix was limited and supervision was high, the average effectiveness score was still 7.7 (scale 1–10).	The effectiveness of clinical rotations depends on the supervision provided and patient mix, but not the number of students. Furthermore, supervision more strongly influences effectiveness when patient mix is limited than when it is high. Finally, high-quality supervision guarantees at least a sufficient score for the rotation's effectiveness, irrespective of the level of patient mix.
Dornan et al. 2007	Experience-based learning: A model linking the processes and	To develop a model linking the processes and outcomes of	Medical students	University of Manchester, United Kingdom	Group	Primary	Qualitative	Model from Grounded Theory Analysis	Learning processes (participation in practice) and	To reach their ultimate goal of helping patients, medical students must develop 2 qualities. One	The core condition for clinical workplace learning is ‘supported participation’, the various outcomes of which

	outcomes of medical students' workplace learning.	workplace learning. We synthesised a model from grounded theory analysis of group discussions before and after experimental strengthening of medical students' workplace learning. The research was conducted within a problem-based clinical curriculum with little early workplace experience, involving 24 junior and 12 senior medical students.							outcomes (practical competence and a state of mind that includes confidence, motivation and a sense of professional identity).	is practical competence; the other is a state of mind that includes confidence, motivation and a sense of professional identity. These 2 qualities reinforce one another. The core process of clinical workplace learning involves 'participation in practice', which evolves along a spectrum from passive observation to performance. Practitioners help students participate by being both supportive and challenging. The presentation of clear learning objectives and continuous periods of attachment that are as personal to the student(s) and practitioner(s) as possible promote workplace learning.	are mutually reinforcing and also reinforce students' ability to participate in further practice. This synthesis has 2 important implications for contemporary medical education: any reduction in medical students' participation in clinical practice that results from the patient safety agenda and expanded numbers of medical students is likely to have an adverse effect on learning, and the construct of 'self-directed learning', which our respondents too often found synonymous with 'lack of support', should be applied with very great caution to medical students' learning in clinical workplaces.
Dorsey et al. 2019	Relationships Matter: Enhancing Trainee Development with a (Simple) Clerkship Curriculum Reform	The traditional clerkship model of brief encounters between faculty and students results in reduced meaningful learning opportunities due to the lack of a relationship that enables repeated observation, supervisor	Medical students	United States of America	Group	Primary	Mixed methods	Intervention, Surveys, Focus Group	Student experience at the clerkship<br><br>Student participation (number of common obstetric procedures)	Students in the revised clerkship performed twice the number of obstetric procedures. Objective measures (United States Medical Licensing Examination Step 1 scores, receiving clerkship honours, self-reported interest in obstetrics, and gender) did not correlate with the number of procedures performed by students. Qualitative analysis of	Clinical curricular reforms that strengthen the continuity of the supervisor–trainee relationship promote mutual trust and can result in a more meaningful training experience in less time. Reciprocal engagement and early alignment of goals between supervisor and trainee are critical for creating a positive relationship.

		feedback, trust formation, and growth.								student survey comments revealed that procedure numbers were influenced by being proactive, having a supervisor with a propensity to teach (trust), and contextual factors (busy service or competition with other learners). Themes identified by faculty that influenced student participation included relationship continuity; growth of patient care skills; and observed student engagement, interest, and confidence. The quality of the relationship was cited by both students and faculty as a factor influencing meaningful clinical participation. Discussions of “rich pictures” drawn by students and faculty revealed that relationships are influenced by continuity, early alignment of goals, and the engagement and attitude of both student and faculty.	
Drolet et al. 2014	A mentorship-based preclinical elective increases exposure, confidence, and interest in surgery.	To investigate if earlier exposure to operative experiences and the establishment of resident mentors through a preclinical elective would	Medical students	Alpert Medical School of Brown University, United States of America	Formal program	Primary	Quantitative	Pre-post study survey	Confidence and interest in surgery	The study found significantly improved confidence (2.8 vs 4.4) and clinical exposure (2.4 vs 4.3) before versus after the elective, with pre-course scores equal to matched peers.	This elective incorporates elements that have been shown to positively influence student decision making in surgical career choice. The mentorship model promotes residents as educators, whereas the elective provides a means for early

		enhance student confidence and interest in surgery.									identification of students interested in surgery.
Edwards and Cozens, 2019	Establishing a regular systems-based teaching programme for core medical trainees within a district general hospital	Although Health Education and Improvement Wales provide regional teaching days for core medical trainees in line with the curriculum, the local teaching for core medical trainees within hospitals in Aneurin Bevan University Health Board has been variable. Focusing on the Royal Gwent Hospital where no formal teaching was being run locally, I aimed to establish regular teaching sessions for core medical trainees incorporating clinical skills, simulation training and useful systems-based discussions that are relevant to day to day working practices.	Residents	Hospitals in Aneurin Bevan University Health Board, United Kingdom	Group Formal program	Primary	Qualitative	Cohort study	Resident satisfaction and clinical skills.	To date, the teaching sessions have been rated overall by attendees as ‘excellent’ or ‘very good’. Two-thirds of people felt the clinical skills workshops in particular improved their clinical skills ‘a great amount’ and all attendees agreed that these workshops would help them pass their Annual Review of Competency Progression for their level of training.	Establishing a regular local teaching programme for core medical trainees has had its challenges, particularly driving a culture towards positive change among trainees and consultant colleagues in a hospital which has traditionally had no formal teaching. Despite the challenges faced, the outcome of establishing this varied systems-based teaching programme has proved to be beneficial to trainees to date, helping them to gain confidence in both academic knowledge and clinical skills which can be utilised as they progress into higher training posts

		The aim is to drive cultural change in a hospital that has never run local teaching sessions for core medical trainees to establish a regular programme that can be continued beyond August 2019 with the new introduction of internal medicine trainees in Wales.									
Edwards et al. 2001	Use of a journal club and letter writing exercise to teach critical appraisal to medical undergraduates.	There is growing interest in methods of teaching critical appraisal skills at undergraduate and postgraduate levels. We describe an approach using a journal club and subsequent letter writing to teach critical appraisal and writing skills to medical undergraduates.	Medical students	New South Wales, Australia, University of Newcastle medical school	Programm e	Primary	Mixed methods	Intervention, discussion, critical appraisal	Informal and formal feedback from students; number of letters written, submitted and published, and a comparison of marks.	Feedback from students was overwhelmingly positive. In the first 3½ years, 26 letters have been published or accepted for publication, and 58 letters published on the Internet. There were no significant differences in overall marks or marks for the critical appraisal component of the literature review assignments between the two student groups.	We believe our approach is an innovative and enjoyable method for teaching critical appraisal and writing skills to medical students. Lack of difference in marks in the literature review between the student groups may reflect its insensitivity as an outcome measure, contamination by other critical appraisal teaching, or true ineffectiveness.
El-Gabri et al. 2019	Surgical Coaching for Advancement of Global Surgical Skills and Capacity: A Systematic Review	Surgical coaching is an emerging concept of education and collaboration to improve surgical performance. Surgical	Junior doctors	United Kingdom	Not indicated	Tertiary	Not indicated	Systematic Review	Not indicated	Not indicated	Not indicated

		education in low-resource settings remains a challenge because of confounding barriers of access, resources, and sustainability. No formal assessments of coaching as a means to improve surgical quality in low- or middle-income countries exist. The purpose of this review is to explore if surgical coaching could serve as an effective method of fostering continued education and advancement of surgical skills in low-resource settings.									
Ericsson et al. 2004	Deliberate practice and the acquisition and maintenance of expert performance in medicine and related domains.	In this article, I propose an alternative framework to account for individual differences in attained professional development, as well as many aspects of age-related decline.	Not indicated	Not indicated	Not indicated	Tertiary	Mixed methods	Commentary	Treatment Outcomes	The distinctive aspect of the expert-performance approach to the study of expertise is its focus on identifying superior, reproducible behaviour for representative tasks in the associated domain. The behaviours in medicine that most clearly capture the essence of expertise are effective treatments of	In sum, our understanding of expert medical performance will improve as we apply the expert-performance approach to the study of reproducibly superior performance in medicine. The study of expert performance in medicine is particularly likely to provide unique insights for future applications to many types of professional expertise, more

		This framework is based on the assumption that acquisition of expert performance requires engagement in deliberate practice and that continued deliberate practice is necessary for maintenance of many types of professional performance.								medical conditions and diseases. Unfortunately treatments for common diseases are often highly standardized and, thus, offer only limited room for individual doctors to influence outcomes and exhibit superior performance.<br><br>A successful integration of the broad body of research on expertise in a professional domain, such as medicine, requires the recognition of differences between the professional domain and more traditional domains of expertise.	generally. The domain of medicine offers a unique opportunity for research on expert performance, in that the benefits of improved medical diagnosis and treatment are quite obvious.
Erridge et al. 2017	Telementoring of Surgeons: A Systematic Review	Telementoring is a technique that has shown potential as a surgical training aid. Previous studies have suggested that telementoring is a safe training modality. This review aimed to review both the technological capabilities of reported telementoring systems as well as its potential benefits as a mentoring modality.	Residents	United Kingdom, Norway	Not indicated	Tertiary	Not indicated	Systematic Review	Not indicated	66 studies were identified for inclusion. 48% of studies were conducted in general surgery. 22 (33%), 24 (36%) and 20 (30%) of studies reported telementoring that occurred within the same hospital, outside the hospital and outside the country respectively. 64 (98%) of studies employed video and audio and 38 (58%) utilized telestration. 12 separate studies directly compared telementoring against on-site mentoring. 7 (58%) showed no difference in outcomes between telementoring and on-	The results of this review suggest that telementoring has a similar safety and efficacy profile as on-site mentoring. Future analysis to determine the potential benefits and pitfalls to surgical education through telementoring are required to determine the exact role it shall play in the future. Technological advances to improve remote connectivity would also aid the uptake of telementoring on a larger scale.

										site mentoring. No study found telementoring to result in poorer post-operative outcomes.	
Esbenshade et al. 2020	Mentors' perspectives on successes and challenges of mentoring in COG Young Investigator Mentorship Program: A report From the Children's Oncology Group.	Identification and development of Young Investigators is critical to the long-term success of research organizations. In 2004, the Children's Oncology Group created a mentorship program to foster the career development of Young Investigators (faculty <10 years from initial appointment). This study sought to assess mentors' long-term assessment of this program.	Junior doctors	Children's Oncology Group, United States, Canada, Australia, New Zealand, and Europe	Dyadic Formal	Primary	Quantitative	Cross-sectional	Career impact and research project.	The response rate was 74.2%. As some mentors had multiple mentees, we report on 138 total mentee/mentor pairs. Mentors were 57.4% male, and mentees were 39.1% male. Mentors rated being mentored as a Young Investigator as important with median rating of 90 on 1–100 scale. Most mentors reported that being mentored themselves helped their own success within Children's Oncology Group (78.2%) and with their overall career development (92.1%). Most mentors enjoyed serving in the program (72.3%), and the median success rating (on a 1–100 scale) across the mentor/mentee pairings was 75. Success ratings did not differ by mentor/mentee gender, but improved with increased frequency of mentor/mentee interactions. Mentor/mentee pairs who set initial goals reported higher success ratings than those who did not. Tangible successes included:	These data indicate that mentorship is important for successful professional development. Long-term mentoring success improves when mentors and mentees set goals upfront and meet frequently.

										current mentee Children's Oncology Group committee involvement (45.7%), ongoing mentor/mentee collaboration (53.6%), and co-authored manuscript publication (38.4%).	
Eubanks et al. 2020	Speed Mentoring: An Effective and Efficient Path to Development of Mentor Relationships in a Military Obstetrics and Gynecology Residency	With a deficit of effective military residency mentorships, a paucity of research on successful mentorship programs, and growing reports on innovative mentoring programs, we developed a "Speed Mentoring" event for the National Capital Consortium Obstetrics and Gynaecology Residency.	Residents	United States of America	Dyadic Formal program	Primary	Mixed Methods	Pre-post survey	Assessment of structure and execution.	Prior to our first event, every resident reported by survey that they desired more mentorship opportunities. However, only 55% could identify a specific mentor, citing limited time and difficulty establishing a relationship. Immediately following the event, 90% of residents scheduled a follow-up with at least one mentor. Forty-seven percent of residents reported inspiration to initiate a new research project. Meanwhile, faculty felt valued and gained satisfaction by "giving back" to their profession. After 1 month, half of the residents and faculty had already conducted at least one meeting. At 3 months, 76% of meetings cantered on research and 23% on quality improvement projects. Fifty-seven percent of participants reported future scheduled meetings. At	After demonstrating a need for improved mentorship opportunities, we implemented an efficient way to foster mentorship while expanding resident involvement in research, projects, and fellowship applications. This "Speed Mentorship" program can be easily adapted to all residency programs.

										6 months, 75% of residents reported meaningful mentorship relationships.	
Fallowfield et al. 2002	Efficacy of a Cancer Research UK communication skills training model for oncologists: a randomised controlled trial	Doctors' communication with patients is commonly hampered by lack of training in this core skill. This study aimed to assess the efficacy of an intensive 3-day training course on communication skills in a randomised controlled trial with a two-by-two factorial design and several outcomes.	Doctors working in cancer centres	United Kingdom	Network formal programm e	Primary	Quantitative	Randomised controlled trial	Communication behaviours, course attendance	Difference between course attenders [n=80] and non-attenders [n=80] 34%, p=0.003), focused and open questions (27%, p=0.005), expressions of empathy (69%, p=0.003), and appropriate responses to patients' cues (38%, p=0.026), and a 24% lower rate of use of leading questions (p=0.11). There was little evidence for the effectiveness of written feedback.	The communication problems of senior doctors working in cancer medicine are not resolved by time and clinical experience. This trial shows that training courses significantly improve key communication skills. More resources should be allocated to address doctors' training needs in this vital area.
Fallowfield et al. 2003	Enduring impact of communication skills training: results of a 12-month follow-up	The efficacy of a communication skills training programme was shown through a randomised trial. Oncologists (N=160) from 34 cancer centres were allocated to written feedback plus course; course alone; written feedback alone or control. Each clinician had 6 - 10 interviews with patients videotaped at	Oncologists	United Kingdom	Network Formal program	Primary	Quantitative	Focus group	Changes in communication behaviours.	No demonstrable attrition in those who had shown improvement previously, including fewer leading questions, appropriate use of focused and open-ended questions and responses to patient cues. Additional skills, not apparent at 3 months, were now evident; the estimated effect sizes corresponded to 81% fewer interruptions (P=0.001) and increased summarising of information to 38% (P=0.038). However, expressions of empathy	The overall results show that 12 - 15 months postintervention, clinicians had integrated key communication skills into clinical practice and were applying others. This is the first RCT to show an enduring effect of communication skills training with transfer into the clinic.

		baseline and 3 months postintervention. Analysis of videotapes revealed improvements in the communication skills of clinicians randomised to training (n=80) compared with others (n=80). A 12-month follow-up assessment is reported here.								(54%, P=0.001) declined.	
Falowski et al. 2020	Implementation of the North American Neuromodulation Society Mentorship Program: Recommendations and Survey Results of Participants	Despite Accredited Counsel of Graduate Medical Education (ACGME) guidance and criteria, there remains variability in training both within each specialty and across the specialties involved in the delivery of neuromodulation. North American Neuromodulation Society advocates for the efficacious and safe the implementation of neuromodulation and therefore an	Residents	North America	Dyadic Formal program	Primary	Quantitative	Cohort study	Knowledge of neuromodulation and increase in experiences.	There was a 70%-86% response rate on each survey administered. All except one respondent reported that the mentorship program met their previously declared expectations. All the respondents self-reported at least a moderate increase in their knowledge in the field of neuromodulation while 54% of the respondents felt their knowledge in the field to have greatly increased. Most respondents reported an increase in the number of spinal cord stimulator trials and permanent implants performed after the mentorship program. The self-reporting of mentees competencies at the conclusion of the	The North American Neuromodulation Society mentorship program met expectations and implementation goals by improving neuromodulation education including covering patient care, delivery, and training topics. The mentoring program provides a structured framework for extending formal physician neuromodulation education outside of direct fellowship training.

		educational mentoring program with a defined educational platform is needed.								program was statistically significant for higher competency scores in all areas assessed.	
Farkas et al. 2019	Mentorship of US Medical Students: a Systematic Review	Mentoring of medical students remains a core pillar of medical education, yet the changing landscape of medicine has called for new and innovative mentoring models to guide students in professional development, career placement, and overall student well-being. The objective of this review is to identify and describe models of mentorship for US medical students.<br><br>The purposes of this review are to identify and describe models of mentorship for US medical students, to summarize the benefits and/or disadvantages of each, and to	Medical students	United States of America	Dyadic Group	Secondary	Mixed methods	Systematic review	Survey based, with satisfaction being the most measured outcome.<br><br>Residency match data, board scores, or clinical evaluations.	Our search yielded 3743 unique citations. Thirty articles met our inclusion criteria. There was significant diversity of the identified programs with regard to program objectives and size. The traditional dyad model of mentorship was the most frequently cited model, with a combination of models (dyad and group mentorship) cited as the second most common. Outcome measures of the programs were largely survey based, with satisfaction being the most measured outcome. Overall, satisfaction was highly rated throughout all the measured mentoring pro- grams. Seven programs provided objective outcomes measures, including improved residency match data and increased scholarly productivity. The programs with objective outcomes measures were smaller, and 6/7 of them focused on a specific clinical area.	Our review demonstrates that mentoring programs for medical students can positively improve medical school satisfaction and career development. These results underscore the need for continued innovative mentoring programs to foster optimal student development in the setting of the increasingly competitive residency match process, growing expectations about student research productivity, and the national focus for overall student wellness.

		assess barriers to mentorship.								Five of these programs relied on the traditional dyad model of mentor-ship. Cost and faculty time were the most frequently cited barriers to these programs.	
Farnan et al. 2012	A systematic review: the effect of clinical supervision on patient and residency education outcomes	To summarize the literature regarding the effect of clinical supervision on patient and educational outcomes, especially in light of the recent (2010) Accreditation Council for Graduate Medical Education report that recommends augmented supervision to improve resident education and patient safety.	Residents	Not indicated	Not indicated	Secondary	Mixed	Systematic review	Patient outcomes, education-related outcomes	Twenty-four articles across a variety of specialties (i.e., psychiatry, emergency medicine, surgery, anesthesia, and internal medicine) met inclusion criteria. Studies demonstrated that enhanced supervision in already-supervised activities resulted in improved patient- or education-related outcomes. Studies were limited by small sample sizes, nonrandomized designs, and a lack of objective measures of clinical supervision.	Enhanced clinical supervision of trainees has been associated with improved patient- and education-related outcomes in published studies. Future work should focus on developing validated measures of the effects of clinical supervision.
Farrington et al. 2019	Clinical Debrief: learning and well-being together	Clinical environments can be so stressful to medical students as to be detrimental to their learning and well-being. Our intervention, Clinical Debrief, integrates learning through clinical experience with the development of positive coping	Medical students	Not indicated	Group	Primary	Mixed methods	Focus groups	Rating for clinical debrief sessions.	A total of 1857 evaluations were extracted (response rate 67%). The median (interquartile range) overall rating for Clinical Debrief sessions was 9 (8/10), where 10 indicates "excellent" and 1 indicates "significant improvement needed". The rating for the supervisory aspects of the sessions and free-text comments were positive.	Students appreciated safe environments, the session structure, facilitator role modelling, transitional support and processing of emotional experiences. Mandatory integrated longitudinal supervision, using trained clinician facilitators, was positively received by students in transition to clinical placements. Normalising the emotional impact of medical work destigmatises distress. Linking clinical reasoning

		strategies. Students shared cases and experiences during weekly small group classroom discussions, facilitated by general practitioners (from outside their current hospital placement), throughout two consecutive 12-week blocks of their first clinical year. Alongside enquiry-based and clinical reasoning learning, we gave students a safe space to reflect on their affect. Our aim was to critically examine students' views in Clinical Debrief. Our aim was to critically examine students' views in Clinical Debrief.									with affective state awareness to contextualise case management, following Mezirow's transformative learning theory, brings added benefit to learning and well-being. Student demand for the expansion of Clinical Debrief is evidence of success.
Faucett, et al. 2020	African American Otolaryngologists : Current Trends and Factors Influencing Career Choice	Identify factors that influence African American physicians to choose a career in otolaryngology. Determine the predominant practice setting	Doctors	United States of America	Dyadic	Primary	Qualitative	Survey	Influencing career choice and practice location and stratified by age group.	The results were reviewed for trends influencing career choice and practice location and stratified by age group. Most African American otolaryngologists are in academic practice and have subspecialty	The findings suggest the continued need for initiatives to increase African Americans in our specialty. Encouraging early exposure, intentional mentoring of students, and development of African American role models who can be mentors

		for African American otolaryngologists who can be role models. Determine if the presence of an African American otolaryngologist in academic setting influences career choice.								fellowship training. Enjoying medical student clerkship was the most frequently cited reason why African Americans chose otolaryngology as a career regardless of age. Early exposure was a driving factor in those 30 to 40 years old. Receiving mentorship was less influential in career choice for all age groups, but there was a positive association between those who were mentored in training and those who mentor faculty.	may help increase the number of African American otolaryngologist faculty. This can help our specialty achieve racial parity in a percentage that matches the number of African Americans in the United States workforce.
Fernande et al. 2019	Evaluation of a Medical Student Research and Career Development Program to Increase Diversity in Academic Medicine	To describe and evaluate an innovative research program supported by the National Institutes of Health, “Promoting Research Opportunities Fully—Prospective Academics Transforming Health” (PROF-PATH), designed to support medical students from groups underrepresented - in-medicine (URM) interested in pursuing academic careers.	Medical students	United States of America	Dyadic	Primary	Mixed methods	Cross-sectional survey, focus group discussion, interview	Students’ self-confidence in research- and career-related skills and abilities.<br><br>Multiple ability domains.<br><br>Seven SCCT domains and increased student academic career self-efficacy.	Of 454 medical students, 343 (75.6%) completed the surveys. According to preprogram surveys, PROF-PATH students (n = 85) were less confident in their ability to find or manage mentor relationships than TRP students (n = 258) and less likely to report having a mentor who provided strong support for their research interests. At program’s end, PROF-PATH students showed greater increases in confidence than TRP students in multiple ability domains. Qualitative analysis of themes indicated that PROF-PATH influenced students through seven SCCT domains and	An innovative program for URM medical students participating in mentored research was successful in supporting academic career interest and academic self-efficacy. Schools motivated to increase diversity in academic medicine should consider adapting PROF-PATH.

										increased student academic career self-efficacy.	
Finest et al. 2003	Long Term Benefits of Communication Skills Training for Cancer Doctors	To assess satisfaction and learning accomplishments after communication skills training courses for cancer doctors in the Nordic countries.	Specialists and general practitioner s who treat cancers	Nordic Cancer Union, Denmark, Finland, Iceland, Norway, Sweden	Formal, skills training course	Primary	Quantitative	Cohort study	Expectations of and satisfaction with the course, perceived confidence and outcome of communication skills	Whereas physicians at baseline most frequently reported a need to learn specific aspects of communication, often with emphasis on psychiatric problems, at follow-up they most typically reported that they had learnt basic communication skills (i.e. to listen and to pose open-ended questions). The majority of the physicians reported at follow-up that they applied the learnt skills to patient-related work, in relation to colleagues and on a personal level.	This study found that physicians who had taken part in a communications skills training programme were generally satisfied both at course completion and at follow-up. The physicians reported that they had learned to apply basic communication skills rather than dealing with specific psychiatric problems. They reported that they applied what they had learned in patient- related work, both in relation to colleagues and on a personal level.
Foguem and Manckoundia, 2019	Experience of medical students mentoring in an acute geriatric unit: Use of evidence-based medicine	In a retrospective study, we studied the learning experience of second and third year undergraduate medical students in the acute geriatric unit of a University Hospital. Students who did not receive Evidence based medicine (EBM) classes had lower grades than those who did in the final theoretical and	Medical students	France	Formal program	Primary	Quantitative	Retrospective study	Grades of students	Students who did not receive Evidence based medicine (EBM) classes had lower grades than those who did in the final theoretical and practical evaluations ($p < .01$)	Our experience suggested that the implementation of EBM in the curriculum through small, structured semiology courses tailored to the specificities of the elderly improved Evidence based practices for undergraduate medical students. EBM should improve instruction and mentoring during undergraduate students' clinical rotation

		practical evaluations ($p < .01$).<br>Our experience suggested that the implementation of EBM in the curriculum through small, structured semiology courses tailored to the specificities of the elderly improved Evidence based practices for under- graduate medical students. EBM should improve instruction and mentoring during undergraduate students' clinical rotation.<br>. The aim of this retrospective study, through the experience of clinical instructor at a university hospital and school of medicine in France, is to compare the impact of two educational approaches to EBM in undergraduate									

		medical students (MS).									
Fornari et al. 2014	Mentoring program design and implementation in new medical schools.	Mentoring is considered a valuable component of undergraduate medical education with a variety of programs at established medical schools. This study presents how new medical schools have set up mentoring programs as they have developed their curricula.	Medical students	United States of America	Not applicable	Primary	Mixed methods	Cross-sectional observational study	1) The current state of mentoring in new medical schools<br>2) Steps in establishing a mentoring program<br>3) Benefits of the mentoring program to both mentors and students<br>4) Challenges of the mentoring program to both mentors and students	The majority of new medical schools had mentoring programs that varied in structure and implementation. Although the programs were viewed as valuable at each institution, challenges when creating and implementing mentoring programs in new medical schools included time constraints for faculty and students, and lack of financial and professional incentives for faculty.	Similar to established medical schools, there was little uniformity among mentoring programs at new medical schools, likely reflecting differences in curriculum and program goals. Outcome measures are needed to determine whether a best practice for mentoring can be established.
Forsetlund, et al. 2009	Continuing education meetings and workshops: effects on professional practice and health care outcomes	BACKGROUND: Educational meetings are widely used for continuing medical education. Previous reviews found that interactive workshops resulted in moderately large improvements in professional practice, whereas didactic sessions did not. OBJECTIVES: To assess the effects of	Doctors	Not indicated	Not indicated	Secondary	Quantitative	Systematic review	Health professional practice behaviour, patient outcomes	In updating the review, 49 new studies were identified for inclusion. A total of 81 trials involving more than 11,000 health professionals are now included in the review. Based on 30 trials (36 comparisons), the median adjusted RD in compliance with desired practice was 6% (interquartile range 1.8 to 15.9) when any intervention in which educational meetings were a component was compared to no intervention. Educational meetings alone had similar effects (median	Educational meetings alone or combined with other interventions, can improve professional practice and healthcare outcomes for the patients. The effect is most likely to be small and similar to other types of continuing medical education, such as audit and feedback, and educational outreach visits. Strategies to increase attendance at educational meetings, using mixed interactive and didactic formats, and focusing on outcomes that are likely to be perceived as serious may increase the effectiveness of educational meetings. Educational meetings alone

		educational meetings on professional practice and healthcare outcomes.								adjusted RD 6%, interquartile range 2.9 to 15.3; based on 21 comparisons in 19 trials). For continuous outcomes the median adjusted percentage change relative to control was 10% (interquartile range 8 to 32%; 5 trials). For patient outcomes the median adjusted RD in achievement of treatment goals was 3.0 (interquartile range 0.1 to 4.0; 5 trials). Based on univariate meta-regression analyses of the 36 comparisons with dichotomous outcomes for professional practice, higher attendance at the educational meetings was associated with larger adjusted RDs ($P < 0.01$); mixed interactive and didactic education meetings (median adjusted RD 13.6) were more effective than either didactic meetings (RD 6.9) or interactive meetings (RD 3.0). Educational meetings did not appear to be effective for complex behaviours (adjusted RD -0.3) compared to less complex behaviours; they appeared to be less effective for less serious outcomes (RD 2.9) than for more serious outcomes.	are not likely to be effective for changing complex behaviours.

Francis et al. 2020	Diversity and Inclusion-Why Does It Matter	The US population is becoming increasingly diverse. Such a nation requires a culturally competent and diverse physician workforce. Minoritized communities have higher rates of disease and receive lower quality care than White people. Women of colour have staggering infant mortality rates compared with their white counterparts. In otolaryngology, racial, ethnic, and socioeconomic disparities have been identified in adult and paediatric populations. Cultural competence, diversity, equity and inclusion alone may not be enough. Without a critical lens toward structural racism sociodemographic-based health disparities will become more	Medical student and doctors	Not indicated	Not indicated	Tertiary	Qualitative	Commentary	Not indicated	In conclusion, a diverse nation requires a culturally competent and diverse physician workforce. Otolaryngology historically has lagged behind other specialties with respect to diversity, equity and inclusion and remains one of the least diverse specialties, with women and racial/ ethnic minorities significantly underrepresented. Without active discourse around diversity, equity and inclusion, there remains a barrier for undergraduate and graduate students and faculty development and achievement. Similar to a labyrinth, issues common for under-represented groups in medicine are complex and multidimensional, requiring a variety of strategies to succeed. Strategies aimed at increasing diversity, equity and inclusion include programs designed to provide mentorship, coaching, and sponsorship. Pipeline efforts, inclusivity on committees, bidirectional communication, and equal pay are additional diversity, equity and inclusion efforts that	The US population is becoming increasingly diverse. In otolaryngology, racial, ethnic, and socioeconomic disparities have been identified in adult and paediatric populations.<br><br>Competent health care systems can improve the efficiency of staff, patient satisfaction, and outcomes of care like unnecessary testing or differences in referral patterns.<br><br>Otolaryngology has historically lagged behind other specialties with respect to diversity, equity and inclusion and remains one of the least diverse specialties as it relates to gender, race, ethnicity and other identities.

		pronounced. Cultural competence values equality, acknowledges historical injustice and incorporates culture into communication, relationship building, and adaptation to meet unique needs. Structural competence values equity and focuses on the elements of health influenced by systems and policy. Additionally, there is a growing recognition that structural competence is essential to the practice of high-quality medicine.								have been successful in increasing representation. Closing the diversity gap is a long-term process; although action should be taken daily and progress measured regularly, culture changes slowly. Focus on performance and promotion. Finally, accept feedback and use it to make refinements—opportunities exist to continually improve diversity, equity, and inclusion efforts.	
Frei et al. 2010	Mentoring programmes for medical students – a review of the PubMed literature 2000–2008	Although mentoring is acknowledged as a key to successful and satisfying careers in medicine, formal mentoring programs for medical students are lacking in most countries. Within the framework of planning a	Medical students	Not indicated	Not indicated	Secondary	Not applicable	Literature review	Types of programs that exist, the objectives pursued by such programs, and what effects are reported	The mentoring programs reported in 14 papers aim to provide career counseling, develop professionalism, increase students' interest in research, and support them in their personal growth. There are both one-to-one and group mentorships, established in the first two years of medical school and continuing through graduation. The	Mentoring is obviously an important career advancement tool for medical students. In Europe, more mentoring programs should be developed, but would need to be rigorously assessed based on evidence of their value in terms of both their impact on the career paths of juniors and their benefit for the mentors. Medical schools could then be monitored with respect to

		mentoring program for medical students at Zurich University, an investigation was carried out into what types of programs exist, what the objectives pursued by such programs are, and what effects are reported.								personal student-faculty relationship is important in that it helps students to feel that they are benefiting from individual advice and encourages them to give more thought to their career choices. Other benefits are an increase in research productivity and improved medical school performance in general. Mentored students also rate their overall well-being as higher. The 11 surveys address the requirements for being an effective mentor as well as a successful mentee. A mentor should empower and encourage the mentee, be a role model, build a professional network, and assist in the mentee's personal development. A mentee should set agendas, follow through, accept criticism, and be able to assess performance and the benefits derived from the mentoring relationship.	the provision of mentorships as a quality characteristic.
Freundlich et al. 2020	Enhancing Māori and Pasifika graduate interest in ophthalmology surgical training in New Zealand/Aotearoa: Barriers and opportunities	Māori, indigenous to New Zealand/Aotearoa and Pacific Peoples indigenous to Pacific Island Nations living in New Zealand,	Medical students and residents	Māori	Dyadic	Primary	Mixed methods	Retrospective quantitative analysis, prospective semi structured interviews	Specialty training influencing factors and prevocational ophthalmology experience.	A total of 64 (6.7%) out of 954 medical graduates from the University of Auckland and University of Otago ranked ophthalmology among their top-three preferred training specialties (2012-2017).	"Māori/Pasifika graduate interest in ophthalmology training was relatively low. Valuable insights include enhancing specialty exposure, mentor development, promoting Māori/Pasifika connections and clarifying training

		experience poorer health outcomes across several ophthalmic conditions. The Royal Australian and New Zealand College of Ophthalmologists have identified indigenous workforce development as a priority.<br><br>The aim of this research was first to determine quantitatively whether factors influencing a career in ophthalmology differed between Māori/Pasifika and non-Māori/Pasifika medical graduates, and, second, to explore qualitatively how Māori/Pasifika medical graduates perceive ophthalmology as a career choice.								Of the 64 graduates, six (9.3%) identified as Māori/Pasifika. No significant difference in influencing factors between Māori/Pasifika and non-Māori/Pasifika students was identified. Both groups ranked intellectual content, procedural skills, specialty exposure and mentorship as highly influential. Qualitative interviews with Māori/Pasifika graduates highlighted positive experiences in ophthalmology but limited exposure over-all. Negative anecdotes and unclear training pathways discouraged Māori/Pasifika interest in Ophthalmology training.	pathways for future graduates.
Gaetke-Udager et al. 2019	Advanced Quality Training in Radiology: Inaugural Report	The study aims to assess the outcomes of a 2-year advanced quality training	Radiology residents	United States of America	Group and Dyadic	Primary	Qualitative	Phenomenological study	2-Day lean healthcare management course RSNA quality essential	All five proposed projects were accepted.. Additionally, 7 more projects were pursued.. Participants worked on	Rich mentor-mentee relationships throughout a 2-year, targeted curriculum with emphasis on hands-on

	of a 2-Year Program	program for radiology residents.							certificates assessed via contributions to grand rounds, oral presentations, poster presentations, publication awards, and process improvements.	nine local and three multicenter process improvements, gave one to two grand rounds each, presented three oral and five poster presentations, generated eight publications, received three awards, formed 14 mentor-mentee relationships, and influenced local and multisite practice patterns. Participants reported gaining firsthand experience in quality improvement principles and developing real-world leadership skills.	quality improvement can produce meaningful results.
Garg et al. 2020	Letter to the Editor Regarding "Mentor-Mentee Relationship in Neurosurgery"	Response to the article titled “Mentor-Mentee Relationship in Neurosurgery” by Deora et al.	Not indicated	Tertiary	Not indicated	Not indicated	Not indicated	Letter to Editor	Not indicated	Not indicated	Despite having a significant impact on mentees’ personal development, academic career, and research productivity, mentorship is still not enough for the career advancement of women and minorities in medicine faculty.<br><br>Mentees should form a network of mentors rather than seek just a single mentor. Multiple mentors can be found in the same department for clinical and research opportunities, as well as different departments like interventional neuroradiology and neurology for neurosurgeons to help increase their understanding of the pathophysiology and thus

											establish a more holistic clinical and research practice.
Gernert et al. 2019	Have A Chat With …: An Innovative Format For Mentoring of Medical Students	Medical students find difficulty in making informed decisions about their professional future. In response, our medical faculty, which includes more than 4000 students, developed a mentoring programme focused on dyadic mentoring between medical students and medical professionals. In establishing this program, we recognized students' increasing need to identify role models and, more specifically, to understand the strategies such models applied to the making of career decisions. The format 'Have a chat with . . .' was implemented in 2016 as a new form of group	Medical students	Germany	Formal program	Tertiary	Not indicated	Letter to the editor	Not indicated	In 2 years, there were 359 applicants and 209 attendees to 21 different discussion rounds. A total of 177 (85%) students filled out an evaluation form. The mean standard deviation (SD) number of students attending was 10 4, and 94% rated the size of the student group as appropriate. Mean SD scores for organisation and location were 1.22 0.50 and 1.10 0.50, respectively, using a 6-point Likert scale (1 = excellent, 6 = poor). This scale was used in all subsequent ratings unless reported otherwise. Overall discussion rounds were given a mean SD score of1.14 0.37. Students strongly voted for the continuation of this project (mean SD score: 1.01 0.25; 1 = totally agree, 6 = totally disagree). Students mostly agreed that these discussion rounds positively impacted their career decisions (mean SD score: 2.10 1.22). They also agreed that	The 'Have a chat with . . .' format seems to be suitable as a mentoring tool with which to support medical students in their career planning. The format is feasible and needs only limited resources. Therefore, we believe it is applicable in different settings, universities and subjects.

		discussion and mentoring.								most open questions about career perspectives (mean SD score: 1.91 1.10) were sufficiently answered. Responses to the question 'Could you imagine working in the represented medical field?' were heterogeneous (mean SD score: 3.00 2.53 on a 10-point Likert scale; 1 = totally agree, 10 = totally disagree). This probably reflected the fact that some students were more interested in the guest's personality or in his or her career decision strategies than in the specific area of work.	
Giancola et al. 2020	Establishing a Mentoring Culture within the Department: The Role of the Chair	Academic medicine literature corroborates the positive impact of mentoring partnerships for faculty; these include enhanced clinical care, teaching, scholarship, leadership development, career satisfaction, promotion, and retention. Choi et al proposed that "a dynamic culture of	Junior faculty members	Department of Pediatrics at Saint Louis University (SLU).	Formal program	Primary	Mixed methods	Post-program surveys and qualitative focus groups.	Descriptive statistics and trend analysis (open-ended questions) were used to assess participant feedback and inform future rollouts.	Out of 52 pairs, a total of 36 mentors (69%) and 40 mentees (77%) responded to the 4 post-surveys. Overall, the program received positive feedback; 97% of respondents would recommend the program to other faculty.	There are key elements and best practices for the chair/leader who wants to champion mentoring and make it a longstanding priority for the department. These include (1) providing structure and resources; (2) visibly championing mentoring; integrating mentoring into recruitment and hiring practices; (4) providing education and professional development regarding mentoring; (5) encouraging and recognizing mentors and mentees; and (6) measuring,

		mentorship is essential to the success of academic medical centers and should be elevated to the level of a major strategic priority.<br><br>However, faculty report dissatisfaction with the mentoring that they receive.									communicating, and celebrating success.
Giancola et al. 2020	Establishing a Mentoring Culture within the Department: The Role of the Chair	This article expounds on previous work by providing department chairs with best practices for instituting successful mentoring programs and eventually, establishing a departmental culture of mentoring that is self-sustaining. It draws from the literature and our experiences in establishing formal programs in multiple departments and institutions,	Junior faculty	Saint Louis University's Department of Pediatrics	Dyadic	Tertiary	Qualitative	Post program surveys, focus group	Extent to which the mentoring partnership increased their job satisfaction, commitment, and motivation.<br><br>Mentees rated the mentors on content expertise, supportive, professional guidance, constructive feedback, and accessibility.	N/A	“There are key elements and best practices for the chair/leader who wants to champion mentoring and make it a longstanding priority for the department. These include (1) providing structure and resources; (2) visibly championing mentoring; (3) integrating mentoring into recruitment and hiring practices; (4) providing education and professional development regarding mentoring; (5) encouraging and recognizing mentors and mentees; and (6) measuring, communicating, and celebrating success.”

		including the Department of Pediatrics at Saint Louis University (SLU).									
Gibson et al. 2020	Ensuring competency in focused cardiac ultrasound: a systematic review of training programs	Focused cardiac ultrasound (FoCUS) is a valuable modality for rapid assessment of cardiac function and volume status. Despite recent viral adoption among physicians, there is limited data on the optimal training programs for teaching FoCUS and yardsticks for determining competency. We conducted a systematic review to investigate on the optimal training strategies, including type and duration, that would allow physicians to achieve basic competency in FoCUS.	Physicians seeking to be competent in FoCUS	Not indicated	Various optimal teaching strategies	Secondary	Not indicated	Systematic review	Not indicated	Data were extracted from 23 studies on 292 learners. Existing FoCUS training programs remain varied in duration and type of training. Learners achieved near perfect agreement (κ > 0.8) with expert echocardiographers on detecting left ventricular systolic dysfunction and pericardial effusion after 6 h each of didactics and hands-on training. Substantial agreement (κ > 0.6) on could be achieved in half this time.	A short training program will allow majority of learners to achieve competency in detecting left ventricular systolic dysfunction and pericardial effusion by FoCUS. Additional sessions are necessary to ensure skill retention, improve efficiency in image acquisition, and detect other pathologies.
Gibson et al. 2020	Ensuring competency in focused cardiac ultrasound: a systematic review	"Focused cardiac ultrasound (FoCUS) is a valuable skill for rapid assessment of cardiac	UG+PG	USA, UK, Europe	Dyadic	Secondary	Not indicated	Systematic review	"Assessment of competency relative to an expert (of focus)"	"Data were extracted from 23 studies on 292 learners. Existing focus training programs remain varied in duration and type of training. Learners	"A short training program will allow most learners to achieve competency in detecting left ventricular systolic dysfunction and pericardial effusion by

	of training programs	function and volume status. Despite recent widespread adoption among physicians, there is limited data on the optimal training methods for teaching FoCUS and metrics for determining competency. We conducted a systematic review to gain insight on the optimal training strategies, including type and duration, that would allow physicians to achieve basic competency in FoCUS."							"formal assessment of competence, such as by expert review or comparison to an expert-performed echocardiogram. "	achieved near perfect agreement (κ > 0.8) with expert echocardiographers on detecting left ventricular systolic dysfunction and pericardial effusion with 6 h each of didactics and hands-on training. Substantial agreement (κ > 0.6) on could be achieved in half this time"	FoCUS. Additional training is necessary to ensure skill retention, improve efficiency in image acquisition, and detect other pathologies".
Gill et al. 2020	Distant supervision of trainee emergency physicians undertaking a remote placement: A preliminary evaluation	Clinical supervision is an indispensible part of post-graduate medical training. Our study assesses emergency medicine trainees' experiences and preferences with regards to distant supervision via information and communication technology (ICT).	Trainee physicians	Australia	6 month placement	Primary	Mixed methods	Focus group	Quantitative data were analysed descriptively using counts and proportions.<br><br>Qualitative data were analysed using the principles of thematic analysis.	Trainees provided care to 1458 patients and communicated with a supervisor for 126 (8.6%) patients. Phone or audio-visual ICT was used for 111 (88.1%) and 12 (9.5%) patients, respectively. The placement was described by trainees as a unique learning experience that demanded independent practice, enhanced their communication and leadership skills and	Trainees reported that distant supervision had mixed effects on their supervision experiences, professional development and on patient management. Trainees used ICT infrequently.

										increased their confidence. However, some disadvantages described were reduced quality and quantity of communication with supervisors, ICT failure and the supervisor's inability to provide hands-on assistance. Perspectives on the essential requirements of a successful remote placement that involved distant supervision were gathered.	
Gilligan et al. 2020	Assessing The Training Needs Of Medical Students In Patient Information Gathering	Effective communication is a cornerstone of competent medical practice but rates of error, patient complaints, and poor clinician job satisfaction suggests there to be room for improvement in this component of medical practice and education.	Experience d clinicians and medical students	Australia	Not indicated	Primary	Qualitative	Cross-sectional interview	Experiences associated with teaching and learning clinical communication skills and identify targets for improvements to addressing these skills in medical curricula.	Four key themes emerged from thematic analysis of the interviews: the importance of experience, the value of role-models, the structure of a consultation, and confidence.	The findings emphasize the need for improvement in teaching and learning communication skills in medicine. In particular, there is a need for target approaches to teaching foundational skills, which can establish a strong foundation before moving into more complex situations, thus preparing students for the flexibility required in medical interviewing. A second area that could be targeted is in the engagement and training of clinicians as mentors and teachers, with the findings from both groups indicating that preparation for teaching and feedback is lacking. Medical programs can improve their teaching of communication skills and draw ideas from other fields s to engage in applicable innovative approaches.

Goelz et al. 2011	Specific training program improves oncologists' palliative care communication skills in a randomized controlled trial	The aim of the study was to demonstrate that COM-ON-p, concise and individualized communication skills training (CST), improves oncologists' communication skills in consultations focusing on the transition to palliative care.	Oncologists	University Medical Center Freiburg, Freiburg, Germany	Dyadic Formal program	Primary	Quantitative	Randomised controlled trial	Communication Skills	Participants in the IG improved significantly more than those in the CG in all three sections of the COM-ON-Checklist: skills specific to the transition to palliative care, global communication skills, and involvement of significant others (all $P < .01$). Differences between the CG and IG on the global items of communication skills and involvement of significant others were also significant ($P < .01$). Effect sizes were medium to large, with a 0.5-point improvement on average on a five-point rating scale.	Physicians can be trained to meet better core challenges during the transition to palliative care through developed concise CST. Generalization and transfer into clinical practice must be proven in additional studies.
Graddy et al. 2020	Longitudinal Resident Coaching In The Outpatient Setting: A Novel Intervention To Improve Ambulatory Consultation Skills	Direct observation with feedback to learners should be a mainstay in resident education. However it is uncommonly done and its impact on consultation skills has rarely been assessed.	Internal Medicine interns	Johns Hopkins Bayview Internal Medicine Residency in Baltimore, MD	Dyadic Formal program	Primary	Quantitative	Randomised controlled Trial	Behaviour checklist	As part of the post-intervention assessment, senior faculty members blinded to intervention and control group assignments assessed videotaped encounters. Coached interns completed an average of 21/23 behaviors from the checklist, while interns from the control group completed 18 ($p < 0.05$). The median overall grade for coached interns was B+, compared to B-/C+ for controls ($p < 0.05$).	Coaching interns longitudinally using a behavior checklist is feasible and associated with improved consultation performance. Direct observation of complete clinical encounters followed by systematic coaching is educationally valuable, however it is time and resource intensive.
Griffiths and Miller 2005	E-mentoring: does it have a	Mentoring for doctors is clearly an important	Doctors	Trent University,	E-mentoring	Tertiary	Qualitative	Commentary	Not indicated	"We would argue that technology supported mentoring within medical	Maybe, e-mentoring and traditional mentoring should not be compared at all.

	place in medicine?	issue and has been the focus of recent debate. We would like to add our own thoughts in one of the more controversial areas— e-mentoring. Over the past decade there has been a substantial increase in work into e-learning support, such as e-moderating,4 and other mentoring and support systems such as online communities of practice. It is not the intention here to explore these literatures but to concentrate specifically on one area—e-mentoring.		United Kingdom						settings complements and extends what is achieved by face-to-face monitoring. Electronic mentors can provide feedback on clinical and medical issues, persona-lised attention, career advice, and encouragement.”	Traditional mentoring is unlikely ever to be replaced. However, new technologies may provide a useful adjunct to the mentoring boundaries.
Guerrasio et al. 2019	The Evolution of Resident Remedial Teaching At One Institution	Residency program directors and teaching faculty put in large amounts of time, resources and energy to provide underperforming at-risk learners with remedial teaching. A remediation program was	Residents	University of Colorado School of Medicine, United States of America	Dyadic Formal program	Tertiary	Not indicated	Program Evaluation	Number of residents being placed on probation	Upon establishment of the program, the authors have observed a decrease in the number of residents being placed on probation, and, of those on probation, more are graduating and obtaining board certification.	Since most studies describing remediation interventions do not describe an explicit framework and structure for remediation or provide long-term outcomes, the authors hope to demonstrate that a standardized framework for remediation can have long-term positive outcomes. We are also hoping that, in 5 to 10 years, ongoing data analysis of this program will

		established and centralized at the University of Colorado School of Medicine in 2006 and 2012, respectively, that consolidated expertise in and resources for learner assessment and individualized teaching for struggling learners, particularly those placed on probation or receiving warning letters. In this Article, the authors aim to describe the development and outcomes of the program and to investigate possible reasons for the improved outcomes.									show significantly improved outcomes for residents who are placed on probation and engage in remediation through a centralized process.<br><br>The multiple advantages of a centralized remediation program appear to include increased expertise in conducting remediation among a smaller group of faculty, a more systematic approach to remediating struggling residents, more accurate diagnosis of learners' deficits, and more consistent and comprehensive development of learning plans.
Guse et al. 2020	Perception Of The Study Situation And Mental Burden During The COVID-19 Pandemic Among Undergraduate Medical Students With And Without Mentoring	The COVID-19 pandemic resulted in drastic changes in medical education and could affect students' mental health and perception of study conditions. Mentoring may have mediating	Medical students	University Medical Center Hamburg-Eppendorf (UKE), Germany	Not indicated	Primary	Quantitative	Cross-sectional survey	Patient Health Questionnaire (PHQ-4) as well as self-developed items.	543 of 1193 (45.5%) completed the survey. 35% of those participated in the g-mentoring and 7% in the e-mentoring. 59% did not participate in any program. More e-mentees than g-mentees and nonparticipants reported clinically unproblematic levels of anxiety and depression	While students feel substantially burdened by the situation and the majority worries about the impact of the pandemic on their studies, they also seem to cope well with the digital course format. Study motivation during COVID-19 decreased among the majority of students regardless of mentoring. These aspects may be

		effects via increasing social support. The University Medical Center Hamburg-Eppendorf (UKE) offers a voluntary general mentoring program for all interested students and a mentoring program for students with excellent course results and scientific interest. We aimed to look into the mental burden and views of their study situation during COVID-19 among students who did or did not take part in in one of the formal mentoring programs.								symptoms. The majority of students (55%) was somewhat worried about the impact of the pandemic on their study situation. Regarding digital teaching students did not feel overburdened by the lack of a fixed schedule and structure, e-mentees even less than g-mentees and nonparticipants. Both g-mentees and nonparticipants were significantly more appreciative of the possible repeated use of the digital teaching materials than e-mentees (both groups M=5.7 vs. M=5.4 in e-mentees, p=.045).	important to address by medical schools interested in developing effective interventions to support students during a pandemic and continuous online teaching.
Gutmann et al. 2019	Characteristics Of Graduating Us Allopathic Medical Students Pursuing A Career In Neurology	This paper aims to identify factors associated with medical students becoming neurologists because, despite the increasing burden of neurologic disorders, there is a growing	Medical students	United States of America	Not indicated	Primary	Quantitative	Deidentified data from the Association of American Medical Colleges Matriculating Student Questionnaire (MSQ) and Graduation Questionnaire (GQ) were	Training and career-related decisions, intent to enter neurology	1,456 out 51,816 students with complete data (2.8%) indicated an intent to enter a neurology residency. Factors associated with an increased likelihood were a student's rating of excellent for their basic neuroscience course and neurology clerkship, participation in an MD/PhD program,	Data from surveys at the entry into and graduation from medical school suggest multiple approaches to inflate the number of medical students entering neurology, including a focus on the student-reported quality of the basic neuroscience course and neurology clerkships, targeted engagement with MD/PhD students, and mentoring

		neurologist shortage.						obtained for the graduation years 2013 to 2014 through 2016 to 2017. Logistic regression was used to assess demographic characteristics and responses to training and career-related questions in association with specialty choice (intent to enter neurology).		majoring in neuroscience or psychology as an undergraduate, a selection response of “content of the specialty was a strong influence on career choice,” and indicating interest in neurology on the MSQ. Factors associated with a decreased likelihood were a higher-priority response on the GQ for salary, work/life balance, and personal fit of the specialty.	programs for students interested in neurology. Efforts to improve salaries for neurologists, to reduce medical school debt, and to improve work/life balance may also help to attract more students.
Haque et al. 2019	Key Attributes of a Medical Learning Community Mentor at One Medical School	The purpose of this study was to discover the aspects required for a successful learning community (LC) faculty member educator of medical students, as well as examine the roles identified by LC faculty in interactions with student learners	Medical students	University of Texas Southwestern School of Medicine, United States of America	Formal program	Primary	Qualitative	Focus group	Elements required for a successful learning community (LC) faculty member educator of medical students	Five major themes emerged: (1) LC faculty characteristics/competency, (2) suggested faculty development methods, (3) factors outside the LC environment influencing student relationships, (4) student attributes influencing teaching techniques, and (5) measuring and improving history and physical skills. Faculty characteristics/competency subthemes included role-modeling, mentoring, and teaching competence. Suggested faculty development methods subthemes included assessing and giving feedback to faculty, peer development, and learning from experts.	The themes with the most extensive discussion among the experienced LC faculty groups may represent qualities to be sought in future mentor recruitment and faculty development. Future studies could build on this study by similarly investigating student perceptions.

										Experienced LC faculty focused more attention on teaching competence and mentoring competence than inexperienced LC faculty.	
Harrison et al. 2020	Development And Implementation of a Sustainable Research Curriculum For General Surgery Residents: A Foundation For Developing A Research Culture	Different methods to incorporate research training during residency are suggested, however, long-term impact is not studied well. This study reports development of a research curriculum with milestones, a long-term outcome and sustainability, and its impact on the overall departmental research culture.	Residents	Wright State University, United States of America	Dyadic Formal program	Primary	Quantitative	Pre-post study	Research output	Following implementation, we observed a significant increase in the number of resident presentations ($p < 0.05$) and higher trends for publications and grant submissions. Medical student research increased significantly in terms of both presentations and publications ($p < 0.05$). Consequently, we observed a significant improvement in the overall department research productivity.	Our resident research curriculum was associated with improved long-term research productivity. It allowed residents to work closely with faculty and medical students leading to more collaboration resulting in an enhanced scholarly environment.
Hauer et al. 2004	Effects of hospitalist attending physicians on trainee satisfaction with teaching and with internal medicine rotations	Hospitalists are increasingly serving as inpatient attendings at teaching hospitals. The educational impact of this new model is unclear. We evaluated the relationship between type of attending (hospitalist vs traditional) and	House staff and 3rd and 4th year medical students in Internal Medicine	Moffitt-Long Hospital and Mount Zion Hospital, United States of America	Group Formal	Primary	Quantitative	Cross-sectional Survey	Evaluations	The overall evaluation completion rate was 91% (1587 of 1742 evaluations) by trainees working with 17 hospitalists and 52 traditional attendings. Trainees reported significantly more overall satisfaction with hospitalists than traditional attendings (8.3 vs 8.0 on a 9-point scale; $P<.001$) and rated hospitalists' overall teaching effectiveness as superior (4.8 vs 4.5	Trainees reported more effective teaching and more satisfying inpatient rotations when supervised by hospitalists. This analysis suggests that hospitalists may possess or accrue a specific inpatient knowledge base and teaching skill that distinguishes them from nonhospitalists.

		trainees' ratings of attending teaching and the overall ward rotation.								on a 5-point scale; P<.001). Perceived overall educational value of rotations was higher with hospitalist attendings (3.9 vs 3.7 on a 5-point scale; P =.04). Trainees evaluated hospitalists' knowledge, teaching, and feedback as superior to that of traditional attendings. There were no significant differences in reports of attendings' interest in teaching or patients, availability, or emphasis on cost-effectiveness.	
Hauer et al. 2005	Medical students' perceptions of mentoring: a focus-group analysis	Characteristics of medical students' mentoring relationships and factors that affect mentoring during medical school are poorly understood. The authors conducted four focus groups with fourth-year medical students to explore what students sought from mentors, perceived barriers to mentoring and suggestions for improvement.	Fourth-year medical students	The University of California, San Francisco, School of Medicine, United States of America	Dyadic	Primary	Qualitative	Focus Group	To find out what students sought from mentors, perceived barriers to mentoring and suggestions for improvement	Students with and without mentors described a mentoring relationship as a personal connection with a faculty member invested in helping the student achieve a personal and professional vision. The short duration of courses, abrupt change from classroom learning to clerkships and limited exposure to clinicians were perceived as barriers to mentoring. Students recommended that the school explicitly promote mentoring with early education about finding mentors, placing the responsibility on students while also expanding the pool of potential mentors.	It is concluded that medical students characterize mentoring in terms of the interpersonal dynamics of the relationship, emphasizing personal connection and advocacy. Educating and empowering students along with faculty education regarding students' needs may improve mentoring.

Hawkins et al. 2014	A mentorship program for final-year students.	Current mentorship programmes in the UK tend to focus predominantly on junior medical students; however, final-year medical students may encounter significant academic and personal pressures. We established a mentoring scheme to provide individualised support for final-year students from a junior doctor mentor. The objectives of this study were to assess the benefits of the scheme and identify areas for future improvement.	Final year medical students	Great Western Hospital in Swindon	Mentoring program	Primary	Mixed methods	Cohort study	The extent at which the mentorship scheme helped in: Feeling comfortable in a ward environment, improving confidence, academic support, procedural skills, history and examination, pastoral support, reducing exam stress, greater sense of belonging, enjoyment of final year experiences.	Key benefits for students were improved confidence, academic support, increased enjoyment and sense of belonging during their final year. Mentors valued the opportunity to gain teaching experience. All doctors and 96 per cent of students would recommend the scheme to a friend. Possible improvements include an introductory lecture alongside the handbook and a bank of 'reserve' mentors to stand-in when a mentor is away.	The mentorship programme was a valuable addition to the final-year experience, with benefits for students and mentors alike. We will be continuing this pro- gramme in the future, and would recommend the adoption of mentorship schemes for other final-year cohorts.
Hee et al 2019	Understanding the Mentoring Environment through Thematic Analysis of the Learning Environment in Medicine	Mentoring's success has been attributed to individualised matching, holistic mentoring relationships (MRs) and personalised mentoring environments	Medical students	Singapore	Formal mentoring	Secondary	Qualitative	Systematic review	Themes on learning environment structure and culture	A total of 4574 abstracts were identified, 90 articles were reviewed, and 58 full-text articles were thematically analysed. The two themes identified were learning environments structure and learning environments culture. Learning environments	Learning environments is the product of culture and structure that influence and are influenced by the tutor-learner-host organisation relationship. Learning environments structure guides the evolving tutor-learner-host organisation relationship whilst the learning environment culture

		(MEs). Whilst there is growing data on matching and MRs, a dearth of ME data has hindered development of mentoring programme. Inspired by studies likening MEs to learning environments (LEs) and data highlighting common characteristics between the two, this systematic review scrutinises reports on LEs to extrapolate the findings to the ME context to provide a better understanding of ME and their role in the mentoring process.								structure regards the framework that guides interactions within the learning environments. Learning environments culture concerns the values and practices influencing learner-tutor-host organisation interactions.	nurtures it and oversees the learning environment structure. Similarities between learning environments and mentoring environments allow learning environment data to inform programme designers of mentoring environment's role in mentoring's success.
Henry-Noel et al. 2019	Mentorship in Medicine and Other Health Professions	The authors describe the development of optimal mentoring relationships, emphasizing the importance of different approaches to mentorship, roles of the mentors and mentees, mentor and mentee benefits, interprofessional	Not indicated	Not indicated	Dyadic, multiple, apprentice, and team-based	Secondary	Qualitative	Review	Not indicated	Not indicated	Themes highlighted include: Approach to mentorship, mentors' roles and responsibility, mentee responsibilities, mentee and mentor benefits, interprofessional mentorship, respect for culture and gender mentorship

		mentorships for teams, gender and mentorship, and culture and mentorship.									
Herr et al. 2020	Aligning the Implicit Curriculum With The Explicit Curriculum in Radiology	As panel members of the The Implicit Curriculum in Radiology Task Force of the Association of University Radiologists-Radiology Research Alliance (AUR-RRA), the authors present a review of the implicit curriculum, exploring its origin and impact on medical education, and on the overall professional development of medical students, post-graduate medical trainees and practicing physicians.	Medical students, post-graduate medical trainees and practicing physicians	Not indicated	Informal program	Secondary	Not indicated	Review	Not indicated	Strategies for recognizing and contending with the implicit curriculum in radiology training are discussed, with a special emphasis on opportunities to leverage its potential through positive role modeling.	The implicit curriculum is a potent force in determining the norms and values that underlie the evolving professional lives of medical students, residents and practicing physicians, including radiologists, and may have a greater effect on the overall education of a physician than the explicit curriculum. When the implicit curriculum is left unexamined and unarticulated, however, students may receive contradictory messages from teachers and institutional leaders who do not entirely practice what they preach. Evincing the contours of the implicit curriculum in this way helps learners to avoid internalizing unprofessional habits; it also allows them to assume traits that align with the objectives of physician education. Increasing awareness, having structured professionalism training in institutions, and positive role modelling play a part in optimizing the implicit curriculum.
Honavar et al. 2019	Mentoring Is A Serious Business	Not indicated	Not indicated	Hyderabad, Telangana, India	Not indicated	Tertiary	Not indicated	Editorial	Not indicated	Not indicated	An ideal mentor should embody and pass on the virtues of professionalism, ethics, values, and the art of medicine to the mentee. Souba describes the roles of

											a mentor include the following: (1) adviser and counselor; (2) friend; (3) agent; (4) teacher/helper; (5) coach; and (6) manager/leader. He further envisages that a mentor should: Motivate; Empower and encourage; Nurture self-confidence; Teach by example;<br>Offer wise counsel and;<br>Raise the performance bar.
Hoskison et al. 2019	A Conversation About the Role of Humiliation in Teaching: The Ugly, the Bad, and the Good	This paper aims to identify the ugly, the bad, and the good in teaching in medical education, based on their experiences as medical students and then educators.	Medical students and educators	University of Oklahoma-Tulsa School of Community Medicine, United States of America	Not indicated	Tertiary	Not indicated	Commentary	Not indicated	Not indicated	Elements of learner-centered education and an autonomy-supportive approach to education.
Hu et al. 2020	Reflections: Starting an Otolaryngology Medical Student Interest Group	A reduction in applicants for otolaryngology residency<br>has been noted. Some possible factors include the lack<br>of exposure to otolaryngology, the lack of role models,<br>work-life balance, and the competitiveness of otolaryngology	Medical students	University of British Columbia, Canada	Formal program	Tertiary	Not indicated	Commentary	Not indicated	Not indicated	Starting a medical student interest group in otolaryngology may be an excellent way to<br>foster interest in the specialty

		as a deterrent. Our institution started a medical student interest group to address several of these factors.									
Illes et al. 2000	A model for faculty mentoring in academic radiology	The purpose of this report is to describe the development and implementation of a faculty mentoring program in radiology designed to promote the career development of junior faculty and enhance communication in the department.	Faculty members	Stanford University, United States of America	Formal program	Primary	Quantitative	Pre-post study survey, retrospective analysis	Performance review of junior faculty, program evaluation form	Research and academic development were identified as the areas of greatest importance to the faculty. Research and patient care were most improved as assessed by faculty peers during performance reviews. The schedule of semiannual formal mentoring meetings was reported to be optimal.	The program was implemented to the satisfaction of junior faculty and mentors, and longitudinal performance suggests positive effects. Issues to be contended with include confidentiality and the time needed for mentoring beyond already saturated schedules. Overall, the authors propose that mentoring programs can be an asset to academic radiology departments and a key factor in maintaining their vitality.
Insetta et al. 2020	A Novel Intimate Partner Violence Curriculum for Internal Medicine Residents: Development, Implementation, and Evaluation	Intimate partner violence (IPV) is a widespread problem with hard-hitting health consequences. Literature suggests that internal medicine (IM) residents are unprepared to screen for and address IPV. A curriculum was designed to improve IM residents' knowledge, attitudes, and	Internal medicine residents	Johns Hopkins University School of Medicine, United States of America	Formal group program	Primary	Quantitative	Pre-post study	Knowledge, attitudes (e.g. importance, confidence, comfort) and practices (screening frequency).	Thirty-two residents received IPV training. In comparing precurriculum (n = 29, 91% of total participants) and postcurriculum (n = 28, 88% of total participants) surveys, there was significant improvement in knowledge about IPV ($p < .001$). Post-curriculum, learners reported greater confidence in detecting IPV ($p < .001$), documenting IPV ($p < .001$), and referring to resources ($p < .001$). Participants reported increased comfort with	The curriculum built on residents' knowledge, confidence, comfort, and preparedness in screening for and discussing IPV.

		practices in caring for IPV survivors								managing difficult emotions about IPV in patients (p < .01) and themselves (p < .001) and increased comfort in discussing IPV with female (p < .001) and male (p < .001) patients. Post-curriculum, all respondents felt they were more skillful in discussing IPV and would be more likely to screen for IPV.	
Itani et al. 2005	Surgical resident supervision in the operating room and outcomes of care in Veterans Affairs hospitals	There has been concern that a reduced level of surgical resident supervision in the operating room (OR) is correlated with worse patient outcomes. Until September 2004, Veterans' Affairs (VA) hospitals entered in the surgical record level 3 supervision on every surgical case when the attending physician was available but not physically present in the operating room or the operating room suite. This study assessed the impact of level 3 on risk-adjusted morbidity and	Residents	Veterans' Affairs hospitals, United states of America	Dyadic, Informal	Primary	Quantitative	Cohort	Mortality and morbidity rates of the surgical patient at 30 days after surgery	A total of 610,660 cases were available for analysis. Thirty-day mortality and morbidity rates were reported in 14,441 (2.36%) and 63,079 (10.33%) cases, respectively. Level 3 supervision decreased from 8.72% in 1998 to 2.69% in 2004.	Between 1998 and 2004, the level of resident supervision in the OR did not affect clinical outcomes adversely for surgical patients in the VA teaching hospitals.

		mortality in the VA system.									
J. Bhagia and J. A. Tinsley, 2000	The mentoring partnership	In this article, we review mentoring as a partnership that depends on the fit between teacher and student. We expand the discussion to include the concept of loss in the life cycles of these professional relationships. This article was written to highlight the qualities of a good mentor, to reiterate the importance of mentors, to characterize this relationship as a partnership, and to introduce the concept that this professional relationship often changes or ends.	General population	Not applicable	Not applicable	Not applicable	Not applicable	Commentary	Not applicable	Not applicable	This article was written to highlight the qualities of a good mentor, to reiterate the importance of mentors, to characterize this relationship as a partnership, and to introduce the concept that this professional relationship often changes or ends. If one understands mentoring as a partnership, it is easy to see that a natural course may be one in which the person mentored develops confidence and independence, so that the role of the mentor-teacher evolves from authority to guide to, finally, colleague and companion.? It is also appropriate to acknowledge that the mentoring relationship may end, sometimes unexpectedly. Grieving its loss then is a necessary component of the professional partnership.
Jacob et al. 2020	The "Secret Sauce" For A Mentored Training Program: Qualitative Perspectives Of Trainees In Implementation Research For Cancer Control	Mentored training approaches help increase skills for research through mentoring networks and skill building activities. Capacity for dissemination and implementation	Fellows at the National Cancer Institute	National Cancer Institute, United States of America	Formal networking program	Primary	Qualitative	Focus group	Not indicated	Fellows reported that regular monthly check-in calls with mentors helped to progress their research and that group mentoring structures helped their learning of basic DandI research concepts and their application. Accessible, responsive, and	Mentored training works best when mentoring is structured and coupled with applied learning and when respected and dedicated mentors are on board. Increased scientific collaborations and credibility within a recognized network are important trainee experiences that should be considered when designing,

		(DandI) research in cancer is needed and mentored training programs have been developed. Evaluation of mentored training programs through quantitative approaches provides us with information on "what" improved for participants. Qualitative approaches provide a deeper understanding of "how" programs work best.								knowledgeable mentors were commonly mentioned by fellows as a key to their success in the program. Fellows noted multiple positive outcomes that they attributed to their participation in the program including gaining credibility in the field, a network of peers and experts, and career developments (e.g., collaborative publications and grant funding).	implementing, and sustaining mentored training programs'.
Jochemsen-van der Leeuw, et al. 2014	Assessment of the clinical trainer as a role model: a Role Model Apperception Tool (RoMAT)	Positive role modeling by clinical trainers is important for helping trainees learn professional and competent behavior. The authors developed and validated an instrument to assess clinical trainers as role models: the Role Model Apperception Tool (RoMAT).	GP trainees	Netherlands, Dutch institutes for GP specialty training	Dyadic, Formal program	Primary	Quantitative	Interventional	Caring attitude and effectiveness	Of 328 potential GP trainees, 279 (85%) participated. Of these, 202 (72%) were female, and 154 (55%) were first-year trainees. The RoMAT demonstrated both content and convergent validity. Two components were extracted: "Caring Attitude" and "Effectiveness." Both components had high reliability scores (0.92 and 0.84, respectively). Less experienced trainees scored their trainers significantly higher on the Caring Attitude component.	The RoMAT proved to be a valid, reliable instrument for assessing clinical trainers' role-modeling behavior. Both components include an equal number of items addressing personal (Heart), teaching (Head), and clinical (Hands-on) qualities, thus demonstrating that competence in the "3Hs" is a condition for positive role modeling. Educational managers (residency directors) and trainees alike can use the RoMAT.
Jordan et al. 2019.	Impact of a Mentorship	Burnout negatively	Medical students	Harbor-UCLA Medical	Dyadic	Primary	Mixed methods	Pre-post study	Burnout: emotional	A total of 135 students (intervention = 51;	Students had an increased sense of personal

	Program on Medical Student Burnout	impacts providers' health and patient care. Mentorship has positive effects including stress mitigation. The authors sought to evaluate the impact of a mentorship program on burnout in fourth-year medical students during their 4-week emergency medicine subinternship.		Center and Mount Sinai Hospital, United States of America	Formal program				exhaustion, depersonalization and personal accomplishment.	control = 84) and 59 mentors participated. Intervention students demonstrated decreased EE and DP and increased PA scores, medians of -2 (-4 to 4), -1 (-3 to 2), and 1 (-1 to 4), respectively, compared to controls, median difference of 0 for all subscales. After adjusting for potential confounders, there was no significant difference in EE (mean difference = -0.2 [-0.5 to 0.2], p = 0.4) or DP scores (mean difference = -0.2 [-1.8 to 1.5], p = 0.9). There was a significant difference in PA scores (mean difference = 2.2 [0.1 to 4.3], p = 0.04). Most students felt the program positively impacted their rotation (39/48) and decreased stress (28/48). Students felt that the program provided career guidance and positively impacted their personal and professional development. The majority (34/37) of mentors enjoyed participating. Qualitative analysis revealed five major themes: relationship building, different perspective, knowledge sharing,	accomplishment after implementation of a mentorship program. Both mentors and mentees viewed the program positively and perceived multiple benefits.

										personal fulfillment, and self-reflection.	
Judson et al. 2019	Saving Without Compromising: Teaching Trainees To Safely Provide High Value Care	Hospitals are increasingly shifting toward value-based reimbursement and focusing on cost consciousness and patient experience. These concepts are crucial to high-quality, affordable healthcare. However, physicians are not well-trained in factoring cost and patient experience into clinical decisions and thus may cause patient harm by depriving patients of necessary care. This paper discusses ways for physicians to mitigate this risk by engaging in online high value care curricula, using a “5-Question High Value Care Time Out,” getting mentorship from master clinicians and using clinical	Physicians	University of California, United States of America	Informal dyadic program	Tertiary	Not indicated	Opinion	Not indicated	Not indicated	Attempts at emphasizing cost-consciousness and patient experience are well-intentioned and critically important to improve affordability and quality of health care. However, the practice of high-value care necessitates thoughtful instruction and implementation to avoid risking patient harm.

		decision support tools.									
Kaewpila et al. 2020	Depressive Disorders In Thai Medical Students: An Exploratory Study Of Institutional, Cultural, And Individual Factors	This exploratory qualitative study conducted among Thai medical students aims to investigate factors related to the development of medical students' depression and how these factors interact in contributing to depressive disorders.	Medical Students	Faculty of Medicine Ramathibodi Hospital, Thailand	Not indicated	Primary	Qualitative	Cross-sectional survey	Not indicated	Protective factors against depression were social support, positive relationships, a growth mindset, spiritual and mindfulness practices, and an adequate mentoring program.	The 4P framework of predisposing, precipitating, perpetuating, and protective factors are suggested to understand the onset and development of students' depressive disorders and to identify targets for institutional and educational intervention. Protective factors against depression were social support, positive relationships, a growth mindset, spiritual and mindfulness practices, and an adequate mentoring program.
Kalen et al. 2010	Mentoring medical students during clinical courses: A way to enhance professional development.	The objective of this study was to investigate undergraduate medical students' experiences and perceptions of one-to-one mentoring and whether they felt that the mentorship promoted their personal and professional development.	Medical students	So¨dersjukhus et, Sweden	Formal program	Primary	Mixed methods	Pre-post study survey	The students' professional and personal development, the role of the mentor, their relationship and the content of their conversations	Most of the respondents experienced that the mentoring programme had facilitated their professional and personal development. The role of the mentor was experienced as being more supportive than supplying knowledge. The students appreciated talking to a faculty not connected with their courses. The few barriers to a successful mentorship were mainly related to timing logistics and 'personal chemistry'.	One-to-one mentoring during clinical courses seems to enhance the medical student's professional and personal development. Future studies are needed to get a deeper understanding and knowledge about factors of importance for successful mentorship.
Kalen et al. 2012	The Core of mentorship: medical students' experiences of one-to-one mentoring in a	Mentoring has been used in different health care educational programmes, but the core of	Medical students	Sweden	Formal program	Primary	Qualitative	Cross-sectional cohort study	The meaning of mentorship from the perspectives of undergraduate students	The results comprise three overarching themes: Space, Belief in the future and Transition. Having a mentor gave a sense of security and	The students experienced that the mentoring programme created a space in which the transition process of becoming a doctor was facilitated and in

	clinical environment.	mentorship, i.e., facilitating the development of medical students' professional competence, has not been explored in depth in the literature. In order to create effective and meaningful mentoring programmes, there is a need for deeper knowledge of the meaning of formal mentorship and, for this, the students' experiences are important. A mentoring program was set up where all medical students were offered a mentor during their first clinical courses; years 3–4. The mentors were physicians and their role as mentors was to support the students and act as sounding-boards, not to teach or assess knowledge. This study aimed to get a deeper								constituted a 'free zone' alongside the undergraduate programme. It gave hope about the future and increased motivation. The students were introduced to a new community and began to identify themselves as doctors. We would argue that one-to-one mentoring can create conditions for medical students to start to develop some parts of the professional competences that are more elusive in medical education programmes, such as reflective capacity, emotional competence and the feeling of belonging to a community.	which their motivation and belief in the future became stronger. A continuous relation to a professional in the clinical environment created a feeling of safety. The mentoring pro- gramme gave the students an opportunity to have a dialogue and reflect on feelings and relationships with an experienced clinician. This suggests that it might be relevant to include one-to-one mentoring programmes in undergraduate medical education. A condition we believe is of great importance for creating this type of learning environment, or space, is the neutral role of the mentor, i.e. the mentor should not be responsible for assessing the students' knowledge and skills. Therefore, it is important to clearly define the role of the mentor, both for students and mentors, when introducing such a programme. According to our findings, we also think that one-to–one mentoring can create conditions for medical students to start to develop professional competences which are more elusive and difficult to point out and to integrate in the curriculum: such as reflective capacity, emotional competence and the feeling of belonging to a community.

		understanding of the meaning of mentorship seen from the perspective of undergraduate medical students.									
Kalén et al. 2015	Longitudinal mentorship to support the development of medical students' future professional role: a qualitative study.	Mentoring has been employed in medical education in recent years, but there is extensive variation in the published literature concerning the goals of mentoring and the role of the mentor. Therefore, there is still a need for a deeper understanding of the meaning of mentoring for medical students' learning and development. The aim of this qualitative study is to explore how formal and longitudinal mentoring can contribute to medical students' professional development.	Medical students	Karolinska Institutet, Sweden	Combined group and one-to-one mentoring	Primary	Qualitative	Cross-sectional observational study	The type of space for development regarding the mentorship created and the student's professional development .	The results comprise three themes: Integrating oneself with one's future role as a physician, Experiencing clinical reality with the mentor creates incentives to learn and Towards understanding the professional competence of a physician. The mentorship enabled the students to create a view of their future professional role and to integrate it with their own personalities. The students' understanding of professional competence and behaviour evolved during the mentorship and they made advances towards understanding the wholeness of the profession. This approach to mentorship supported different components of the students' professional development; the themes Integrating oneself with one's future role and Towards understanding the professional competence of a physician can be	Formalized and longitudinal mentoring focusing on the non-medical skills can be recommended to help medical students to integrate their professional role with themselves as individuals and promote understanding of professional competence in the process of becoming a physician.

										regarded as two parallel processes, while the third theme, Experiencing clinical reality with the mentor creates incentives to learn, promotes these processes.	
Kalet et al. 2002	Mentoring for a new era.	Over the course of their education, medical students must develop an identity that involves a deep under- standing of professional principles and the skills to apply these ideals. This task is so important that it cannot be left to informal means and should be cultivated by a structured system that is focused on professionalism. The overall goal of our student-mentoring program is to advance the professional development of our students during the first two years of medical school through regular group meetings with skilled, trained faculty facilitators.	Medical students, faculty members	Master scholars program (MSP)	Workshop s	Primary	Mixed methods	Pre-post study	Program touched on a wide range of topics relating to professional development, such as balancing careers in medicine with family and challenges encountered.	A list of each group's discussion topics is posted on an electronic bulletin board to inform others. Two faculty mentors lead each group to ensure continuity and diversity over the two-year course of their meetings. A total of 57 out of 78 (73%) attended workshops to learn about the program and enhance their facilitation skills. Professional behaviors are explicitly emphasized in the materials outlining expectations of both students and faculty. Students are expected to attend, participate in and lead discussions, be constructive, respectful, and supportive, and accept/act on constructive feedback. Mentors are to facilitate the group's process, and provide feedback and guidance about the students' ideas and passions and the challenges they encounter. The faculty mentors will contribute	Our medical school has committed resources, and our faculty and students have given thought, energy, and enthusiasm to our mentoring program. This innovative new model has the potential to deepen and enrich the culture of medicine by providing a forum and skills for students to reflect on their own professional development and interact in a meaningful way with committed and skilled faculty who share similar interests.

										descriptive material for the dean's letter on each student. We assessed student understanding of the definition of professionalism prior to the mentoring program and will follow up at regular intervals. Formative evaluation of the program includes surveys and focus groups with students and faculty to assess the effectiveness of the group process in accomplishing the stated goals of the program.	
Kelly et al. 2020	Teaching And Learning In General Practice: Ethical And Legal Considerations For GP Teachers And Medical Students	Not indicated	Medical students	Australia	Dyadic Formal program	Tertiary	Not indicated	Editorial	Not indicated	Not indicated	The general practice teaching context differs from the hospital context and presents its own ethical and legal considerations for GP teachers. Being mindful of these considerations will help ensure positive and patient-centred teaching and learning in general practice placements.
Keser et al. 2020	The role of residents in medical students' neurology education: current status and future perspectives	Neurophobia, a well-described fear of neurology, affects medical students worldwide and may be one of the factors contributing to a shortage of neurologists in the United States. Residents spend a considerable	Medical students	McGovern Medical School, United States of America	Not indicated	Primary	Quantitative	Cross-sectional survey	Confidence in managing neurological conditions and interest in pursuing a neurology residency	Medical students reported a statistically significant increase in their confidence in managing neurological conditions and interest in pursuing a neurology residency after their clerkship. There was a significant association between the medical students' overall rotation experience and the residents' teaching	Our study supports that resident-led teaching efforts are important in improving medical students' neurologic education and their interest in neurology. Our data also supports that the interest in neurology increased for medical students after their neurology clerkship. We examined future strategies to implement "nearpeer" teaching activities to enhance the medical

		amount of time with medical students; therefore, we sought to understand better the impact neurology residents have on medical students during their neurology clerkship and their subsequent interest in neurology. We aimed to identify and implement strategies to decrease neurophobia and increase the number of students pursuing neurology as a career.								effectiveness. The overall clerkship experience correlated with the medical students' interest and confidence in neurology. There was a trend towards an increase in residents' teaching effectiveness and students' rotation experience after a resident teaching workshop. Additionally, of note, students who rotated on both and outpatient and inpatient sites during their clerkship reported an increased interest in neurology.	students' neurologic educational experience. These strategies could potentially mitigate neurophobia and ultimately lead to a much-needed increase in future neurologists.
Kilminster et al. 2007	AMEE Guide No. 27: Effective educational and clinical supervision	Not indicated	Doctors	UK, Australia	Not indicated	Tertiary	Not indicated	Guide	Not indicated	Not indicated	There is the need for a definition of and for explicit guidelines on supervision. While there is evidence that supervision is considered to be both important and effective, practice is highly variable. In some cases, there is inadequate coverage and frequency of supervision activities. There is a need for an effective system to address both poor performance and inadequate supervision.

Koea et al. 2021	Affirmative Action Programmes In Postgraduate Medical And Surgical Training—A Narrative Review	This review aims to identify and summarise the literature pertaining to the implementation of affirmative action programmes (AAP) for selection of ethnic minorities and Indigenous peoples into selective specialist medical and surgical training programmes.	Junior physicians	New Zealand	Not indicated	Secondary	Not indicated	Literature review	Not indicated	The initial search retrieved a total of 524 papers of which 44 were included in this review. All studies retrieved were written in English. All included studies were comprised of national audits (n=6), organisational audits (n = 4), consensus methodology (n = 1), narrative reviews (n = 7), university audits (n = 3) project audits (n = 7), viewpoints (n = 8), simulation experiments (n = 3), case surveys (n = 5) and a position paper (n = 1). 15 Studies centred on ethnic minorities in the United States of America,China, Switzerland and United Kingdom; women who identified as an ethnic minority; Indigenous peoples; both ethnic minorities and Indigenous peoples; and people with low socio-economic status.	This review reaffirms that AAP have proven effective at increasing numbers of ethnic minority and Indigenous medical undergraduates but have not been robustly assessed by specialist medical or surgical training programmes. This review proposes a suggested AAP framework underpinned by comprehensive preparation, support and mentorship of candidates prior to initial applications extending beyond training programmes. The primary goal of any AAP in medical or surgical training programmes should be successful graduation of ethnic minority and Indigenous trainees into practice and appointment to faculty member and leadership positions.
Koopman et al. 2005	Views of family medicine department Chairs about mentoring junior faculty	Mentoring can be a key component contributing to the success of faculty. We investigated the attitudes of family medicine department Chairs toward	Junior faculty	United states of America	Formal mentoring programmes	Primary	Qualitative	Qualitative inquiry	Semi-structured interviews	Although most Chairs felt that mentoring was valuable, very few departments had formal mentoring programs. This gap between the great value placed on mentoring and its lack of existence in departments echoes previous	Academic medicine may benefit from more formal mentoring experiences for junior faculty, as it is generally felt to be of value and there is evidence supporting this. This research highlights a possible conflict between requiring everyone to

		mentoring, with emphasis on mentoring female and minority faculty. This qualitative inquiry used semi-structured interviews with 13 Chairs of US departments of family medicine.								findings. The presence of a formal departmental mentoring program did not correlate with the Chairs' own past experience with mentoring, contrary to our expectations.	participate and making the program personalized. In formalizing the mentoring process, there is a potential to limit personal 'fit' in the relationships. Until more senior women and minority faculty are available, cross-gender and cross-ethnicity mentoring will need to be utilized.
Kosoko-Lasaki et al. 2006	Mentoring for women and underrepresented minority faculty and students: experience at two institutions of higher education	Women and minority faculty and students are seriously underrepresented in university and academic healthcare institutions. The role of mentoring has been identified as one of the significant factors in addressing this underrepresentation. We have described the mentoring efforts at two institutions of higher learning in assisting women and minority students and faculty in being accomplished in their academic pursuits.	Faculty members, medical students	Creighton University, Wake Forest University, United States of America	Formal program	Primary	Quantitative	Cross-sectional study	Evaluation form	One-hundred-thirty students and >50 women and minority faculty have participated in the mentoring programs described. The number of participants has increased dramatically over the years and continues to evolve positively. These programs appear to be quite successful in the short term. Further evaluation of measurable outcomes will be necessary to fully determine their true impact.	The mentoring models for women and underrepresented minority faculty and students at Creighton University Health Sciences Schools and Wake Forest University School of Medicine will serve as a guide for other Health Sciences Schools.
Krishna et al. 2020	Combined Novice, Near-Peer, E-	An acute shortage of senior mentors	Medical students	Singapore	Informal group program	Primary	Mixed methods	Cross-sectional survey	Assessing the 5 essential activities in	Thematic analysis of open-ended responses revealed three themes–	CNEP mentoring is an evolved form of novice mentoring built on a

	Mentoring Palliative Medicine Program: A Mixed Method Study In Singapore	saw the Palliative Medicine Initiative (PMI) combine its novice mentoring program with electronic and peer mentoring to overcome insufficient mentoring support of medical students and junior doctors by senior clinicians. A three-phased evaluation was carried out to evaluate mentees' experiences within the new CNEP mentoring program.							mentoring programs which were 1) matching practices, 2) mentoring relationships, 3) the mentoring environment, 4) the mentoring structure and 5) the mentoring culture.	the CNEP mentoring process, its benefits and challenges that expound on the descriptive statistical analysis of specific close-ended and Likert scale responses of the survey. The results show mentee experiences in the PMI's novice mentoring program and the CNEP program to be similar and that the addition of near peer and e-mentoring processes enhance communications and support of mentees.	consistent mentoring approach supported by an effective host organization. The host organization marshals assessment, support and oversight of the program and allows flexibility within the approach to meet the particular needs of mentees, mentors and senior mentors. Whilst near-peer mentors and e-mentoring can make up for the lack of senior mentor availability, their effectiveness hinges upon a common mentoring approach. To better support the CNEP program deeper understanding of the mentoring dynamics, policing and mentor and mentee training processes are required. The CNEP mentoring tool too needs to be validated.
L. Berman et al. 2008	Attracting surgical clerks to surgical careers: role models, mentoring, and engagement in the operating room	Professional supervision (PS), a collaborative relationship that promotes reflection on contextualised work-related issues for the benefit of ongoing learning and development and improved professional practice, is not well understood in the medical context. This	Medical students	United States of America	Group Formal program	Primary	Mixed methods	Post-program survey	Assessing characteristics of the clerkship experience, students' level of interest in pursuing a career in surgery	Students who sutured ($p = 0.001$), drove the camera ($p = 0.01$), stated that they felt involved in the operating room ($p = 0.009$), and saw residents ($p = 0.03$) and attendings ($p = 0.0003$) as positive role models were more likely to be interested in surgery. After adjusting for covariates, students who sutured in the operating room were 4.8 times as likely to be interested in surgery (95% CI, 1.5 to 14.9) and students who	Students who participate actively in the operating room and those who are exposed to positive role models are more likely to be interested in pursuing a career in surgery. To optimize students' clerkship experiences and attract top candidates to the field of surgery, clerkship directors should encourage meaningful engagement of students in the operating room and facilitate mentoring experiences

		study aimed to explore the attitudes and experiences of PS among doctors working in palliative care in New Zealand.								drove the camera were 7.2 times as likely to be interested in surgery (95% CI, 1.1 to 46.8).	
Lanier and Rose, 2008	The Contemporary Medical Community: Leadership, Mentorship, and Career Choices	This study examined the feasibility and focus of a mentoring program from the perspective of medical leaders (leaders) and physicians new to Canada (physicians).	Medical leaders, doctors	Not indicated	Not indicated	Tertiary	Qualitative	Review	Not indicated	Both leaders and physicians recognized that mentorship would support the physician socially, professionally, and emotionally. They told us that mentorship programs should be structured carefully to build trust, allow mentors and mentees some selection, be in geographic proximity where possible, and have transparent rules. While leaders felt that evaluation would be an important part of a mentorship program, the physicians disagreed noting that it would change the nature of the program. Leaders stated that the ultimate evaluation of the program's success would be found in retention numbers.	Physicians new to a country need support. Mentorship is a feasible approach to support new comers that may result in more efficient and effective integration, enculturation, and higher levels of retention.
Larson et al. 2020	From the Historical Examples of Drs. Osler, Cushing, and Van Wagenen: Lessons on the Importance of	In this historical vignette, we describe and exemplify mentoring relationships, while at the same time extract	Not indicated	Not indicated	Not indicated	Secondary	Not indicated	Literature review	Relevant primary and secondary sources that documented the interactions between Cushing, Osler,	Not indicated	Through our review, it is undeniable that Dr. Osler's personal and professional guidance was vital to young Dr. Cushing's success as the founder of modern neurosurgery. Likewise, Cushing's tutelage of Van

	Mentorship in Contemporary Neurosurgery	important and applicable principles from them. We reviewed relevant primary and secondary sources that documented the interactions between Cushing, Osler, and Van Wagenen. In founding the field of neurological surgery, the brilliant yet volatile Dr. Harvey Cushing received guidance from his mentor, Dr. William Osler.							and Van Wagenen.		Wagenen enabled Van Wagenen to become a leader of a second generation of neurosurgeons, thereby perpetuating the existence of Cushing's high neurosurgical standards. These historical mentor-mentee relationships were built on 4 primary components: accurate recognition of talent, guidance, arrangement of opportunity, and sustenance of mentorship—actions that are commonly implicated in effective mentorship in contemporary studies.
Lawlor et al. 2020	Women in Otolaryngology: Experiences of Being Female in the Specialty	A broad survey of women otolaryngologists on the current state of the field, including opportunities for advancement, support of family leave, and prevalence of harassment, has not been performed since 1998. This paper provides an update on the experiences of female otolaryngologists	Residents	American Academy of Otolaryngology–Head and Neck Surgery (AAO-HNS), United States of America	Residency program	Primary	Quantitative	Cross-sectional survey	How women within the specialty perceive the educational and professional environment, individual's feelings about work-life balance.	Five hundred thirty-five responses out of 2303 total WIO members (23.2%). Respondents ranged in age from 25 to >65 years. Respondents reported that in the residency programs they attended, 29% of residents, 13% of faculty, and 7% of department leaders were women. Forty-four percent disagreed that their department leaders and 39% disagreed that their male co-residents were supportive of women starting families in training. Younger	Women otolaryngologists continue to experience harassment in the workplace. It is encouraging that younger otolaryngologists feel more supported by their departments in both their careers and their personal lives. This survey highlights critical areas for continued growth within our specialty.

		is vital to continue to advance the specialty.								respondents were more likely to feel that their department leaders were supportive of female residents, maternity leave, etc. Harassment in the current work environment did not differ by age; 53% reported harassment-free, 31% subtle undertones, 8% noticeable tones, 2% significant harassment. Harassment in the workplace varied by region; the greatest level of harassment was in the Midwest.	
Lempp and Seale, 2004	The hidden curriculum in undergraduate medical education: qualitative study of medical students' perceptions of teaching	To study medical students' views about the quality of the teaching they receive during their undergraduate training, especially in terms of the hidden curriculum.	Medical students	United Kingdom	Undergraduate medical training	Primary	Qualitative	Cross-sectional	Medical students' experiences and perceptions	Students reported many examples of positive role models and effective, approachable teachers, with valued characteristics perceived according to traditional gendered stereotypes. They also described a hierarchical and competitive atmosphere in the medical school, in which haphazard instruction and teaching by humiliation occur, especially during the clinical training years.	Following on from the recent reforms of the manifest curriculum, the hidden curriculum now needs attention to produce the necessary fundamental changes in the culture of undergraduate medical education.
Lennon et al. 2020	Understanding the professional satisfaction of hospital trainees in Australia	Ensuring that specialty trainees are professionally satisfied is not only important from the point of view of trainee wellbeing but also	Junior doctors	Australia	Hospital trainee	Primary	Qualitative	Cross-sectional survey	Professional satisfaction	The three most strongly correlated factors with professional satisfaction were feeling well supported and supervised by consultants (or 2.59; 95% ci 2.42 - 2.77)),	This study suggests that good clinical supervision and support, appropriate working hours and supported study time directly impacts trainee satisfaction, potentially affecting their quality of clinical care. Furthermore the

		critical for health systems to retain doctors. Despite this there has been little systematic research on specialist trainees that identifies policy-amenable factors correlated with professional satisfaction. This study examined factors associated with trainee professional satisfaction in a national australian cohort.								having sufficient study time (or 1.54; 95% ci 1.40 - 1.70) and self-rated health status (1.65; 95% ci 1.53 - 1.80). Those working >56 hrs/wk were significantly less professionally satisfied (or 0.76; 95% ci 0.70 - 0.84) compared to the median work hours (45 – 50 hrs/wk). Those earning in the lower quintiles, earlier in their training and who have studied at an overseas university were also significantly less likely to be satisfied.	needs of junior trainees, overseas graduates and those working >56 hrs/wk should be given particular consideration when developing wellbeing and training programs.
Leslie et al. 2005	Junior faculty experiences with informal mentoring	Mentoring is one way in which new faculty can acquire the skills needed for a successful academic career. Little is known about how informal mentoring is operationalized in an academic setting. This study had two main objectives: (1) to determine if junior faculty identify as having an informal mentor(s) and to describe their informal	General	Canada	Informal mentorship	Primary	Qualitative	Cross-sectional	Interview regarding career development	Four topics recurred: qualities sought in mentors, processes by which guidance is obtained, content of the guidance received and barriers. Faculty obtained guidance in two principal ways: (a) through collegial working relationships; and (b) through discussion with senior clinicians as part of the evaluative system in the department. Participants discussed the degree of mentoring they received in the areas of: career focus, orientation to the organization, transition of role from trainee to	Barriers identified included an evaluative role and conflict of interest on the mentor's part. Junior faculty identify some relationships from which they receive guidance; however, limitations in these relationships result in a lack of mentorship on career direction and on balancing career with personal life.

		mentoring relationships; and (2) to identify the areas in which these faculty seek career assistance and advice.								faculty and work/nonwork balance.	
Liénard et al. 2010	Is it possible to improve residents breaking bad news skills? A randomised study assessing the efficacy of a communication skills training program	This study aims to assess the efficacy of a 40-h training programme designed to teach residents the communication skills needed to break the bad news.	Residents	Belgium	Training berk Formal program	Primary	Quantitative	Cohort study	Communication skills	“”Ninety-five residents were included. After training, the duration of the pre-delivery phase was found to be longer for the trained residents (relative risk (RR) 1⁄4 3.04; Po0.001). The simulated relative’s first turn of speech about the bad news came more often during the pre-delivery phase (RR1⁄46.68; P1⁄40.008), and was more often initiated by the trained residents (RR1⁄419.17; Po0.001). Trained residents also used more assessment (RR 1⁄4 1.83; Po0.001) and supportive utterances (RR 1⁄4 1.58; Po0.001).”	“This study demonstrates that a training programme that focuses on the practice of dyadic and triadic communication skills can improve the communication skills of the participating residents in a BBN triadic consultation. Such a training should be included in resident curriculum.”
Lin et al. 2015	Redesigning a clinical mentoring program for improved outcomes in the clinical training of clerks.	This study assessed the effect of a redesigned clinical mentoring program from the perspective of clerks. The objective was to assess the benefits of the redesigned program and	Medical students	China Medical University Hospital (CMUH), Taiwan	Formal program	Primary	Mixed methods	Pre-post study survey	See Fig 1.	The redesigned mentoring program improved the professional development of students by mentors in areas such as mentor guidance in personal career interests, promotional opportunities, coordinating professional goals, modeling of their behavior after the	The findings serve as an improved framework for the role of the institution and demonstrate how institutional policies, programs, and structures can shape a clinical mentoring program. The authors recommend the adoption of mentorship schemes for other cohorts of medical students and for different learning and training

		identify potential improvements.								mentors; as well as personal support offered by mentors.	stages involved in becoming a physician.
Loo et al. 2017	Towards a Practice Guided Evidence Based Theory of Mentoring in Palliative Care	Provision of end of life care and coping with the emotional and existential distress engendered by palliative care demands the provision of holistic support and training for palliative physicians. Mentoring is an effective means of meeting this need; however little is known of mentoring in palliative care and a universally accepted learning theory of mentoring remains lacking in this setting. To advance mentoring practice in palliative care, we review the only two evidenced based mentoring theories based upon narrative reviews of mentoring practice in the key specialties	General	Singapore	Not indicated	Tertiary	Qualitative	Meta-analysis	Not indicated	Building upon mentoring's mentee, mentor and organizational dependent, goal specific, context sensitive features highlighted in both recent reviews of mentoring this paper proffers a working theory of mentoring. Constructed Krishna's Mentoring Pyramid that underlines the 5 core elements of successful mentoring programs, we propose melding elements of the cognitive apprenticeship model with the adult learning theory using the multi-theories model of adult learning offers an effective starting point for a mentoring theory. More context-specific studies are needed to provide better insight into the validity of this framework in the ongoing pursuit of an interprofessional mentoring theory in Palliative Medicine.	The melded Multi-theories model does appear to encapsulate the key aspects of the mentoring process described by Wu et al. and Wahab et al.. Whilst this framework does offer a good starting point for an effective mentoring theory further studies need to be carried out within the specific context of mentoring involving senior clinicians and junior clinicians and/or undergraduates within the nursing, medicine, surgery and medical social work settings. Further studies should also be carried out to compare the mentoring process in undergraduates and postgraduate settings to further validate the theory particularly given the potential differences in mentoring approaches, goals and duration in addition to obvious differences in mentee characteristics. We believe an effective mentoring theory is now in sight and hope that standardized mentoring experiences defined by this framework will move palliative care education forward.

		within palliative care teams.									
Low et al. 2018	A narrative review of mentoring programmes in general practice	Mentoring in general practice nurtures personal and professional development among mentees and mentors and enhances organisational productivity. Yet, failure to define it leads to variations in understanding and practice and conflation with other educational approaches. As a result, mentoring in general practice is underutilised and poorly overseen. This review seeks to identify elements among successful mentoring relationships to aid the design of local mentoring programmes. Reports of undergraduate and postgraduate mentoring programmes in general practice were analysed thematically.	Medical students and physicians	Singapore	Informal and formal programm es	Secondary	Qualitative	Systematic review	Mentoring relationship	Three themes were identified, including the initiation of mentoring relationships, the mentoring relationship and the mentoring environment. Successful mentoring programmes pivot upon effective mentoring relationships. Based on the themes identified, a framework to guide practice, structure and oversight of the mentoring process is forwarded. Further context- specific study is needed to ensure the viability of the Mentoring Framework to better employ mentoring in general practice.	Drawn from the themes identified in this review, the Mentoring Framework (MF) provides a means of balancing and overseeing the mentoring process. MF also provides a means of designing the mentoring programme

Luckhaupt et al. 2005	Mentorship in academic general internal medicine. Results of a survey of mentors.	Effective mentorship is crucial to career development. Strategies to improve the availability of mentors include mentoring multiple mentees at once, compensating mentors, co-mentoring, and long-distance mentoring. Aim: To describe current trends in mentorship in general Internal Medicine (GIM).	Mentors	University of Cincinnati, United States of America	Formal program	Primary	Quantitative	Cross-sectional survey	Web-based survey (25-iems)	Mentors supervised a median (25th percentile, 75th percentile) of 5 (3, 8) mentees each, and would be willing to supervise a maximum of 6 (4, 10) mentees at once. Compared with mentors without funding, mentors with funding had more current mentees (mean of 8.3 vs 5.1, respectively; P<.001). Full professors had more current mentees than associate or assistant professors (8.0 vs 5.9 vs 2.4, respectively; P=.005). Ninety-four (85%) mentors had experience co-mentoring, and two-thirds of mentors had experience mentoring from a distance. Although most mentors found long-distance mentoring to be less demanding, most also said it is less effective for the mentee and is personally less fulfilling.	Mentors in GIM appear to be close to their mentorship capacity, and the majority lack funding for mentorship. Co-mentoring and long-distance mentoring are common.
Malling et al. 2020	How group coaching contributes to organisational understanding among newly graduated doctors	Practising medicine at an expert level requires skills beyond medical expert knowledge. Research shows that newly appointed consultants feel less prepared to	Junior doctors and residents	Denmark	Group coaching course	Primary	Qualitative	Cross-sectional interview	Experiences and problems in personal lives	Forty-five newly graduated doctors participated in a total of six courses sharing experiences and problems reflected in their professional lives. The following themes emerged: revelation of the hidden curriculum, importance of professional relations,	Newly graduated doctors seemed to develop a growing awareness of organisational issues and a deeper understanding of the complexity of health care organisations through participation in a group coaching course. The study indicates that participation in a group coaching course probably contributes to

		deal with leadership issues compared to issues regarding medical expertice. Newly graduated (ng) doctors and residents in particular struggle with leadership and organisational issues. The purpose of this study was to explore the impact of group coaching on ng doctors' approach to organisational and leadership challenges in daily practice during the transition period from medical school to clinical work.								inter-professional communication, conflict management and emerging leadership skills. Participants' communication skills improved due to an increased awareness of other peoples' perspectives and preferences. They realized the importance of good relations, saw how they could become active contributors in their departments and began to practice leadership skills through involvement of the team, delegation of work and negotiation of own obligations.	improve practice among newly graduate doctors. Further studies are needed to consolidate the findings and explore possible organisational effects.
Mansour et al. 2021	Impact of resident-paired schedule on medical student education and impression of residency programs	Clinical rotations in emergency medicine (em) can be challenging for medical students because of the lack of continuity with attending physicians. To overcome this challenge, institutions have started to match a student's	Medical students	Cook county health and hospital systems, department of emergency medicine, United States of America	Formal programm e resident-paired shifts (rps) and a traditional resident-unpaired shifts (rus)	Primary	Quantitative	Cross-sectional survey	Learning experience and knowledge	The response rate was 47 of 58 students (84%). Respondents indicated that rps resulted in more teaching time (64.6% rps vs 8.3% rus), a better overall educational experience (68.8% rps vs 8.3% rus), and a greater ability to showcase their medical knowledge (52.1% rps vs 6.3% rus). Additionally, students felt that the program was better able	When compared to traditional rus during an ms4 rotation, a rps format provided students with the perception of an improved learning experience, ability to showcase knowledge, and familiarity with the residency program without sacrificing teaching from attending physicians.

		schedule with that of a resident, referred to as "paired shifts." we sought to pilot and compare two schedule formats for fourth-year medical students (ms4) - a resident-paired shifts (rps) and a traditional resident-unpaired shifts (rus) schedule.								to evaluate them (66.7% rps vs 10.4% rus) and they were better able to better evaluate the program (66.7% rps vs 6.3% rus) in the rps format.	
Marcus et al. 2020	Teach and repeat: deliberate practice for teaching	Deliberate practice Facilitates skill mastery. We aimed to Create a novel resident-As-teacher Rotation, leveraging a deliberate Practice framework with repeated Practice in real-life teaching Settings, with feedback from dedicated faculty member coaches.	Resident	Training educators at children's hospitals (teach), United States of America	Formal programme - resident-as-teacher rotation	Primary	Quantitative	Cross-sectional survey	Participant perspective of skills	Survey response rates Varied from 40 to 71%. The rotation was highly valued, and deliberate practice was a most valued aspect. Mean scores in comfort and Confidence significantly increased from pre-To post-rotation, with These increases sustained months later. Prior to implementation, Residents' perceptions of their teaching skills and the adequacy of their training increased incrementally with each postgraduate year. After the inaugural year of the rotation, the PGY2 class rated their teaching skills and training as higher than more experienced residents.	A novel resident-as-Teacher rotation successfully Incorporated deliberate practice in real-life settings by repeating Teaching activities with feedback from dedicated coaches. The Rotation led to sustained increases in residents' confidence in their Teaching, supervising and feedback skills, and improved perceptions of Their teaching training during residency.
Marshall et al. 2019	Womentorship: the	As leaders in academic medicine, we	Physicians	Not indicated	Not indicated	Primary	Qualitative	Commentary	Not indicated	Not indicated	We also recommend that all academic institutions consider offering formal

	#womeninmedicine perspective	have collated several structured recommendations for physicians of both genders seeking to be better mentors to female trainees and early career physicians.									institutional training programs and faculty development in the area of mentorship and sponsorship and that training include discussion of issues specific to women. Such training is extremely important for career development for all physicians and may be especially important to help close the gender gap for women leaders in academic medicine.
Marusic et al. 2003	Teaching Students How to Read and Write Science: A Mandatory Course on Scientific Research and Communication in Medicine	The authors describe the development and introduction of a course on scientific methodology and communication into the medical curriculum in a country outside of the mainstream scientific world.	Medical students	Zagreb University School of Medicine, Croatia	Lectures Group discussion Problem-solving small-group work	Secondary	Qualitative	Cross-sectional survey	Knowledge and attitudes of students	The students' response to the course was overwhelmingly positive, with 96% during the first year feeling that the course provided important education in a stimulating way. In a cross-sectional study of all students from the medical school in Zagreb, we showed that the knowledge on research methodology and attitude toward science was highest in students who just finished the course.	By teaching the principles of scientific research and fostering a positive atti- tude toward it among medical students and academic physicians, we hope to promote responsible conduct of research in the academic environment
Mcdaniel et al. 2019	A qualitative evaluation of a clinical faculty mentorship program using a realist evaluation approach	Clinically focused faculty (full-time clinical faculty and clinician educators) comprise an increasing proportion of academic faculty, yet they underutilize mentorship	Junior physicians	University of Washington, United States of America	Formal, junior faculty mentorship pro- gram	Primary	Qualitative	Cross-sectional interview	Realist evaluation approach via semi structured interviews	We interviewed 23 junior faculty representing 15 pediatric specialties. We identified 4 contextual themes (past personal experience, current competing priorities, institutional culture, and gaps in support and resources), 3 mechanisms (connecting with faculty, sharing	We identified 3 outcomes that participants experienced as a result of the program: creation of a sense of community, acquisition of practical tools and skills, and broadened perspectives.

		nationally. The aims of this study were to test and refine a pro- gram theory for an institutional mentorship program for junior clinically focused faculty and to understand the facilitators and barriers of sustained participation.								ideas and strategies, and self-reflecting), and 3 outcomes (sense of community, acquired tools and skills, and broadened perspectives), which we organized into a programmatic theory representing the program’s impact on participants. Themes that emerged were consistent between both groups.	
Mckenzie et al. 2020	"A taste of real medicine": third year medical students' report experiences of early workplace encounters	This study sought to investigate senior students’ perspectives in their early stages of workplace learning, by using social learning theory as a framework. The focus is on team integration, practical skills performance, professional development and their evolving professional identity.	Medical students	Central clinical school royal prince Alfred hospital, Australia	Workplace learning, by using social learning theory as a framework.	Primary	Qualitative	Focus group	Development of professional identity	Thirty-six students out of 200 (18%) attended focus groups over a four-year period. The results are presented using the theoretical frameworks of community of practice and workplace affordances and presented as themes of: meaning, “learning as experience”, practice, “learning as doing” community, “learning as belonging”, and identity, “learning as becoming”.	Participants reported many positive examples of workplace learning while dealing directly with patients. Students were also exposed to ethical dilemmas and unexpected risks in the workplace. These included lack of site orientation, unsupportive teams, lack of supervision, and students’ inability to initiate agency, all of which contributed to their workplace uncertainty. Performing manageable tasks for their team provided a role in their community of practice, strengthening their identity as evolving doctors. Exposure to both positive and negative role models allowed students to reflect on ethical issues, further extending their own professional identities.
McNamara et al. 2008	A pilot study exploring gender differences in residents’	Mentoring is important throughout a physician's career	Residents	University of Pittsburgh, United States of America	Formal program	Primary	Qualitative	Focus group	Mentoring relationships	Residents of both genders cited multiple barriers to mentoring. Men's strategies for	Female residents may lack strategies and initiatives for finding mentors. Residency programs should create

	strategies for establishing mentoring relationships	and has been noted to be particularly important during residency training. Other studies suggest that women may experience difficulty in finding mentors. This study explored gender-specific differences in residents' mentoring experiences.								finding mentors were more numerous than women's and included identifying mentors through research, similar interests, friendship, and networking. Female strategies were limited and included identifying mentors through "word of mouth" and work experiences. Women described more passive approaches for finding a mentor than men.	opportunities for residents to develop mentoring relationships, with special attention paid to gender differences.
Medina-walpole et al. 2020	A tribute to my mentor on his transition to professor emeritus of medicine	Not indicated	Physicians	Not indicated	Not indicated	Tertiary	Qualitative	Letter to editor	Not indicated	Not indicated	Not indicated
Meeuwissen et al. 2019	Multiple-role mentoring: mentors' conceptualisations, enactments and role conflicts	Outcome-based approaches to education and the inherent emphasis on programmatic assessment in particular, require models of mentoring in which mentors fulfil dual roles: coach and assessor. Fulfilling multiple roles could result in role confusion or even role conflicts, both of which may affect	Medical students	Maastricht university, the Netherlands	Longitudinal and programmatic assessment approaches	Primary	Qualitative	Constructivist Grounded theory study	Competency-based education and Assessment, using the Canadian medical education Directives for specialists (canmeds) roles as its overarching framework.	Three predominant mentoring approaches were constructed: (i) empowering (a reflective and holistic approach to student development); (ii) checking (an observant approach to check whether formal requirements are met), and (iii) directing (an authoritative approach to guide students' professional development). Each approach encompassed a corresponding type of mentor-mentee relationship: (i) partnership; (ii)	In multiple-role mentoring in The context of programmatic assessment, mentors adopted certain predominant mentoring approaches, which were characterised by different strategies for mentoring and resulted in different mentor mentee relationships. Multiple role mentoring does not necessarily result in role conflict. Mentors who do experience role conflict seem to favour the directing approach, which is most at odds with key principles of competency-based education and programmatic assessment. These findings

		mentoring processes and outcomes. In this study, we explored how mentors conceptualise and enact their role in a multiple-role mentoring system and to what extent they experience role conflicts.								instrumental, and (iii) faculty-centred.	build upon existing mentoring literature and offer practical suggestions for faculty development regarding approaches to mentoring in programmatic assessment systems.
Miedzinski et al. 2009	Perceptions of a faculty mentorship programme	Ethics Board-approved anonymous programme evaluation questionnaires were developed. One was mailed to current and past mentees and the other to current and previous academic or scientific mentors. Both groups were asked to evaluate the perceived impact of the relationship on assumed measures of effectiveness, including research funding and publication success, local	Faculty members	Canada	Formal program	Primary	Quantitative	Cross-sectional survey	Ethics Board-approved anonymous programme evaluation questionnaires	Overall, 75 of 138 questionnaires (54%) were returned. These included responses from 41 (65%) academic mentees, 16 (67%) scientific mentees, 31 (62%) academic mentors and 11 (48%) scientific mentors. Whereas most pairs met once or twice per year, 25% of the scientific pairs and 5% of the academic pairs had more than four meetings per year ($P = 0.06$). Significant correlations were noted between the mentee's assessment of adequacy of meeting frequency and perceived mentor helpfulness (academic pairs, $P = 0.01$; scientific pairs, $P = 0.01$). The majority of mentees would recommend their mentors and 82% reported overall	In general, scientific mentoring pairs perceived a greater impact on academic success than academic pairs. Although the overall assessment of the pro-gramme was positive, it was difficult to tease out the specific aspects of academia enhanced by the pro-gramme, which made it difficult to characterise success.

		and national networking, integration into the department, and enhanced life balance.								satisfaction with the programme. A total of 56% of mentees indicated a willingness to serve as mentors in future.	
Mihalynuk et al. 2008	Student learning experiences in a longitudinal clerkship programme.	To identify how medical student learning experiences in a new longitudinally integrated clinical clerkship (LICC) programme impacted students' learning.	Medical students	Not indicated	Longitudinally integrated clinical clerkship (LICC) programme	Primary	Qualitative	Cohort study	Students described clerkship activities and reflected on impact of these activities on their learning.	Students in the LICC programme reported slow but ongoing increases in patient responsibility, examination-driven learning, programme flexibility to address educational gaps, and a strong and positive perception of educational continuity through a longitudinal primary care educator and similar case mix throughout the year.	Student learning experiences in an LICC programme are both similar to and different from those in a traditional rotational clerkship programme. Students in the integrated clerkship were clear and unequivocal about the benefits of working with one teacher across time and caring for patients at different stages of the same disease in multiple settings. These findings have implications for clinical education development and design.
Mikhaiel et al. 2020	Graduating with honors in resilience: creating a whole new doctor	Although coaching programs have become a prominent piece of graduate medical education, they have yet to become an integral part of undergraduate medical education. A handful of medical schools have utilized longitudinal coaching experiences as a method for	Medical students	Georgetown university, Washington, United States of America	Self-directed coaching sessions and workshops	Secondary	Qualitative	Descriptive study	Student testimonials	Of the 132 students in the program, 107 have worked with one of our coaches (81%). Student testimonials have been uniformly positive with students remarking on an increased sense of presence, improvements in communication, and more specific direction in their careers.	Our pilot coaching program has received positive feedback from students early in their medical training. It will be important to further scale the program to reach an increasing number of students and quantitatively evaluate participants for the long-term effects of our interventions.

		professional identity formation, developing emotional intelligence and leadership.									
Moreno et al. 2020	Mentorship strategies to foster inclusivity in surgery during a virtual era	Addressing mentorship in medical school is necessary to pro-mote a diverse and inclusive environment especially now as the ongoing coronavirus pandemic will require prolonged social isolation.	Medical students	Not indicated	Not indicated	Tertiary	Qualitative	Opinion paper	Not indicated	Not indicated	Given the ongoing relative social isolation of the upcoming year, let us make sure we are creating virtual communities so that each student has the necessary network and equipment to access their mentors (e.g., ipads or computers). It is also important not to assume that students have access to virtual platforms and electronic devices, and may require creative alternatives like borrowed computers or electronic medical school resources. Altogether, these strategies will foster inclusion by providing accessible mentors and successful mentoring relationships, especially during a new virtual age.
Murphy et al. 2020	Faculty development: how do we encourage faculty to become better teachers and mentors?	A healthy mentor relationship is a mutually beneficial experience and a necessary part of the natural progression of a career in academic medicine. We sought to explore the advantages of and challenges to becoming a	Medical students and physician trainees	United states of America	Dyadic Formal program	Secondary	Qualitative	Systematic review	Not indicated	Mentorship can promote self-confidence in the ability to choose a career, drive academic productivity, and even inspire a career in academic medicine. It is necessary to help promote advancement in diverse socioeconomic groups within medical trainees. Strong mentors can serve as role models to the next generation of doctors. However, the	A mentor in the current climate of academic medicine must be thoughtful, nimble, and current. They should understand their potential impact on the career choices of a student or trainee and focus on keeping a specialty well-represented by a cross-section of the general population. Finally, they need to be aware of the challenges facing today's students and trainees and

		mentor in current academic urology.								ability to be an effective mentor is being challenged in today's world of academic medicine.	how to navigate these to set these individuals up to succeed.
Nimmons et al. 2019	Medical student mentoring programs: current insights	The aim of our review of the literature, is to analyse current trends in medical student mentoring programs, taking into account their objectives, execution, and evaluation. We outline the challenges encountered, potential benefits, and key future implications for mentees, mentors, and institutions.	Medical students	London, United Kingdom	Dyadic Formal program	Secondary	Qualitative	Systematic review	Not indicated	The quality of evaluation that occurs varies. Few programs follow the students over an extended period of time to assess the long-term impact of a mentoring initiative. The majority of programs use surveys to assess students' experiences and satisfaction, with only a few evaluating tangible outcomes, such as examination results. It is, therefore, hard to establish best practice. Despite this, mentoring has the potential to bring multiple benefits to mentees, mentors, and institutions.	Before a mentoring program is established, a needs analysis or/and pilot should be undertaken to ensure that the design and intended goals are appropriate and achievable. Programs should have clear measurable objectives and outcomes, both short and long term. Mentees and mentors should be matched in a way that encourages their relationship to succeed. This may be through a validated matching process or mentees choosing their own mentor. Mentors should receive training in the requirements of the role and in delivering effective feedback. Incentives should be offered, for example, recognition of mentoring for promotion. Likewise, mentees should be made aware of what is expected of them. Protected time should be allocated for mentoring activities to encourage engagement and motivation. Evaluation should include the mentee, mentor, and institution, and follow the mentee through an extended period of time to assess long-term impact of the initiative. Evaluation should

											utilize validated methods of assessment.
Nothnagle et al, 2010	Promoting self-directed learning skills in residency: a case study in program development	Self-directed learning (SDL) skills are essential for the formation and ongoing competence of today's physicians who work in the context of expanding scientific knowledge and changing health care systems. In 2007–2008, the authors developed a program to promote SDL in the Brown University Family Medicine Residency. Through an iterative process, the project team juggled learning theories (i.e., Knowles' SDL model, Collins' cognitive apprenticeship model, and Quirk's expertise development model) with curricular goals, instructional options, and local	Residents	Brown University Family Medicine Residence, Providence, Rhode Island, United States of America	Dyadic Formal program	Primary	Qualitative	Case study	Measures improvements in residents' SDL skills including the use of EBM, positive impacts on the program learning culture, and favorable changes in resident attitudes regarding lifelong learning.	Not indicated	Although, at this moment, SDL remains more conceptual than practical, its place in the present discourse of medical education is robust, and interventions to amend its relative absence have been reported.

		constraints to design a practical and theoretically robust intervention. The intervention that emerged from this process features a faculty physician serving as a learning coach who meets individually each month with all second- year residents to assist them in generating learning goals, reflecting on their learning experiences, and practicing evidence-based medicine (EBM) skills. An electronic portfolio serves as a documentation tool that supports reflection; residents record their goals and reflections in the portfolio, which also contains their formative assessments, procedure logs, and special projects. To address the hidden									

		curriculum, the program designers took special care to avoid increasing faculty and resident workload and created a forum for discussion and group reflection. Program evaluation combines qualitative and quantitative methods, such as surveys of and interviews with residents and faculty, to assess changes in residents' SDL and EBM skills and in the program's educational culture.<br>The authors use Kern and colleagues' six-step model for curriculum development to describe both the unfolding of this complex project and the choices that resulted in the current program design.									

Oelschlager et al. 2011	Where do medical students turn? The role of the assigned mentor in the fabric of support during medical school	The University of Washington School of Medicine implemented an assigned mentoring program in 2002. The College Mentors are assigned at matriculation, advise students throughout medical school, and teach and evaluate students in the 2nd-year Introduction to Clinical Medicine course. The purpose of the study was to determine from whom students report they would seek advice and support for academic, professional, personal, and research issues.	Medical students	University of Washington School of Medicine, United states of America	Formal program	Primary	Quantitative	Cross-sectional survey	Survey	Students reported that they would contact their College Mentor first for general academic progress (49.6%), personal issues (36.2%), and professional issues (64.1%) but not for research issues.	Students identified their College Mentor as a primary contact for academic, professional, and personal issues, suggesting that neither the mentors' assigned status or evaluator role were barriers to the mentoring relationship.
Ogunyemi et al. 2010	Promoting residents' professional development and academic productivity using a structured faculty mentoring program.	The importance of effective mentoring in career success is universally accepted but has not been well studied in academic medicine. The purpose was to describe a formal	Residents	Cedars Sinai Medical Center, United States of America	Formal mentoring program	Primary	Quantitative	Interventional study	Mentorship profile questionnaire, mentorship effectiveness scale	Twenty-five residents and 18 faculty participated. After 2 years, 7 residents (28%) had scientific publications as compared to 10% in the year preceding the mentoring program. Eleven (44%) presented oral or poster presentations, and 17	A simple but well-organized mentoring system can be an effective means of improving resident learning and enhancing their academic portfolio. Mentoring should be strongly encouraged for residents, especially those with specific deficiencies

		mentoring program of residents by faculty in obstetrics and gynecology and assess professional and educational outcomes.								(68%) were conducting scientific research. Mentors' effectiveness was rated between 4.3 and 4.7 (Likert scale of 1-5). Three of 4 residents in remediation fulfilled their educational requirements. Four residents noted insufficient meeting time.	
Olivier et al. 2020	Lighting a pathway: the minority ophthalmology mentoring program	Racial disparities in eye health and health care are well documented, and in its 2016 report making eye health a population health imperative: vision for tomorrow, the national academies of sciences, engineering, and medicine (nasem) concludes that a diverse physician workforce is an essential element to solving this problem.	Medical students	United States, American academy of ophthalmology (aao) and the association of university professors in ophthalmology (aupo)	Dyadic Formal program	Primary	Qualitative	Commentary/editorial	Increase the number of underrepresented-in-medicine applicants to us ophthalmology-residency programs and support those accepted in the pipeline program. “	Diversifying our workforce will enhance the awareness among all ophthalmologists of health disparities, direct research resources toward understudied medical problems affecting minority communities, and help us provide high-quality patient-centered medicine to all our patients. MOM will set measurable goals for improving the quality of care and ensuring that goals are achieved equitably across all races and ethnicities.	Not indicated
Omary et al. 2008	Mentoring the mentor: another tool to enhance mentorship	Mentoring the mentors provides an alternative “top down” approach to complement the essential and more traditional “bottom up” emphasis that we currently aim to provide to	Teaching faculty	United States of America	Not indicated	Tertiary	Qualitative	Commentary	Not indicated	Not indicated	Highlighting in a proactive fashion the importance of mentoring and of mentoring the mentors helps enrich how our medical societies can further contribute to the well-being of society at large. Implementation of a successful mentoring program is likely to perpetuate and grow the positive effects of

		trainees on the importance of identifying appropriate mentors and what it takes to be role model mentees.									mentorship, and mentoring-the-mentor considerations raised herein are likely to enhance overall mentorship.
Patel et al. 2020	Mentorship and sponsorship: are you in good hands?	Not indicated	Resident	United states of America	Not indicated	Tertiary	Qualitative	Opinion paper	Not indicated	Not indicated	Not indicated
Patel et. al. 2020	A mentor, advisor, and coach (mac) program to enhance the resident and mentor experience	While residents must meet standardized educational milestones to graduate, individualized mentorship and guidance can help them achieve personal and career goals. A novel mentor, advisor, and coach (mac) program was created for residents of the yale university traditional internal medicine residency program to help them attain these goals.	Residents	United states of America	Formal program	Primary	Quantitative	Cross-sectional survey and focus groups	Resident experiences	Survey responses were obtained from 50 of the 116 residents (43%) and 21 of the 49 mac faculty (43%). Thirteen residents and five mac faculty participated in in-person focus groups. Most participants (92% of interns, 83% of residents, and 95% of mac faculty) felt the program was beneficial and should continue. Individualized relationships and meeting content were key to the program’s success. Areas for improvement included clarification of the program’s purpose and each party’s responsibilities in scheduling meetings. Mac faculty also requested faculty development tools to help them meet expectations of being a mac.	The mac program provided a successful avenue for mentorship and guidance for residents. Central themes to enhance participants’ experience were individualization and flexibility, mutual agreement of the ground rules, and enhanced communication from program leadership.

Rabatin et al. 2004	A year of mentoring in academic medicine: case report and qualitative analysis of fifteen hours of meetings between a junior and senior faculty member	We describe a specific mentoring approach in an academic gen-eral internal medicine setting by audiotaping and transcribing all mentoring sessions in the year. In advance, the mentor recorded his model. During the year, the mentee kept a process journal. Qualitative analysis revealed development of an intimate relationship based on empathy, trust, and honesty. The mentor's model was explicitly intended to develop independence, initiative, improved thinking, skills, and self-reflection. The mentor's methods included extensive and varied use of questioning, active listening, standard setting, and frequent	General	Department of Medicine, New York University	Dyadic Formal program	Primary	Qualitative	Case Report and Qualitative Analysis	Not indicated	A mentoring model stressing safety, intimacy, honesty, setting of high standards, praxis, and detailed planning and feedback was associated with mentee excitement, personal and professional growth and development, concrete accomplishments, and a commitment to teaching.	The qualitative analysts identified 4 salient aspects of the mentoring: meetings; themes; interactive qualities; and accomplishments. Successful mentoring is less distinguished by innate personality than by supportive behaviors. To function either as a mentor or as a mentee involves parallel qualities of attending to the process of the relationship, managing conflict effectively, and learning and continuing to be open to possibility.

		feedback. During the mentoring, the mentee evolved as a teacher, enhanced the creativity in his teaching, and matured as a person. Specific accomplishments included a national workshop on professional writing, an innovative approach to inpatient attending, a new teaching skills curriculum for a residency program, and this study.									
Ras et al. 2020	Training family physicians: a qualitative exploration of experiences of registrars in a family medicine training programme in cape town, South Africa	The mmed in family medicine is a professional master's qualification spanning 4 years of training. The outcomes were predetermined by national consensus. While these outcomes are measured in the form of a national exit examination, there has been no exploration of	Junior doctors	Cape Town, South Africa	Not indicated	Primary	Qualitative	Semi-structured interviews, focus group discussion	Perceptions of supervisors and the experiences of registrars	Supervisors identified the strengths and weaknesses of the programme which will impact on further strategic planning. Data from registrar interviews yielded two themes: affirmation, referring to the positive social engagement and facilitation of professional identity formation; and frustrations, referring to structural aspects of the programme which	We now have a better understanding of how structural aspects of the training programme influence the learning environment in beneficial and non-beneficial ways.

		the experiences of registrars (residents) in this relatively new programme. To evaluate the experiences of registrars in one of the nine training programmes in south africa and to identify areas for improvement.								hindered academic progress.	
Razavi et al. 2003	How to Optimize Physicians' Communication Skills in Cancer Care: Results of a Randomized Study Assessing the Usefulness of Posttraining Consolidation Workshops	This study aims to assess the efficacy of six 3-hour consolidation workshops conducted after a 2.5-day basic training program.	Specialists in medical or surgical oncology	Universite´ Libre de Bruxelles, Belgium	Formal, group, consolidation workshops	Primary	Mixed methods	Cohort study	Communication skills (assessed according to the Cancer Research Campaign Workshop Evaluation Manual), patients' perceptions of communication skills improvement (assessed using a 14-item questionnaire).	Sixty-three physicians completed the training program. Communication skills improved significantly more in the consolidation-workshop group compared with the waiting-list group. In simulated interviews, group-by-time repeated measures analysis of variance showed a significant increase in open and open directive questions (P = .014) and utterances alerting patients to reality (P = .049), as well as a significant decrease in premature reassurance (P = .042). In actual patient interviews, results revealed a significant increase in acknowledgments (P = .022) and empathic statements (P = .009), in educated guesses (P	The findings emphasize the usefulness of consolidation workshops to optimize learning of new communication skills and their transfer to clinical practice. As expected, consolidation workshops had a positive impact not only on the range of communication skills learned but also, in some cases, on their amplitude. These improvements highlight a trend toward a physician-patient relationship that is more centered on the patient.

										= .041), and in negotiations (P = .008). Patients interacting with physicians who benefited from consolidation work-shops reported higher scores concerning their physicians' understanding of their disease (P = .004).	
Rodenhauser et al. 2000	Skills for Mentors and Protégés Applicable to Psychiatry	The authors address the elements and dynamics involved in the life cycle of mentoring relationships applicable to psychiatry, including a proposed model for explicating the develop- mental stages experienced by participants in the process. They provide a review of the mentoring literature for use in psychiatric education, research, and practice and offer various perspectives, describing skills for mentors, skills for protege´s, the dynamics of the mentoring relationship, benefits and	Mentors and protégés in psychiatry	United States of America	Dyadic	Secondary	Qualitative	Systematic review	Characteristics of relationship, developmental stages, qualities of a good mentor, benefits and barriers, issues of gender, race, and culture, protégé perspectives, required skills for specific protégé populations	Not applicable	The appreciation of the importance of relationships inherent in the theory and practice of psychiatry encourages exploration of the implications of mentoring as applied to psychiatric education. The benefits of mentoring are not unique to psychiatry, however. The literature on this subject is very broad, and the application of mentoring strategies currently cuts across all denominations in the business and professional worlds. Although mentoring is an integral part of medical students', residents', and junior faculty's development across specialties, psychiatrists are uniquely equipped to contribute to the field of medical education along these lines. The characteristics, benefits, skills, and strategies for mentors and protégés described in this article are generalizable to other medical specialties.

		barriers, and issues related to gender, race, and culture.									
Rothberg et al. 2014	Implementing a resident research program to overcome barriers to resident research	Internal medicine residents are required to participate in scholarly activity, but conducting original research during residency is challenging. Following a poor Match at Baystate Medical Center, the authors implemented a resident research program to overcome known barriers to resident research. The multifaceted program addressed the following barriers: lack of interest, lack of time, insufficient technical support, and paucity of mentors. The program consisted of evidence-based medicine training to stimulate residents' interest in research and structural	Internal medicine residents	Baystate Medical Center, USA	Not indicated	Primary	Quantitative	Cross-sectional study	Research output, number of PGYs in program, number of those accepted in fellowship	Following implementation in the fall of 2005, there was a steady rise in the number of resident presentations at national meetings, then in the number of resident publications. From 2001 to 2006, the department saw 3 resident publications. From 2006 to 2012, that number increased to 39 ($P < .001$). The department also saw more original research (29 publications) and resident first authors (12 publications) after program implementation. The percentage of residents accepted into fellowships rose from 33% before program implementation to 49% after ($P = .04$). This comprehensive resident research program, which focused on evidence-based medicine and was tailored to overcome specific barriers, led to a significant increase in the number of resident Medline publications and improved the reputation	Although we describe numerous interventions in this article, we believe that three elements of the program—leadership, culture change, and excitement—stand above the others. As the research program matured, the residency program attracted trainees with more interest in EBM, and graduating residents obtained competitive subspecialty fellowships. Finally, applicants to the residency program cited the research component as a positive aspect, and some cited it as their reason for applying. In many ways, the problems that we faced initially were solved.

		changes to support their conduct of research, including protected time for research during ambulatory blocks, a research assistant to help with tasks such as institutional review board applications and data entry, a research nurse to help with data collection, easily accessible biostatistical support, and a resident research director to provide mentorship.								of the residency program.	
Rustici et al. 2020	Educational benefits of allowing pediatrician supervision of emergency medicine residents	According to the accreditation council for graduate medical education emergency medicine (em) program Requirements, em residents on em rotations must be supervised by board-certified/board-prepared em or pediatric em	Residents	Denver health emergency medicine, United States of America	Network	Primary	Mixed methods	Cross-sectional observational study	Skill improvement, education and patient outcomes	Ninety percent of residents participated in 1 of 7 focus groups. Four key themes emerged from qualitative analysis of focus group transcripts: (1) pediatricians have unique skills that complement those of em physicians; (2) em resident education improved; (3) patients may get better care with dual staffing; and (4) other	Not indicated

		(pem) faculty. We sought to understand the effect of allowing em residents to be supervised by attending pediatricians while caring for Pediatric urgent care patients.								pem department and urgent care team members may have Benefited from the change.	
Sambunjak et al. 2010	A systematic review of qualitative research on the meaning and characteristics of mentoring in academic medicine	Mentorship is perceived to play a significant role in the career development and productivity of academic clinicians, but little is known about the characteristics of mentorship. This knowledge would be useful for those developing mentorship programs. Aim: To complete a systematic review of the qualitative literature to explore and summarize the development, perceptions and experiences of the mentoring relationship in academic medicine.	Medical students, doctors	North American countries	Mentoring in academic medicine	Secondary	Qualitative	Systemic review	Mentoring relationships	A total of 8,487 citations were identified, 114 full text articles were assessed, and 9 articles were selected for review. All studies were conducted in North America, and most focused on the initiation and cultivation phases of the mentoring relationship. Mentoring was described as a complex relationship based on mutual interests, both professional and personal. Mentees should take an active role in the formation and development of mentoring relationships. Good mentors should be sincere in their dealings with mentees, be able to listen actively and understand mentees' needs, and have a well-established position within the academic community. Some of the mentoring functions aim at the mentees'	Successful mentoring requires commitment and interpersonal skills of the mentor and mentee, but also a facilitating environment at academic medicine's institutions.

										academic growth and others at personal growth. Barriers to mentoring and dysfunctional mentoring can be related to personal factors, relational difficulties and structural/institutional barriers.	
Samer and Zahraa, 2020	Letter to the Editor "Neurosurgical Subspecialty-Tailored Mentoring Approaches: Current Status and Future Demands"	Not indicated	Neurosurgical trainees	Not indicated	Not indicated	Tertiary	Not indicated	Letter to editor	Not indicated	Not indicated	Mentoring plays an indispensable role in training the future batches of neurosurgeons. In light of the rapid expansion of neurosurgical subspecialties, mentoring models need to be adapted to the qualities, demands, and challenges inherent to each neurosurgical subspecialty.
Santen et al. 2019	Factors affecting entrustment and autonomy in emergency medicine: "how much rope do I give them?"	During residency, the faculty's role is to provide supervision while granting the trainee autonomy. This concept is termed entrustment. The goal is appropriate progression from supervision to autonomy while decreasing oversight as residents train. The objective of this study was to better understand the factors affecting the degree of	Residents	Netherlands	Network	Primary	Qualitative	Cross-sectional observational study	Factors affecting faculty Entrustment of patient care to residents	Analysis of the transcripts yielded four major factors affecting entrustment of residents. Patient factors including the acuity of the patient, sociomedical issues of patient/family, and complexity of risk with patient or procedure. Environmental factors including patient volume and systems protocols Resident factors including the year of training, resident performance, clinical direct observation, and patient presentations. Faculty factors including confidence in his/her own practice, risk-averse attitude, degree of	Analysis of the transcripts yielded four major factors affecting entrustment of residents. Patient factors included the acuity of the patient, sociomedical issues of patient/family, and complexity of risk with patient or procedure.

		autonomy or supervision faculty choose to provide residents.								ownership of the patient, commitment to education, and personality.	
Scott et al. 2019	Medical student and faculty perceptions of undergraduate surgical training in the South African and Swedish tertiary institutions: a cross-sectional survey	To evaluate and compare medical student and faculty perceptions of undergraduate Surgical training and compare results between South Africa and Sweden.	Medical students	Africa, Sweden	Group	Primary	Quantitative	Cross-sectional survey	Medical student and faculty perceptions of Undergraduate surgical training	Students believed they ought to receive significantly more teaching when Compared to surgical faculty. Students and faculty generally agreed that students should expect to study approximately six to 20 hrs per week outside of clinical duty. There was general agreement that “small-group tutorials” was the area students learn the most from, whereas students reported “lectures” least helpful. Registrars were reported as the first person students should consult regarding patient care.	Significant differences exist between surgical faculty and medical student perceptions Regarding undergraduate surgical training in developing and developed countries. In order To increase surgical interest among undergraduate medical students, it is imperative for surgical Educators to be aware of these differences and find specific strategies to bridge this gap.
Secchin et al. 2020	Implementation of a Longitudinal Mentorship Program for Quality of Life, Mental Health, and Motivation of Brazilian Medical Students	Mentoring has been used as a strategy for mental health prevention and the promotion of quality of life in medical students, with mixed results. The aim of this study was to compare the levels of mental health, quality of life, and academic motivation of	Medical students	Brazil	Group Formal	Primary	Quantitative	Cohort study	Benefits of the program.	A total of 95 medical students were included: 55 received the mentoring program and 40 did not receive the program. The Multivariate generalised linear model regression model revealed no significant main effect of mentoring. Likewise, the Univariate generalised linear model regression showed no significant main effect of mentoring on medical students’ perceived health.	Mentoring promoted no significant changes in the students of this Brazilian institution. These results highlight the challenges faced in structuring, maintaining, and assessing an effective mentoring program for students on medicine courses.

		medical students after implementation of a longitudinal curricular mentoring program relative to those students without mentoring in their curricula.									
Shah et al. 2020	First-year radiology residents teaching anatomy to first-year medical students: a symbiotic relationship	The purpose of this study is to assess the impact of this program on medical student knowledge and perceptions of radiology, and on resident attitudes toward teaching. Our institution has developed an educational program in which first-year radiology residents teach first-year medical students during gross anatomy laboratory sessions.	Medical students	Department of radiology, University of Pennsylvania, Philadelphia, United States of America	Group	Primary	Quantitative	Cohort study	Students' radiology knowledge, confidence, and perception of radiology.	Students' radiology knowledge significantly increased between the pre-course and post-course. Additionally, there were significant improvements in terms of students' confidence in radiological anatomy skills, perceived importance of radiology for medical training, familiarity with the field of radiology, and perception that radiologists are friendly. Radiology residents felt more confident in their teaching proficiency by the conclusion of the course.	Resident-led small-group teaching sessions during anatomy laboratory are mutually beneficial for medical students and radiology residents. The program also allows radiology residents to be exposed early on in residency to teaching and academic medicine.
Sheng et al. 2020	Supervision of resident physicians	Previous experts have further stipulated supervision to constitute the 3 functions/roles of oversight/management, education, and support. Most of the	Physicians	Department of emergency medicine, Boston medical centre, Boston university school of	Not indicated	Secondary	Qualitative	Review	Factors affecting entrustment of residents, suggestions for optimizing resident autonomy and supervision	The act of supervision entails multiple functions/roles, including oversight / management, education, and support. Despite the limited number of studies on the impact of supervision on patient outcomes, safe, high-quality patient care	Although multiple patient, resident, and environmental dynamics affect entrustment in the supervisory relationship, the supervisor's personality, risk tolerance, and management styles tend to be the deciding factors. Multiple strategies can be implemented on an individual

		medical literature on supervision affords little attention to discussion of theoretic models behind the concept of supervision.		Medicine, United States of America						should be the ultimate collective aim for residents and supervising physicians alike. Residents and faculty should work together to balance autonomy with supervision in order to facilitate learning and development for resident physicians and optimize safe patient care.	physician, departmental, or institutional level to assist supervisors in balancing autonomy with supervision while providing safe, high quality care for patients.
Shojania et al. 2006	Graduate medical education and patient safety: A busy— and occasionally hazardous— intersection.	The case highlights the hazards of patient handoffs as well as the importance of clear communication techniques and knowing when to ask for help.	Graduates in medical education	Ottawa Health Research Institute, University of Ottawa, Ottawa	Not indicated	Tertiary	Not indicated	Report	Not indicated	The discussion also shows the vicious circle that results when attending physicians fail to provide effective supervision: Not only is safety compromised but trainees lose the experience of being supervised. Consequently, trainees have no models of effective supervision on which to draw when they become supervisors. They then fall into the same trap as those who taught them, busying themselves with direct patient care and providing supervision only as time allows.	Not indicated
Sinai J et al. 2001	Developing a training program to improve supervisor–resident relationships, step 1: Defining	By some estimates, the teacher–learner relationship explains roughly half of the variance	Post graduate residents in years 2-5	University of Toronto, United States of America	Dyadic	Primary	Qualitative	Cross sectional	Interactions that either assisted or adversely affected learning	Qualitative analysis of the interview data led to the identification of 5 types of issues affecting the supervisory relationship: goals and individual differences,	Recognition of the types of interpersonal interactions that assist or hinder learning may contribute to enhanced teaching effectiveness.

	the types of issues	attributed to the effectiveness of teaching. Despite this, relation-ships largely have been ignored in the educational literature.								communication and feedback, power and rivalry, support and collegiality, and role modeling and expertise. Face validity was supported when typed anonymous written feedback obtained from annual supervisor evaluations also could be organized into the 5 categories.	
Smith 2020	Surgeon coaching: why and how	The members and leadership of the pediatric orthopaedic society of north america (posna) continue to expand awareness of the impact of burnout on the delivery of care and on the health care professionals that are critical to delivering that care. Surgeon coaching, when appropriately defined, shows considerable promise as a method to create positive change in our team environment and practice, our organizational culture, and our own wellness.	Physicians	Not indicated	Not indicated	Secondary	Not indicated	Literature review	Extensive experience in behavioural health, adult learning theory, and the evolution of best practices as they pertain to coaching techniques and skills	Physician and surgeon coaching is more akin to executive coaching and self-directed learning for highly trained individuals and teams rather than a method of remediation or coercion into someone else's agenda. A methodology for performance improvement to those who have already achieved so much in their careers was shared as a structure for those struggling to organize the process, avoid blind spots, and leverage a growth/reward process rather than the traditional destructive/punitive process that includes shaming, guilting, and other negative techniques. Surgeon coaches and coaching skills are expected to be significant ingredients of performance improvement in team-	High-performance surgeons engage in coaching to maintain or amplify that passion for performance improvement in anything and everything. It does not matter whether you are seeking coaching for juggling the many priorities in your life and practice; dealing with difficult outcomes, litigation, or personal stress; refining a technique or skill; addressing burnout; climbing to the next level of your career; training for or sustaining the marathon of a surgical career; implementing incremental steps or changes; or practicing wellness in your own way. Any way you look at it, coaching and coaching skills can be a positive influence and an avenue to even greater success for surgeons in their life and career.

										based care, organizational culture, and physician wellness.	
Sng et al. 2017	Mentoring relationships between senior physicians and junior doctors and/or medical students: A thematic review.	Mentoring relationships are pivotal to the outcome of the mentoring process. This thematic review seeks to study the key aspects of mentoring relationships between senior physicians and junior doctors and/or medical students to inform efforts to improve mentoring programs.	Senior physicians, junior doctots, medical students	Singapore	Informal or formal programm e	Secondary	Qualitative	Thematic review	Effective mentoring relationships	Thematic analysis of 49 articles reveals five semantic themes of initiation process, developmental process, evaluation process, sustaining mentoring relationship, and obstacles to effective mentoring. The evolving and relational-dependent nature of mentoring pivots upon the compatibility of mentors and mentees and the quality of their interactions, which in turn depend on mentoring environments and awareness of mentor-, mentee-, organizational-related factors and changes in context and goals.	Embrace of a consistent mentoring approach to ensure effective oversight of the mentoring process must be balanced with sufficient flexibility to ensure a mentee-centered approach. Efforts must be made to optimize the key aspects of mentoring relationships in order to ensure successful mentoring processes and outcomes.
Stalmeijer et al. 2008	Cognitive apprenticeship in clinical practice: Can it stimulate learning in the opinion of students?	Learning in clinical practice can be characterised as situated learning because students learn by performing tasks and solving problems in an environment that reflects the multiple ways in which their knowledge will be put to use in their future	Medical students	Maastricht Medical School, Netherlands	Not indicated	Primary	Qualitative	Cross-sectional	Students explored the perceived occurrence of the teaching methods, related problems and possibilities for improvement.	The students had experienced all six teaching methods during their clerkships. Modelling, coaching, and articulation were predominant, while scaffolding, reflection, and exploration were mainly experienced during longer clerkships and with one clinical teacher. The main problem was variability in usage of the methods, which was attributed to teachers' lack of time	The results suggest that the cognitive apprenticeship model is a useful model for teaching strategies in undergraduate clinical training and a valuable basis for evaluation, feedback, self-assessment and faculty development of clinical teachers.

		professional practice. Collins et al. introduced cognitive apprenticeship as an instructional model for situated learning comprising six teaching methods to support learning: modelling, coaching, scaffolding, articulation, reflection and exploration. Another factor that is looked upon as conducive to learning in clinical practice is a positive learning climate. We explored students' experiences regarding the learning climate and whether the cognitive apprenticeship model fits students' experiences during clinical training.								and formal training. The students proposed several ways to improve the application of the teaching methods.	
Stamm and Buddeberg-Fischer, 2011	The impact of mentoring during postgraduate training on	Although mentoring is perceived as key to a successful and satisfying	Final year students, medical school graduates	Switzerland	Formal program	Primary	Quantitative	Cross sectional study	Career success and satisfaction	Up to 50% of doctors reported having a mentor. A significant gender difference was found, with fewer female	This study confirmed the positive impact of mentoring on career success in a cohort of Swiss doctors in a longitudinal design.

	doctors' career success.	career in medicine, there is a lack of methodologically sound studies to support this view. This study made use of a longitudinal design to investigate the impact of mentoring during postgraduate specialist training on the career success of doctors.								than male doctors having a mentor (40.7% versus 60.4% at the fifth assessment; p £ 0.001). Apart from gender, significant predictors of having a mentor were instrumentality (b = 0.24, p £ 0.01) and extraprofessional concerns (b =) 0.15, p £ 0.05). Both having a mentor and having career support from the development network were significant predictors of both objective (b = 0.15, p £ 0.01; b = 0.17, p £ 0.01) and subjective (b = 0.17, p £ 0.01; b = 0.14, p £ 0.05) career success, but not of career satisfaction.	However, female doctors, who are mentored less frequently than male doctors, appear to be disadvantaged in this respect. Formal mentoring programmes could reduce barriers to mentorship and promote the career advancement of female doctors in particular.
Steiner et al. 2004	Assessing the role of influential mentors in the research development of primary care fellows.	To assess the association between mentorship and both subsequent research productivity and career development among primary care research fellows.	Fellows	United States of America	Formal program	Primary	Quantitative	Cross sectional study	Research productivity and career development	A total of 139 fellows (65%) responded to mentorship questions a median of four years after their fellowship. Thirty-seven fellows (26.6%) did not have an influential mentor, 42 (30.2%) reported influential but not sustained mentorship, and 60 (43.2%) had influential and sustained mentorship. Individuals with influential mentorship spent more time conducting research (p = .007), published more papers (p = .003), were more likely to be	Influential and sustained mentorship enhances the research activity of primary care fellows. Research training programs should develop and support their mentors to ensure that they assume this critical role.

										the principal investigator on a grant (p = .008), and more often provided research mentorship to others (72.5% versus 66.7% of those with unsustained mentorship, and 36.4% of those with no influential mentor, p = .008). After controlling for other predictors, influential and sustained mentorship remained an important determinant of career development in research. On qualitative analysis, fellows identified three important domains of mentorship: the relationship between mentor and fellow (such as guidance and support), professional attributes of the mentor (such as reputation), and personal attributes of the mentor (such as availability and caring).	
Taherian and Shekarchian, 2008	Mentoring for doctors. Do its benefits outweigh its disadvantages?	Mentoring is widely used in medicine and is an established means of professional development. We have all been mentored, knowingly or otherwise at some stage of our careers. To provide an overview of mentoring in	Doctors at any stages of their medical journey	Not indicated	Informal and formal programs	Tertiary	Qualitative	Discussion paper	Not indicated	Doctors at all career stages including medical students can benefit from mentoring. Benefits of mentoring include; benefits to the mentee, benefits to the mentor and benefits to the organization. Overall, both mentees and mentors are highly satisfied with mentoring. Nevertheless, problems exist, such as conflict between the mentoring and supervisory roles of	Mentoring is an important developmental process for all involved. There is a perception amongst mentors and mentees that well conducted, well timed mentoring can reap enormous benefits for mentees and be useful to mentors and organizations. However strong evidence for this is lacking and there is need for further research in this area.

		clinical and academic medicine, review the literature, discuss various mentoring styles and weigh the advantages and disadvantages of mentoring.								the mentor, confidentiality breaches, mentor bias, lack of "active listening" and role confusion. Problems usually stem from poor implementation of mentoring. Mentors should not be the mentee's educational supervisor or line manager or otherwise be involved in their assessment or appraisal to avoid blurring of these distinct roles. Safeguards of confidentiality are of vital importance in maintaining the integrity of the mentoring process. Good mentoring is a facilitative, developmental and positive process which requires good interpersonal skills, adequate time, an open mind and a willingness to support the relationship. Mentors should encourage critical reflection on issues to enable mentees to find solutions to their own problems.	
Tan et al. 2018	A framework for mentoring of medical students: thematic analysis of mentoring programmes	A consistent mentoring approach is key to unlocking the full benefits of mentoring, ensuring effective	Medical students	Singapore	Novice mentoring programm es	Secondary	Qualitative	Systematic review	Not indicated	Preparation, initiating and supporting the mentoring process and the obstacles to effective mentoring.	These themes highlight 2 key elements of an effective mentoring framework-flexibility and structure. Flexibility refers to meeting the individual and changing needs of mentees. Structure

	between 2000 and 2015	oversight of mentoring relationships and preventing abuse of mentoring. Yet consistency in mentoring between senior clinicians and medical students (novice mentoring) which dominate mentoring processes in medical schools is difficult to achieve particularly when mentors practice in both undergraduate and postgraduate medical schools. To facilitate a consistent approach to mentoring this review scrutinizes common aspects of mentoring in undergraduate and postgraduate medical schools to forward a framework for novice mentoring in medical schools.									concerns ensuring consistency to the mentoring process and compliance with prevailing codes of conduct and standards of practice.
Thomas et al. 2010	Team training in the neonatal resuscitation program for interns: teamwork	Poor communication and teamwork may contribute to errors during	Junior doctors	United States of America	Not indicated	Primary	Quantitative	Randomised controlled trial	Clinical behaviours (teamwork frequency),	High-fidelity training (HFT) group had higher teamwork frequency than did control subjects (12.8 vs 9.0 behaviors	Trained participants exhibited more frequent teamwork behaviors (especially the HFT group) and better workload

	and quality of resuscitations	neonatal resuscitation. Our objective was to evaluate whether interns who received a 2-hour teamwork training intervention with the Neonatal Resuscitation Program (NRP) demonstrated more teamwork and higher quality resuscitations than control subjects.							resuscitation timings	per minute; P < .001). Intervention groups maintained more workload management (control subjects: 89.3%; low-fidelity training [LFT] group: 98.0% [P < .001]; HFT group: 98.8%; HFT group versus control subjects [P < .001]) and completed resuscitations faster (control subjects: 10.6 minutes; LFT group: 8.6 minutes [P = .040]; HFT group: 7.4 minutes; HFT group versus control subjects [P < .001]). Overall, intervention teams completed the resuscitation an average of 2.6 minutes faster than did control subjects, a time reduction of 24% (95% confidence interval: 12%-37%). Intervention groups demonstrated more frequent teamwork during 6-month follow-up resuscitations (11.8 vs 10.0 behaviors per minute; P = .030).	management and completed the resuscitation more quickly than did control subjects. The impact on team behaviors persisted for at least 6 months. Incorporating team training into the NRP curriculum is a feasible and effective way to teach interns teamwork skills. It also improves simulated resuscitation quality by shortening the duration.
Thomas-MacLean et al. 2010	Discussing mentorship: an ongoing study for the development of a mentorship program in Saskatchewan.	To identify the essential components of a mentorship program as the first step in the ongoing development of a mentorship program for	Junior physicians	Saskatchewa, Canada	Not indicated	Primary	Mixed methods	Cross-sectional	Negative and positive aspects of mentoring	Family physicians described positive and negative aspects of mentoring, or having a lack of experience with mentoring. They also outlined key components of a potential mentorship program: matching mentees with mentors; integrating formal and	Based on the feedback from family physicians, mentorship is viewed as an important and meaningful program of action that regional health stakeholders and medical educators in Saskatchewan could implement. A pilot test of a mentorship program model will be the culmination of this

		primary care physicians.								informal mentorship; and the evaluation process of the mentorship relationship and program.	study. Further research will be undertaken to evaluate the model once it is implemented. This will have important implications for establishing a national mentorship program for family physicians across the country.
Thorndyke et al. 2008	Functional mentoring: a practical approach with multilevel outcomes.	Mentoring is a central component of professional development. Evaluation of “successful” mentoring programs, however, has been limited and mainly focused on measures of satisfaction with the relationship. In today’s environment, mentoring programs must produce tangible outcomes to demonstrate success. To address this issue, the authors advance the framework of functional mentoring combined with measurement of outcomes at multiple levels.	Junior physicians	Penn State College, United States of America	Mentoring program	Primary	Mixed methods	Cohort study	Participation, reaction and satisfaction, the impact of the mentoring relationship, skill development, individual projects (Evidence of transfer/performance) and the impact of the project on the individual and beyond.	In 4 years, 165 faculty participated in the program. Respondents were highly satisfied with the pairings: 85% of junior faculty believed their mentor had a significant effect on their projects. Junior faculty reported a significant enhancement of skills related to initiating and negotiating a new mentoring relationship (85%) and stated that their project would have a significant impact on their career (92%) and on the department or institution (86%).	The success of this mentoring program is demonstrated at multiple levels. The key outcome of functional mentoring is the project. Projects are aligned with professional responsibilities and with institutional missions. The project contributes to the individual’s dossier and adds value to the institution. Functional mentoring is a practical approach that allows measurable results at multiple levels.
Timberlake et al. 2018	Examining the impact of surgical	We examined how problem-	Medical students	United States of America	Not indicated	Primary	Quantitative	Randomised controlled trial	Clinical skills (suturing skills)	No differences in baseline suturing skills	This work suggests that coaching may increase heart

	coaching on trainee physiologic response and basic skill acquisition	solving coaching impacts trainee skill acquisition and physiologic stress as well as how trainee sensitivity to feedback, known as self-monitoring ability, impacts coaching effectiveness.							Self-monitoring skills	between the groups emerged. Improvement in the coaching group's suturing was significantly higher than the control group. One measure of physiologic stress (rmssd) was significantly higher in the coaching group. Trainees who received more coaching demonstrated larger improvements. Overall, perceived quality of the coaching relationship was high. There was no correlation between trainee self-monitoring ability and skill improvement.	rate variability of trainees, indicating coping well with training. Trainee disposition toward feedback did not play a role in this relationship.
Toh et al. 2017	Developing Palliative Care Physicians through Mentoring Relationships	Mentoring has been shown to improve resilience, sense of well-being, reduce staff turnover and to render psycho-emotional support in clinical medicine in general. Palliative care physicians face burnout, compassion fatigue and death anxiety. Whilst existing literature describes the benefit of formal mentoring programs and it's short to medium term goals, there	General	Singapore	Clinical and academic mentoring	Secondary	Qualitative	Systematic Review	Support of physicians	A total of 806 articles were retrieved from the initial search, on reading the full text to evaluate whether the articles fulfilled the selection criteria, a final number of 39 articles were chosen.	We identified four key themes from our analysis of the 39 papers included in this study. These include the mentoring relationship, the characteristics of the mentor, the characteristics of the mentee and advice to mentees and mentors in advancing effective mentoring relationships

		is a lack of data describing the relational aspects of mentoring and its long term goals in shaping a physician.<br><br>We aim to explore the perspectives of mentors and mentees featured in editorials, perspective and opinion pieces to lend a glimpse into the long term impact, the relational and personal aspects of mentoring									
Toh et al. 2017	Developing palliative care physicians through mentoring relationships.	Mentoring has been shown to improve resilience, sense of well-being, reduce staff turnover and to render psycho-emotional support in clinical medicine in general. Palliative care physicians face burnout, compassion fatigue and death anxiety. Whilst existing literature describes the benefit of formal mentoring programs and it's	Medical students and doctors	Not applicable	Not applicable	Secondary	Not applicable	Literature review	Mentoring relationship, the characteristics of the mentor, the characteristics of the mentee and advice to mentees and mentors in advancing effective mentoring relationships.	A total of 806 articles were retrieved from the initial search, on reading the full text to evaluate whether the articles fulfilled the selection criteria, a final number of 39 articles were chosen. We identified four key themes from our analysis of the 39 papers included in this study. These include the mentoring relationship, the characteristics of the mentor, the characteristics of the mentee and advice to mentees and mentors in advancing effective mentoring relationships.	Mentoring is an important source of support and growth for palliative physicians. The key themes identified in mentoring practice provides broad principles for allow integration of mentoring practice within prevailing palliative care practice and training.

		short to medium term goals, there is a lack of data describing the relational aspects of mentoring and its long term goals in shaping a physician.<br><br>We aim to explore the perspectives of mentors and mentees featured in editorials, perspective and opinion pieces to lend a glimpse into the long term impact, the relational and personal aspects of mentoring.									
Tomizawa et al. 2019	Role modelling for female surgeons in Japan	We founded the Japan association of women surgeons (jaws) with the aims to advance females in surgery by providing networking and to develop leadership, mentorship, education, expertise and advocacy. This article describes our philosophy and activities,	Medical students and physicians	Japan	Not indicated	Tertiary	Not indicated	Commentary	Not indicated	To find a single perfect role model is impossible in Japan, because lifestyle and family situations are quite different among individuals. Many young doctors in Japan find difficulties in identifying role models appropriate to their own situations.	Our concept of remote role modelling is to identify multiple outstanding persons with excellent roles that one wishes to emulate; classify them by field of work, lifestyle, family situations, and others; then select the parts that one needs and assemble them to customize one's own ideal role model. This type of role modelling is probably the most practical approach. While modern technology is good for communication, meeting role models in person at meetings and social

		and our concept of role modelling.									occasions is important in building an ideal role model.
Tran et al. 2014	West Yorkshire Mentor Scheme: teaching and development	The West Yorkshire Mentoring Scheme (WYMS) was created to provide a framework for clinical supervision, teaching and support by foundation year (FY) doctors for final-year medical students. Although established literature highlights the benefits of near-peer teaching, the accompanying mentoring role has little been explored. This study explored the impact of the WYMS for FY doctors and final-year medical students.	FY1 doctors, fifth year medical students	United Kingdom	West Yorkshire Mentoring Scheme (WYMS)	Primary	Mixed methods	Cohort study	Evaluation of scheme	Forty-nine medical students and 122 FY1s responded: 98 per cent of mentors and 100 per cent of mentees would recommend the scheme to their peers. Thematic analysis demonstrated that the scheme proved useful in skills development, teaching supervision and increasing preparedness for work.	WYMS is well received, beneficial and an excellent, local adjunct to clinical placements. It is of significant value to final-year students and their FY mentors, assisting in the development of student assistantships and clinical placement design. For FY doctors, it is a rewarding scheme that develops essential attributes of time management, communication and leadership for mentors and for the junior doctors who organise the scheme.
Trowbridge et al. 2010	The effect of overnight in-house attending coverage on perceptions of care and education on a general medical service	An increased emphasis on patient safety has led to calls for closer supervision of medical trainees. It is unclear what effect an	Residents, attending physicians	United States of America	Nil	Primary	Quantitative	Cross-sectional survey	Quality of care, educational experience	Of 58 total respondents, most faculty (91%) and resident (92%) physicians reported they were satisfied with the overall quality of care delivered and believed the quality of care delivered overnight	In-house attending coverage was acceptable to both residents and faculty, with perceived improvements in quality and educational experience.

		increased degree of faculty presence will have on educational and clinical outcomes. The aim of this study was to evaluate resident and attending attitudes and preferences regarding overnight attending supervision.								improved with an in-house attending system (90% and 85%, respectively). Most resident physicians (82%) believed the educational experience improved with the system of increased attending availability. Nearly all faculty (95%) and resident (97%) physicians preferred the in-house attending system to the traditional system of attendings being available by pager. The implementation of such coverage resulted in increased cost to the hospital for compensating covering hospitalist physicians.	
Usmani et al. 2011	Mentoring undergraduate medical students: experience from Bahria University Karachi	To explore the perceptions and the effects on mentors regarding mentoring medical students at Bahria University Medical and Dental College, Karachi, Pakistan.	Medical students	Bahria University Medical and Dental College, Pakistan	Formal program	Primary	Qualitative	Cross-sectional survey	Effects of mentoring, anonymous data collection tool	Close-ended questions reflected that majority of the mentors rate themselves as "good" or "satisfactory" mentors (55% and 45%).The mentors give enough time to their mentees and are comfortable with any gender or culture, and help their mentees settle down and improve them academically as well as personally (86.36%). Half the mentors sacrifice their personal time for the development of their mentees. All expressed personal satisfaction and fulfilment in observing	The perception of mentors about the structured mentoring programme subjectively appears to be a promising strategy for young medical students. Objective assessment of these mentors is needed.

										their mentees' success. Of all respondents, 86% ask mentees for regular feedback. Fifty nine percent mentors felt that their mentees showed good improvement academically due to these sessions, while 41% said that they were satisfied with the academic results of their mentees.	
Von der Borch et al. 2011	A Novel Large-scale Mentoring Program for Medical Students based on a Quantitative and Qualitative Needs Analysis.	Mentoring plays an important role in students' performance and career. The authors of this study assessed the need for mentoring among medical students and established a novel large-scale mentoring program at Ludwig-Maximilians-University (LMU) Munich School of Medicine.”	Medical students	Ludwig-Maximilians-University (LMU) Munich School of Medicine, Germany	Formal novel large-scale mentoring program	Primary	Mixed methods	Needs assessment	Overall satisfaction and desires for program.	Needs assessment revealed that 83% of medical students expressed overall satisfaction with the teaching at LMU. In contrast, only 36.5% were satisfied with how the faculty supports their individual professional development and 86% of students voiced a desire for more personal and professional support. When asked to define the role of a mentor, 55.6% "very much" wanted their mentors to act as counselors, arrange contacts for them (36.4%), and provide ideas for professional development (28.1%). Topics that future mentees "very much" wished to discuss included research (56.6%), final year electives (55.8%) and	Based on the strong desire for mentoring among medical students, the authors developed a novel two-tiered system that introduces one-to-one mentoring for students in their clinical years and offers society-based peer mentoring for pre-clinical students. One year after launching the program, more than 300 clinical students had experienced one-to-one mentoring and 1,503 students and physicians were involved in peer mentoring societies.

										experiences abroad (45.5%).	
Wasserstein et al. 2007	Mentoring at the University of Pennsylvania: Results of a faculty survey	To explore multiple aspects of mentoring at an academic medical center in relation to faculty rank, track, and gender	Faculty members	University of Pennsylvania, United States of America	Formal program	Primary	Quantitative	Cross-sectional survey	Self-administered survey developed from existing instruments and stakeholders	Most (92%) assistant and half (48%) of associate professors had a mentor. Assistant professors in the tenure track were most likely to have a mentor (98%). At both ranks, the faculty was given more types of advice than types of opportunities. Satisfaction with mentoring was correlated with the number of types of mentoring received ($r = .48$ and $.53$, $P < .0001$), job satisfaction ($r = .44$ and $.31$, $P < .0001$), meeting frequency ($r = .53$ and $.61$, $P < .0001$), and expectation of leaving the University within 5 years (Spearman $r = -.19$ and $-.18$, $P < .0001$), at the assistant and associate rank, respectively. Significant predictors of higher overall job satisfaction were associate rank [Odds ratio (OR) = 2.04, CI = 1.29-3.21], the 10-point mentoring satisfaction rating (OR = 1.27, CI = 1.17-1.35), and number of mentors (OR = 1.60, CI = 1.20-2.07).	Having a mentor, or preferably, multiple mentors is strongly related to satisfaction with mentoring and overall job satisfaction. Surprisingly, few differences were related to gender. Mentoring of clinician-educators, research track faculty, and senior faculty, and the use of multiple mentors require specific attention of academic leadership and further study.
Wazen et al. 2020	Tales of mentoring in	To investigate the utility of	Residents	United States of America	Dyadic Group	Primary	Qualitative	Cross-sectional interview	Experience and opinion of	During this experience all mentors had a	Mentoring groups can be a valuable addition to

	radiology: the experience of residents and mentors at a single academic program	mentoring groups in radiology residency.							mentor and residents	positive experience. The majority found it helpful to have residents of different levels in their group to allow for peer to peer mentoring and all thought the mentoring program should continue. The most common topics they covered during the sessions were career advice and specialty choice. Almost all mentees had a positive or very positive mentoring experience this year. The high majority found that having residents of different levels was beneficial. Topics that mentoring sessions helped mostly with were career advice, work life balance and study skills.	residency training, especially in helping with career advice and work life balance.
Wear and Skillicorn, 2009	Hidden in plain sight: The formal, informal, and hidden curricula of a psychiatry clerkship	To examine perceptions of the formal, informal, and hidden curricula in psychiatry as they are observed and experienced by (1) attending physicians who have teaching responsibilities for residents and medical students, (2) residents who are taught by those same	Attendings, residents and medical students	Not indicated	Not indicated	Primary	Qualitative	Cross-sectional	Explored perceptions and experiences with various enactments of the formal, informal, and hidden curricula reflected in the teaching environment of the psychiatry department	All three groups offered a similar belief that the knowledge, skills, and values of the formal curriculum focused on building relationships. Similarly, all three suggested that elements of the informal and hidden curricula were expressed primarily as the values arising from attendings' role modeling, as the nature and amount of time attendings spend with patients, and as attendings' advice	The process described here has great potential in local settings across all disciplines. Asking teachers and learners in any setting to think about how they experience the educational environment and what sense they make of all curricular efforts can provide a reality check for educators and a values check for learners as they critically reflect on the meanings of what they are learning.

		physicians and who have teaching responsibilities for medical students, and (3) medical students who are taught by attendings and residents during their psychiatry rotation.								arising from experience and intuition versus “textbook learning.” Whereas students and residents offered negative values arising from the informal and hidden curricula, attendings did not, offering instead the more positive values they intended to encourage through the informal and hidden curricula.	
Wetter 2020	Personal reflections on mentorship as a pathway toward sustaining a joyful dermatologic practice: part i: influential mentors during training and early career	Not indicated	Physicians	United States of America	Mentoring	Tertiary	Not indicated	Commentary	Not indicated	The greatest mentors model career paths that are personally fulfilling and intellectually stimulating, provide tangible guidance and boundless encouragement to help mentees discern and achieve their goals, and inspire self-confidence in mentees that translate to success in a variety of areas, including patient care, education, research, and overall life purpose.	Mentorship is a readily available resource to all of us and embodies many of the qualities that initially attracted us to the medical profession. Helping others to achieve their goals is inherently joyful and immensely gratifying, and it provides clarity of purpose that we are part of a community greater than ourselves.
Wingard et al. 2004	Facilitating faculty success: Outcomes and cost benefit of the UCSD National Center of Leadership in Academic Medicine	The study assessed four primary outcomes associated with participation in the UCSD NCLAM program: whether participants stayed at UCSD, whether they stayed in	Faculty members	University of California San Diego, United States of America	Formal structure mentoring program	Primary	Quantitative	Pre-post study survey	Whether participants stayed at UCSD, whether they stayed in academic medicine, a quantitative assessment of improved confidence in skills needed to	Among 67 participants, 85% remained at UCSD and 93% in academic medicine. Their confidence in skills needed for academic success improved: 53% personal leadership, 19% research, 33% teaching, and 76% administration. Given improved retention rates, savings in recruitment	Structured mentoring can be a cost-effective way to improve skills needed for academic success and retention in academic medicine.

		academic medicine, a quantitative assessment of improved confidence in skills needed to succeed in academic medicine, and costs of the program compared to dollars spent on junior faculty recruitment.							succeed in academic medicine, and costs of the program compared to dollars spent on junior faculty recruitment.	was greater than cost of the program.	
Wolff et al. 2020	Coaching to improve self-directed learning	The continuously changing health care context necessitates that medical trainees develop self-directed learning skills. This study examined the effect of coaching on the self-directed learning process in pre-clerkship medical students.	Medical students	United States of America	Group	Primary	Quantitative	Randomised control trial	Implementation of learning goals	Of 171 students enrolled, 167 completed all four assessments and were included. All 167 developed a learning goal after each assessment. Of students receiving coaching, 91.8% implemented a learning goal, whereas only 65.9% of students in the control group implemented a learning goal. Students who received coaching were more likely to incorporate performance feedback into their learning goals.	For students, faculty member coaching facilitated better learning goal development and more frequent implementation compared with students who did not receive coaching.
Wolff et al. 2020	Academic coaching: insights from the medical student's perspective	With the growing recognition of the role of coaching in competency-based medical education, many medical education training programs are	Medical students	United States of America	Group	Primary	Qualitative	Cross-sectional interview	Students' perceptions of successful coaching experiences: coach attributes, relational skills, coaching skills, and utilization of	With the growing recognition of the role of coaching in competency-based medical education, many medical education training programs are investing significant resources into developing coaching	The findings from this study illuminate the student stakeholder perspectives on successful coaching relationships, and should be considered when developing a coaching program and faculty development.

		investing significant resources into developing coaching programs. However, there is a lack of rigorous research on academic coaching programs in medical education and recommended coaching practices are based on expert opinion without incorporating the student perspective. The aim of this study was to investigate factors that affect a students' perception of a successful coaching experience.							coaching. Each of these themes had specific dimensions.	programs. However, there is a lack of rigorous research on academic coaching programs in medical education and recommended coaching practices are based on expert opinion without incorporating the student perspective. The aim of this study was to investigate factors that affect a students' perception of a successful coaching experience.	
Wuensch et al. 2011	Disclosing information about randomised controlled trials in oncology: training concept and evaluation of an individualised communication skills training for physicians COM-ON-rct	When physicians disclose information about randomised controlled trials, they have to balance the requirements of conducting high standard research and the respect for patients' rights. Physicians need	Physicians treating oncology patients	Germany	Group	Primary	Quantitative	Cross-sectional survey	Not indicated (Evaluation of training program)	Forty physicians have been trained. The acceptance of the training concept was assessed by a questionnaire consisting of 14 items and using a 6-point scale from 1 (very best) to 6 (very bad): the individualised CST was highly accepted (mean = 1.33). Practicing with actor-patients (mean = 1.4),	Our CST trains physicians to realise best research standards and incorporate patients' rights

		training in this difficult matter. An individualised communication skills training (CST) about randomised controlled trials for oncologists has been developed. The aim of this publication is to describe the concept of our CST and present data of evaluation by the participants:								providing constructive feedback (mean = 1.3) and assessing individual learning goals (mean = 1.85) were seen as helpful.	
Yedavalli et al. 2019	Resident' perceptions of usage of the current alumni and attending network for a formal mentorship program in an academic affiliated community hospital radiology residency	Mentor-mentee relationships within radiology residencies can add significant value to a resident's overall experience. Studies demonstrate that mentorship programs can increase satisfaction for residents and faculty alike by reducing stress, easing career related decisions, increasing involvement with research, improving teaching and communication	Resident	United States of America	Informal program	Primary	Quantitative	Cross-sectional survey	Pre-mentor and post-mentor satisfaction survey	The overall response rate for the mentor survey was 35% (28/81) with a positive response rate, defined as a yes response to the mentorship program, of 30% (24/81). The positive response rate group was comprised of 20 male and 4 female respondents. External and internal alumni response rates were 27% (16/59) and 45% (8/11), respectively. The internal attending response rate was 45% (10/22). The mentee survey resulted in 100% overall and positive response rates (13/13). A positive response was defined as a resident	Our results demonstrate overall positive resident perceptions of the formalized program using our existing alumni network, both internal and external, in addition to our current faculty. We show statistically significant increased satisfaction with faculty engagement, overall morale, research involvement, networking for both fellowship and job prospects, as well as reducing stress. We show no significant changes in resident perceptions due to the formal program with respect to satisfaction with recruitment, communication skills, and leadership roles.

		skills, and finally increasing leadership roles.								interested in having a mentor.	
You et al. 2019	Outcomes of mentoring programs in psychiatry training: a literature review	This literature review aimed to outline the evidence regarding the outcomes of formal mentoring programs for training psychiatrists	Training physicians	New South Wales, Australia	Formal mentoring programs	Secondary	Mixed methods	Systematic review	Not indicated	A total of 17 articles fulfilled inclusion criteria. Positive outcomes of mentorship include promotion of research, perpetuation of further mentorship, increased perceived support and enhancement of teaching skills. One article reported a negative outcome.	Current evidence regarding mentorship in psychiatry is limited and there is a lack of clarity regarding the relationship between positive outcomes and mentorship. Components of the included programs may act as confounders. Mentorship, protected teaching time, and didactic teaching may be important to encourage research. Other potential benefits of mentorship include enhanced career guidance, perceived support, and enriched teaching skills.
Yung et al. 2019	Do teaching strategies matter? Relationships between various teaching strategies and medical students' wellbeing during clinical workplace training	In the later years of medical school, medical students learn through clinical rotations at medical institutions. Using cognitive apprenticeships as the theoretical reference for teaching strategies, this study aimed to assess how clinical teaching strategies benefit medical students' wellbeing in the workplace.	Medical students	Taiwan	Clinical training	Primary	Quantitative	Cross-sectional survey	Teaching strategies and personal wellbeing	We determined that the inspiring teaching strategy, characterized by articulation, reflection, and exploration, was related to reduced burnout among medical students and an increased sense of compassion satisfaction; the directing teaching strategy, characterized by modeling, coaching, and scaffolding, was related only to reduced burnout among medical students but not to compassion satisfaction during the clinical training.	Clinical teaching strategies were demonstrated to affect, to various extents, medical students' wellbeing with respect to factors such as burnout and compassion satisfaction in the workplace. Clinical teachers and educators should increase efforts to develop inspiring teaching skills to shift the balance of responsibility and to support students in the teaching and learning relationship.
Zuzuarregui and Hohler 2015	Comprehensive opportunities for research and	We developed a program to promote medical	Medical students	United States of America	Formal program	Primary	Qualitative	Retrospective study	Residency application uptake	Since the implementation of this program in 2010, the	In this mentoring program, students are prepared for residency application and

	teaching experience (CORTEX): a mentorship program.	student interest in pursuing a career in neurology. This program focuses on medical student mentorship. It also offers opportunities in teaching and clinical research in order to provide students with marketable skills for an academic career in neurology.								number of students entering into the field of neurology from our institution significantly increased from 14 students between 2006 and 2010, to 30 students between 2011 and 2014 ($p < 0.05$). Medical student research productivity increased from 7 publications during 2006-2010, to 22 publications, 14 poster presentations, and a book chapter after implementation of this program in 2010 ($p < 0.05$).	provided with research and teaching opportunities. Students develop a highly desirable academic skill set for residency and have matched at top-ranked institutions. This program has been successful in improving student productivity in clinical research and garnering student interest in neurology.
